# Ultrahigh-molecular-weight tadpole polymers with statistically symmetric head-to-tail geometry: the key topological intermediate bridging linear and cyclic architectures

**DOI:** 10.1039/d6sc04605c

**Published:** 2026-08-11

**Authors:** Wendi Liang, Jun Gong, Yongkang Deng, Wenjie He, Yiren Wang, Mo Zhu, Xin Guan, Zhi-Chao Yan, Zhi Chen, Xiaopeng Li, Yu Song, Lianwei Li, Wenke Zhang

**Affiliations:** a College of Chemistry and Environmental Engineering, Shenzhen University Shenzhen 518060 China xinguan@szu.edu.cn zhi.chen@szu.edu.cn lianweili@szu.edu.cn; b State Key Laboratory of Supramolecular Structure and Materials, College of Chemistry, Jilin University Changchun 130012 China; c School of Chemical Engineering and Light Industry, Guangdong University of Technology Guangzhou 510006 China; d State Key Laboratory of Organometallic Chemistry, Shanghai Institute of Organic Chemistry, University of Chinese Academy of Sciences Shanghai 200032 China

## Abstract

Polymer topology profoundly influences material properties by regulating chain arrangement, conformational entropy, and interactions, yet understanding topological entanglement constraints like threading and knotting remains a fundamental challenge in polymer science, partly due to the scarcity of structurally precise, ultrahigh-molar-mass ring-containing polymers as model systems. Herein, we address this synthetic challenge with a dual-terminal intramolecular cyclization strategy to synthesize tadpole polymers, the key architectural intermediate bridging linear and monocyclic topologies. This approach leverages synergistic dual-end groups and viscoelastic effects to maintain chain-end functionality, enabling efficient synthesis of tadpole polymers with ultrahigh molar masses (0.4–1.0 MDa) and statistically symmetric head-to-tail geometry. Such precise structural control facilitates direct visualization of individual tadpoles *via* advanced microscopy techniques. Physical characterization shows their distinct properties: an average ∼10% reduction in intrinsic viscosity (signaling compact packing) while the fractal dimension (*d*_f_ ≈ 1.7) remains unchanged; in the melt, rheology exhibits an entanglement plateau extended by 1–2 orders of magnitude, indicative of long-lived, threading-stabilized transient networks. Additionally, we develop a versatile post-polymerization strategy to build functional tadpole polymer libraries. This work provides a valuable experimental platform for examining the linear-to-cyclic topological transition and establishes a robust platform for investigating constrained dynamics and engineering advanced soft materials in future work.

## Abstract 2

A dual-terminal cyclization strategy enables synthesis of ultrahigh-molecular-weight symmetric tadpole polymers. These well-defined hybrids exhibit unique melt viscoelasticity and support modular functionalization for topological polymer studies.

## Graphical abstract

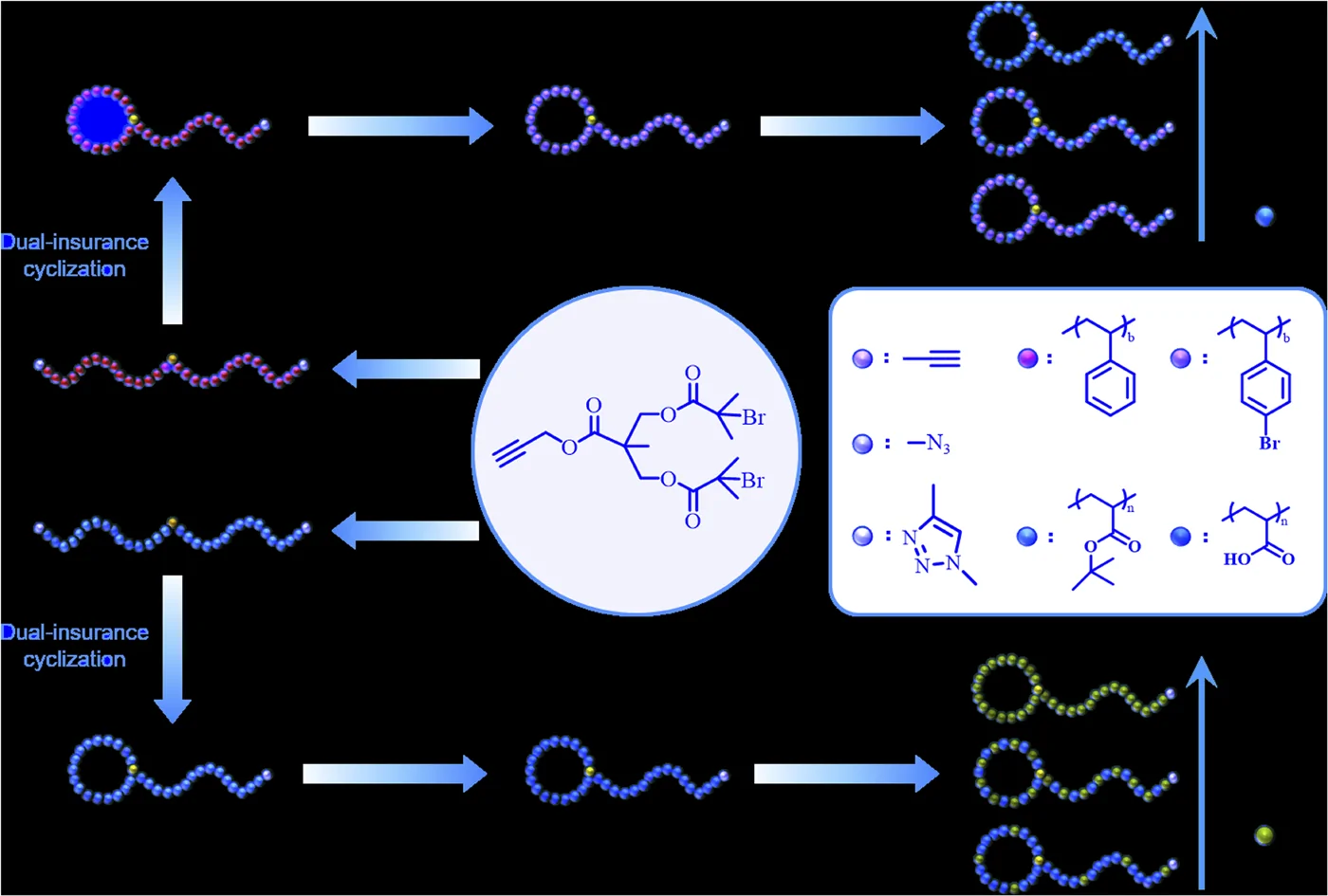

## Introduction

Polymer topology serves as a fourth-dimensional structural parameter, governing material properties by precisely regulating chain arrangement, conformational entropy, and entropy–enthalpy balance.^1–3^ For a given chemical composition, it reprograms macroscopic physical characteristics through exact control over molecular motion and interactions, enabling promising applications such as flexible electronics,^4^ drug delivery,^2^ biocompatible interface materials,^5^ and luminescent materials.^6^ Among diverse topological architectures, tadpole polymers stand out as a unique architectural intermediate that intrinsically combines linear and cyclic segments within a single molecule, imposing enhanced topological constraints such as threading, knotting, and entanglement.^7–9^ This hybrid structure endows tadpole polymers with distinctive physicochemical properties, including suppressed reptation dynamics in the cyclic head and free-end motion in the linear tail, which can lead to novel behaviors like anomalous viscoelasticity and rheological properties.^7^

Despite their potential, the precise synthesis of tadpole polymers remains highly challenging, primarily due to low cyclization efficiency and the difficulty in ensuring the coexistence of well-defined linear tails.^10^ Previous efforts based on intramolecular coupling strategy generally suffer from limited efficiency imposed by high-dilution requirements to minimize intermolecular reactions, as well as molecular weight constraints that prevent the formation of large rings without significant byproducts like dimers or multimers.^2,11^ Ring-expansion polymerization offers an alternative for large rings but is limited by monomer and catalyst compatibility, often yielding products with insufficient molecular weight or structural uniformity to probe pronounced topological effects.^12–14^ Furthermore, even with successful cyclic precursors, precise control over linear tail attachment is crucial to suppress competing side reactions, notably self-cyclization of the linear tail, which can lead to undesired monocyclic structures.^15^ Most importantly, these synthetic barriers have historically limited tadpole polymers to molar masses below *ca.* 100 kDa, with head (ring) and tail (linear) segments each below *ca.* 50 kDa, which are too low to exhibit pronounced topological effects.^16^

Over recent decades, due to these synthetic limitations of tadpole polymers, researchers have adopted physically blended systems of linear and cyclic polymers to simulate macroscopic properties arising from linear–cyclic topological interactions.^9,17^ In weakly correlated polymer blends, topological constraints in ring polymers have proven significantly more complex to characterize than in their linear counterparts.^18–21^ Recent studies have demonstrated that ring-linear polymer blends exhibit distinct rheological properties, such as enhanced viscosity compared to individual components, owing to differences in knotting and entanglement.^17,22^ This emergent behavior arises from long-lived topological constraints, wherein linear polymers thread the ring polymers, creating transient networks that alter stress relaxation and flow dynamics.^23^ Significant research progress has been made in ring-linear polymer blends, particularly in the nonlinear rheometric behavior,^18^ polymorphism of ring components,^22^ and formation of topological bigels.^23^ However, most advances remain limited to theoretical and computational studies, as direct experimental observation of threading or de-threading processes is challenging. This stems not only from the transient nature of interactions in blends but also from the scarcity of structurally precise, ultrahigh-molar-mass cyclic polymers available in sufficient quantities.^24–26^

To complement blend and simulation studies, covalently bonded tadpole polymers offer a well-defined system for experimental validation. Their intrinsic architecture provides a well-defined experimental platform for quantifying topological effects, bridging the gap between linear and cyclic behaviors while allowing systematic control over interactions that are fleeting in blends. Nevertheless, the effect of molecular weight on their properties remains critically important. As classic studies on cyclic polymers have revealed, intramolecular entanglements can only form when the chain length is sufficiently large, thereby imparting higher toughness, thermal stability, and melt processability to the material.^12,14,27^ Despite advances in synthetic methods such as intramolecular cyclization, intermolecular coupling, and electrostatic assembly, the field has been hampered by molecular weight limitations.^2,28,29^ Equally important, the permanently coupled ring–linear motif renders tadpole polymers a tractable building block for constructing more complex mechanically interlocked and slide-ring polymer architectures, which further motivates the pursuit of high-molar-mass, structurally defined samples.^30^

Herein, we present a dual-terminal intramolecular cyclization strategy to address these synthetic barriers. This strategy offers three distinct advantages over conventional methods: (i) it exploits built-in redundancy from the dual-end groups, combined with viscoelastic effects, to preserve chain-end functionality and favor predominant tadpole polymer formation, thereby enabling efficient cyclization even at ultrahigh molar masses; (ii) the design of AB_2_-type linear precursors enables the synthesis of statistically symmetric tadpole PS and poly(*tert*-butyl acrylate) (PtBA) (*t*-PS_*n*_ and *t*-PtBA_*n*_) with well-matched head-tail dimensions; (iii) this cyclization strategy suppresses intermolecular coupling, yielding tadpole polymers with molar masses up to ∼0.4 MDa (PS) and ∼1.0 MDa (PtBA) without detectable dimer byproducts, extending beyond previously reported sub-100 kDa systems. Beyond this synthetic advance, we leverage these samples to uncover their topology-dependent properties in both solution and melt states, and develop versatile post-polymerization strategies for constructing functional tadpole polymer libraries. This work establishes an experimental platform for investigating constrained dynamics and expanding the application scope of topological polymers in future work.

## Results and discussion

We initiated our tadpole polymer synthesis by designing an AB_2_-type functional atom transfer radical polymerization (ATRP) initiator, featuring one central alkyne and two terminal bromine groups, to prepare well-defined linear precursors (*l*-PS_*n*_ and *l*-PtBA_*n*_) *via* activators regenerated by electron transfer (ARGET) ATRP (Scheme 1a). A fundamental limitation in conventional tadpole synthesis is the inevitable decay of bromine end-group fidelity at ultrahigh molar masses (>300 kDa) due to radical termination.^31^ Our dual-terminal cyclization strategy mitigates this limitation by statistical redundancy. Namely, even with a 70% single-end retention rate, the probability of both chain ends being deactivated is only 9%, —i.e., 91% of chains retain at least one functional end and can undergo cyclization. While this does not eliminate precursor heterogeneity (chains with zero, one, or two active ends coexist), it substantially increases the fraction of chains capable of subsequent azidation and intrachain CuAAC cyclization, which is important for accessing high-molar-mass species. To precisely correlate reaction parameters with product structure, we engineered a high-throughput cyclization reactor with quantitative injection control protocol (Scheme 1b). It is worth noting that at elevated concentrations, intermolecular coupling becomes non-negligible, competing with intramolecular cyclization and generating multimeric and hyperbranched byproducts (Scheme 1c). Actively suppressing these side reactions was therefore a major focus of our optimization.

**Scheme 1:**
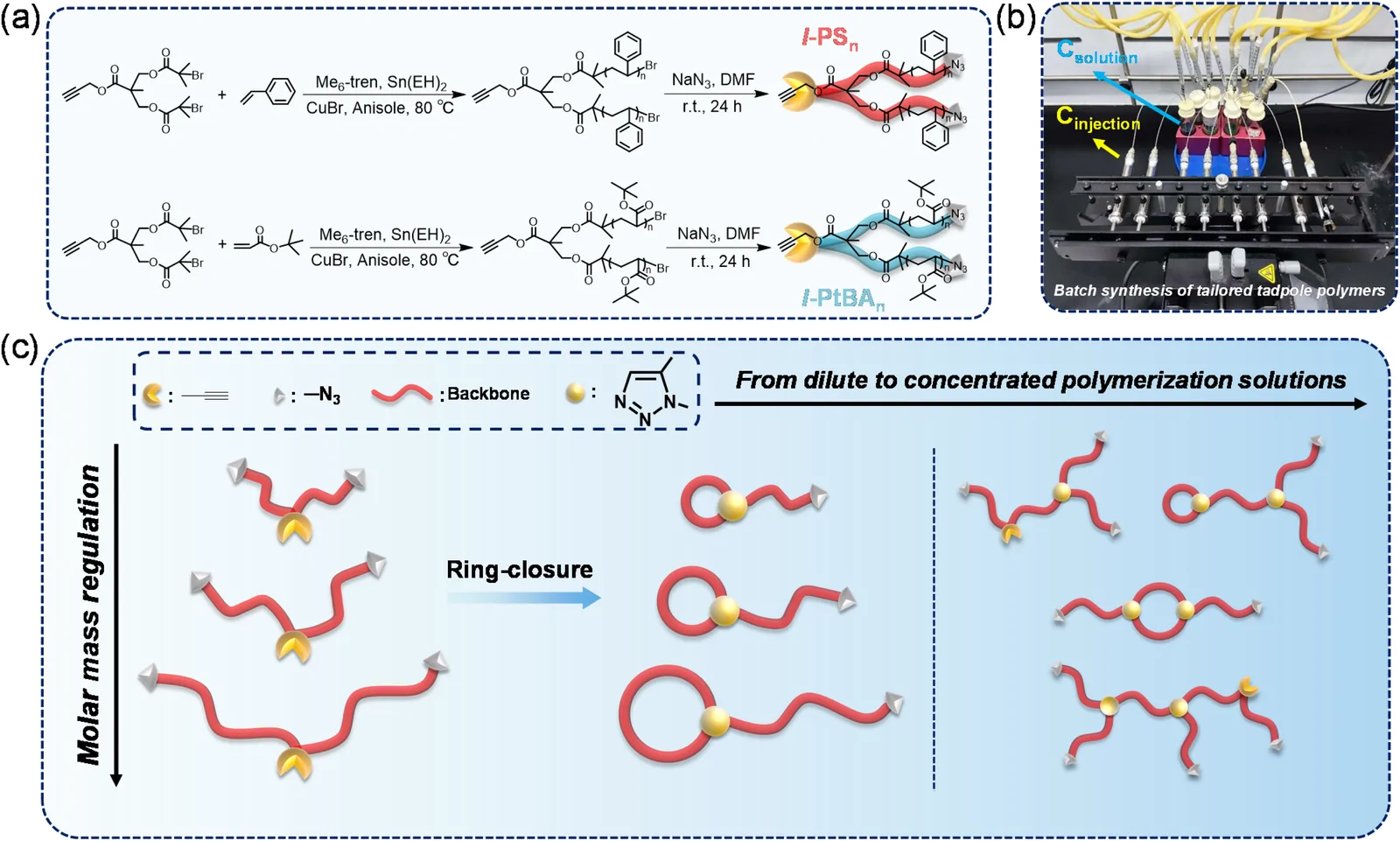
A dual-terminal cyclization strategy for topologically defined ring-containing tadpole polymers. (a) Precise synthesis of telechelic AB_2_-type linear precursors (*l*-PS_*n*_/*l*-PtBA_*n*_) with central alkyne and terminal azides. (b) High-throughput experimental protocol for architectural control of tadpole products (*t*-PS_*n*_/*t*-PtBA_*n*_). (c) Potential competition between intrachain cyclization and interchain coupling for AB_2_-type linear precursors by CuAAC click chemistry.

To achieve optimal molecular control and suppress radical termination, ARGET ATRP of PS in anisole and PtBA in acetone was conducted below *T* = 80 °C and 70 °C, respectively, with monomer conversions maintained at 20–40% (see Tables S1 and S2 for detailed conditions). This protocol yielded a series of narrowly dispersed linear precursors, *l*-PS_*n*_ (56 ≤ *n* ≤ 3766) and of *l*-PtBA_*n*_ (63 ≤ *n* ≤ 7936), with maximum molar masses reaching ∼0.4 MDa and ∼1.0 MDa, respectively. Representative *t*-PS_1633_ and *t*-PtBA_1587_ syntheses were reproduced in three independent 500 mg batches, giving mean isolated yields of 90.5 ± 2.4% and 88.0 ± 1.4%, respectively (Table S3). Both relative and absolute molar masses (M_rel_ and M_abs_) were determined by conventional and quadruple-detection GPC (QD-GPC) (Tables S4–S7). For the ultralow-molar-mass precursors *l*-PS_56_ and *l*-PtBA_63_, comprehensive characterization by ^1^H nuclear magnetic resonance (^1^H NMR), Fourier transform infrared (FTIR) spectroscopy, and matrix-assisted laser desorption/ionization time-of-flight mass spectrometry (MALDI-TOF MS) of their bromo- and azide-terminated analogues provided detailed validation (Fig. 1a–f): (i) ^1^H NMR indicated quantitative bromine-to-azide conversion (>95%) *via* the characteristic shift of the methylene signal, while the central alkyne proton remained intact; (ii) FTIR showed the diagnostic azide stretch at 2092 cm^−1^; (iii) MALDI-TOF MS confirmed repeat unit spacings and revealed azide-terminal dissociation fragments, aligning well with theoretical structures (Fig. S1 and S2).

**Fig. 1:**
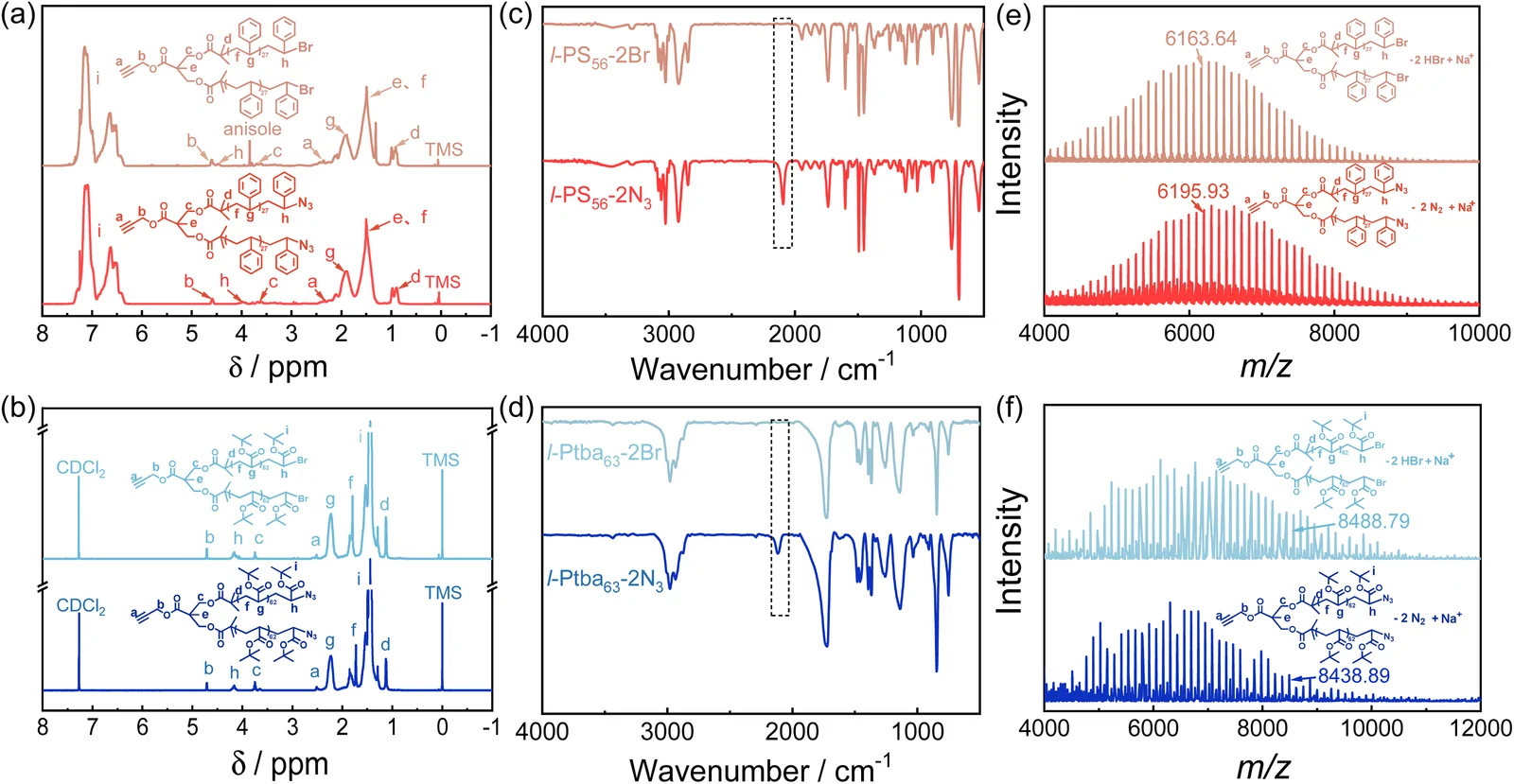
Structural confirmation of telechelic AB_2_-type linear precursors. (a and b) ^1^H NMR spectra, (c and d) FTIR spectra, and (e and f) MALDI-TOF MS of bromo- and azide-terminated linear precursors PS_56_ (*l*-PS_56_-2Br, *l*-PS_56_-2N_3_) and PtBA_63_ (*l*-PtBA_63_-2Br, *l*-PtBA_63_-2N_3_).

The solution conformation of the linear precursors was first established by dynamic light scattering (DLS). Unimodal f(*R*_h_) distributions for *l*-PS_*n*_ (56 ≤ *n* ≤ 3766) exhibited a scaling relationship of *R*_h_ ∼ *M*_w_^0.56^, with *R*_h_ increasing from ∼1.9 nm to ∼19.1 nm (Fig. 2a), consistent with established polymer solution theory.^32^ In contrast, the *l*-PtBA_*n*_ (63 ≤ *n* ≤ 7936) displayed a lower scaling exponent (*R*_h_ ∼ *M*_w_^0.47^) (Fig. 2b). Cyclization was assessed by complementary techniques. For low-molar-mass precursors (*e.g.*, *l*-PS_56_ and *l*-PtBA_63_), the disappearance of azide stretch in FTIR, the emergence of triazole protons in ^1^H NMR, and MALDI-TOF MS were consistent with the expected cyclized structures (Fig. S3–S6). For higher-molar-mass species, GPC refractive index (RI) signals revealed significant peak shifts to higher elution volumes for all precursors (Fig. 2c and d and S7), consistent with a reduction in hydrodynamic volume upon cyclization. The ratio of apparent peak molar masses (*M*_rel,p,t_/*M*_rel,p,l_) for the tadpole products fell in the range of 0.82–0.92 (Fig. 2e and f), a value notably higher than that for monocyclic polymers (0.75–0.80).^33,34^ These chromatographic results reflect a characteristic hydrodynamic response consistent with the tadpole architecture, in which the cyclic head imposes compactness while the unconstrained linear tail provides hydrodynamic compensation. However, this result does not exclude the presence of a mixture containing linear and cyclic species. Notably, the GPC peak shift (Δ*V*_retention_ = 0.08–0.10 mL) of ultrahigh-molar-mass *t*-PS_3766_ after cyclization was subtle yet reproducible (Fig. S8), consistent with the feasibility of our cyclization strategy for high-molar mass species. Across five replicate injections, *l*-PS_3766_ and *t*-PS_3766_ gave mean retention volumes of 19.468 ± 0.018 and 19.554 ± 0.020 mL, respectively, corresponding to a mean shift of 0.086 ± 0.009 mL (Table S8).

**Fig. 2:**
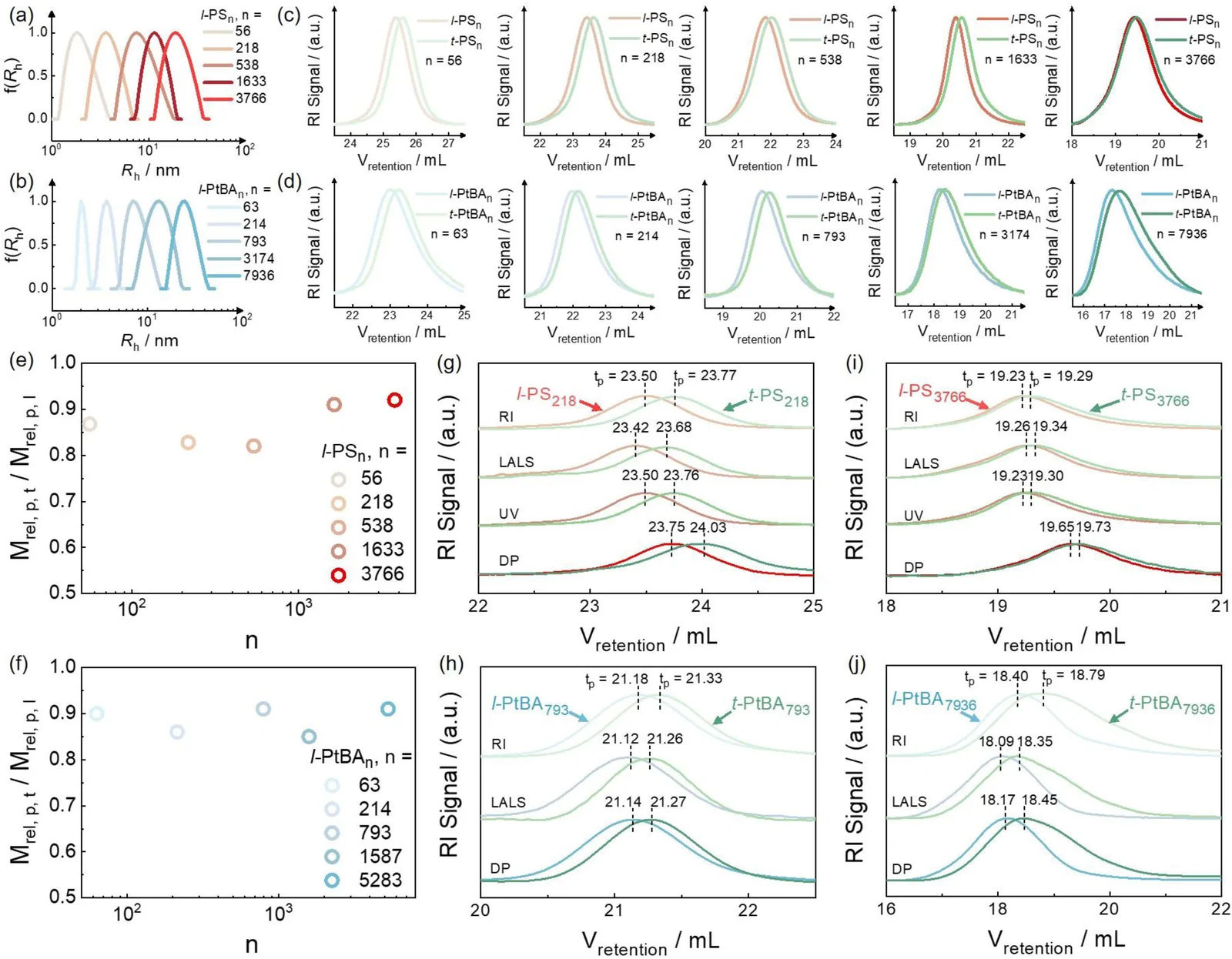
Chromatographic evidence of topology-dependent conformational contraction upon cyclization. (a and b) Hydrodynamic radius (*R*_h_) distributions and scaling relationships for AB_2_-type linear precursors (*l*-PS_*n*_ and *l*-PtBA_*n*_) in solutions. (c and d) Conventional/QD-GPC traces for linear precursors (*l*-PS_*n*_ and *l*-PtBA_*n*_) and their corresponding tadpole counterparts (*t*-PS_*n*_ and *t*-PtBA_*n*_) using THF as the eluent. (e and f) Apparent peak molar mass ratios (*M*_rel,p,t_/*M*_rel,p,l_) as a function of degree of polymerization for linear precursors and their tadpole counterparts. (g–j) Comparative QD-GPC traces (RI, UV, LALS, DP signals) for representative linear precursors and their tadpole counterparts (PS_218_, PS_3766_, PtBA_793_, and PtBA_7936_).

Additionally, the topological transitions from linear precursors to tadpole polymers were further supported by QD-GPC on account of the peak shift in differential RI, ultraviolet (UV), low angle light scattering (LALS), and viscosity (DP) signals (Fig. 2g–j and S9–S10). Compared with *t*-PS_3766_, ultrahigh-molar-mass *t*-PtBA_7936_ with an apparent weight-average molar mass beyond ∼1.0 MDa even displayed more pronounced peak shifts (Fig. 2j), suggesting a more pronounced topology-dependent contraction for the more flexible PtBA backbone. The ultralong contours of the head-tail segments were estimated to be approximately 565 nm for *t-*PS_3766_ and 1190 nm for *t*-PtBA_7936_, significantly beyond the required molar mass for interchain topological entanglement in the melt state.^35^

We further systematically investigated the dependence of intramolecular cyclization efficiency for *t*-PS_*n*_ series on injection concentration (*C*_i_) and final solution concentration (*C*_s_) using our high-throughput reactor (experimental conditions summarized in Table S9). Analysis of GPC peak shifts and deconvolution quantification revealed a pivotal concentration window for high-purity synthesis. For *t*-PS_*n*_ with *n* ≤ 1633 (Fig. S11), *C*_i_ ≤ 18 g L^−1^ (*C*_s_ ≈ 1.64 g L^−1^) yielded stable *M*_rel,p,t_/*M*_rel,p,l_ ratios of 0.80–0.94 with no detectable dimer formation (Fig. S12), confirming the dominance of the desired intramolecular pathway under dilute conditions. At elevated concentrations (*C*_i_ = 54 g L^−1^), the emergence of shoulder peaks in GPC profiles and an increase in the molar mass ratio for longer chains (*n* ≥ 218) indicated the onset of intermolecular coupling (Fig. S11), a consequence of reduced end-group segregation at lower overlap concentrations (C*). QD-GPC analysis further identified the primary byproduct at *C*_i_ = 162 g L^−1^ as dimeric species (Fig. S13 and Table S10). For the ultra-long *l*-PS_3766_ chains, successful cyclization was achieved within an operational window (*C*_i_ = 6–18 g L^−1^) that notably exceeded the upper concentration limits reported for most monocyclic systems. This expanded processing range is attributed to the steric protection of the central alkyne and the chain's viscoelastic properties.^36^ Specifically, the central alkyne buried in the polymer coil requires intermolecular azides to penetrate a high-density region, whereas intramolecular azide can circumvent this entropic penalty *via* local conformational fluctuations. Meanwhile, the exceptionally long relaxation times of ultrahigh-molecular-weight chains (*τ* ∼ *M*^2^–*M*^3^) kinetically trap the central alkyne and the terminal azides in close proximity over the typical reaction timescale. This persistent spatial proximity strongly promotes intramolecular azide–alkyne encounters in preference to intermolecular collisions, as the latter necessitate two independent chains to diffuse over distances far exceeding the chain size.

The topological impact of cyclization was further quantified by comparing the intrinsic viscosity ([*η*]) and melt rheology of tadpoles with their linear precursors. QD-GPC analysis revealed consistent reductions in [*η*] for the tadpole products at equivalent elution volumes (Fig. 3a and b and S14–S15), with average decrements of ∼10.8% for *t*-PS_*n*_ and ∼9.4% for *t*-PtBA_*n*_, suggesting more compact chain packing. This compaction was corroborated by a correspondingly smaller radius of gyration (*R*_g_) for the tadpoles (Fig. S16). Notably, the [*η*]–*M* scaling relationships yielded identical fractal dimensions (*d*_f_ = 1.76, where *d*_f_ = 3/(*α* + 1) = 1.76 with (*α* ≈ 0.7)) for both linear and tadpole architectures (Fig. 3c and d). However, the [*η*]_t_/[*η*]_l_ ratios (0.82–0.95) substantially exceeded those of monocyclic polymers (0.57–0.63),^37^ a signature of the hydrodynamic volume compensation by the linear tail in this hybrid topology.

**Fig. 3:**
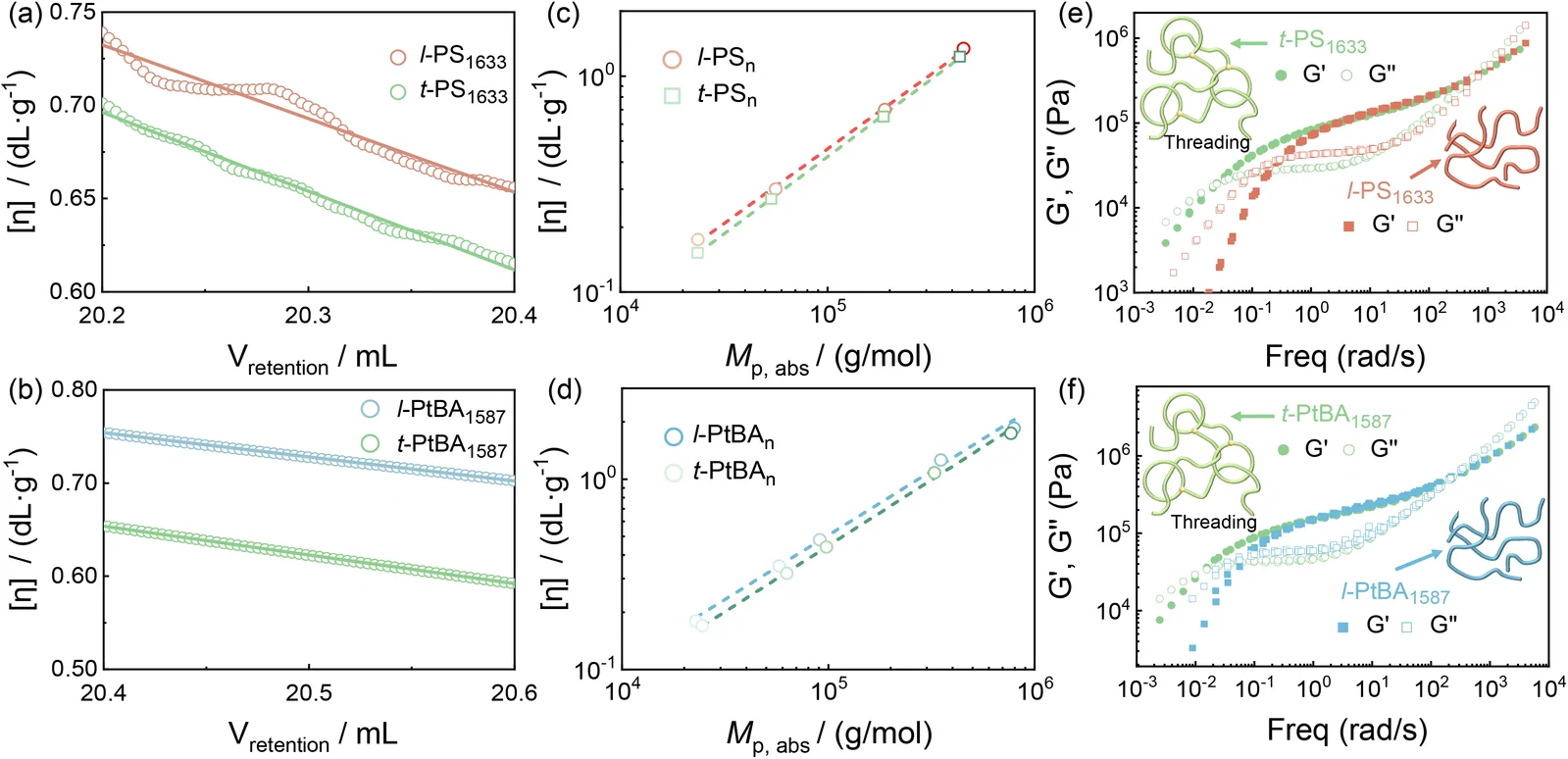
Unique topological properties of high-molar-mass tadpole polymers in solution and melt states. (a and b) Intrinsic viscosity ([*η*]) *versus* elution volume for linear precursors (*l*-PS_1633_ and *l*-PtBA_1587_) and their corresponding tadpole counterparts (*t*-PS_1633_ and *t*-PtBA_1587_). (c and d) [*η*]–M (absolute molar mass) scaling relationships for linear precursors and their corresponding tadpole counterparts in solutions. (e and f) Rheological properties showing frequency dependence of storage (*G*′) and loss (*G*″) moduli for linear precursors (*l*-PS_1633_, *l*-PtBA_1587_) and their corresponding tadpole polymers (*t*-PS_1633_ and *t*-PtBA_1587_). Data were obtained from frequency sweeps at different temperatures (70–130 °C) and horizontally shifted along the frequency axis to construct time–temperature superposition master curves.

More profound effects were observed in the melt state. Rheological frequency sweeps demonstrated that *t*-PS_1633_ and *t*-PtBA_1587_ significantly extended the entanglement plateau compared to their linear analogs. Specifically, for the PS series, the low-frequency edge shifted from 7.0 × 10^−1^ rad s^−1^ for *l*-PS_960_ to 2.0 × 10^−1^ rad s^−1^ for *l*-PS_1633_ and further to 2.0 × 10^−2^ rad s^−1^ for *t*-PS_1633_. For the PtBA series, the corresponding edge shifted from 7.0 × 10^−2^ rad s^−1^ for *l*-PtBA_1587_ to 1.0 × 10^−2^ rad s^−1^ for *t*-PtBA_1587_ (Fig. 3e and f). Relative to the molecular-weight-matched linear precursors, these values correspond to a one-order-of-magnitude shift for PS and an approximately sevenfold shift for PtBA. This dramatic extension of the relaxation spectrum is consistent with the formation of long-lived, threading-stabilized transient networks. We attribute this emergent behavior to the unique topology of the tadpoles, where the cyclic head acts as a robust slip-ring, and the linear tail can thread either its own ring or those on adjacent molecules. This self-threading or inter-molecular threading mechanism not only increases the effective entanglement density but also creates a topology that can potentially enhance both melt elasticity and stretchability. These results are qualitatively consistent with the long-lived threading constraints predicted for ring–linear systems, while the covalent head–tail linkage permits the effect to be tested under permanent coupling of ring and tail relaxation. This interpretation is supported by the *l*-PS_960_ control, whose molecular weight is comparable to the tail segment of *t*-PS_1633_. Unlike *t*-PS_1633_, *l*-PS_960_ does not exhibit a comparable plateau extension (Fig. S17), indicating that the extended plateau cannot be attributed solely to the linear tail, but is closely associated with the participation of the cyclic head in topological constraints. Furthermore, the molecular weight dependence of this topology-mediated relaxation is supported by the lower-molecular-weight tadpole polymer *t*-PS_538_, which does not exhibit a comparable plateau extension (Fig. S18), suggesting that sufficiently high molar mass is necessary to access the threading-dominated relaxation regime.

Notably, the tadpole sample may contain a small fraction of residual uncyclized linear chains. Residual uncyclized linear chains may contribute to the response. However, because approximately half of each tadpole polymer is the linear tail and the high-frequency response is dominated by linear segments,^20^ residual linear polymer alone is unlikely to account for the additional decade-wide plateau extension.

To obtain complementary structural visualization of the cyclization products, we employed advanced imaging technologies, including scanning tunneling microscopy (STM), atomic force microscopy (AFM), and transmission electron microscopy (TEM) to visualize representative tadpole polymers (*t*-PS_1633_ with original contour and *t*-PPA_1587_-g-PS with thickened contour). The polydispersity and aggregation tendencies of polymers present significant obstacles to their precise characterization. Ultra-high vacuum low-temperature scanning tunneling microscopy (STM) offers high-resolution imaging capabilities, particularly when combined with vacuum electrospray deposition (ESD).^38,39^ This ESD-STM approach has so far been mainly confined to conjugated and relatively rigid polymers. Its application to flexible polymers remains challenging, mainly due to the ionization efficiency and aggregation of such macromolecules during electrospray processing. Notably, the spatial arrangement of benzene rings in polystyrene enables visualization of these rings as bright spots *via* solution drop-casting deposition for STM imaging. In this study, a solution of *t*-PS_1633_ (1 × 10^−7^ mol L^−1^ in DCM) was introduced onto a Ag(111) substrate *via* drop-casting under a nitrogen atmosphere. Subsequent STM imaging under ultrahigh-vacuum and low-temperature conditions yielded representative images of *t*-PS_1633_ chains. As shown in Fig. 4a–d, some polymers aggregated at the step edges of the Ag(111) surface, while others were individually dispersed on the terraces. A small number of isolated chains exhibited tadpole-like morphology with distinct head–tail segmentation. One representative molecule displayed a total contour length of approximately 123 nm, consisting of a head segment around 80 nm and a tail segment about 43 nm in length (Fig. 4d). Owing to the inherent flexibility of the chains and their propensity to aggregate or entangle upon deposition, the incomplete feature in the upper-left corner of Fig. 4c most likely corresponds to a segment of an individual tadpole rather than an intact molecule. This observation underscores the persistent challenge of imaging ultrahigh-molar-mass polymers by STM. Given the limited number of fully isolated molecules and the potential conformational distortion of flexible chains during surface deposition, STM imaging is employed here solely as a complementary approach for representative structural visualization.

**Fig. 4:**
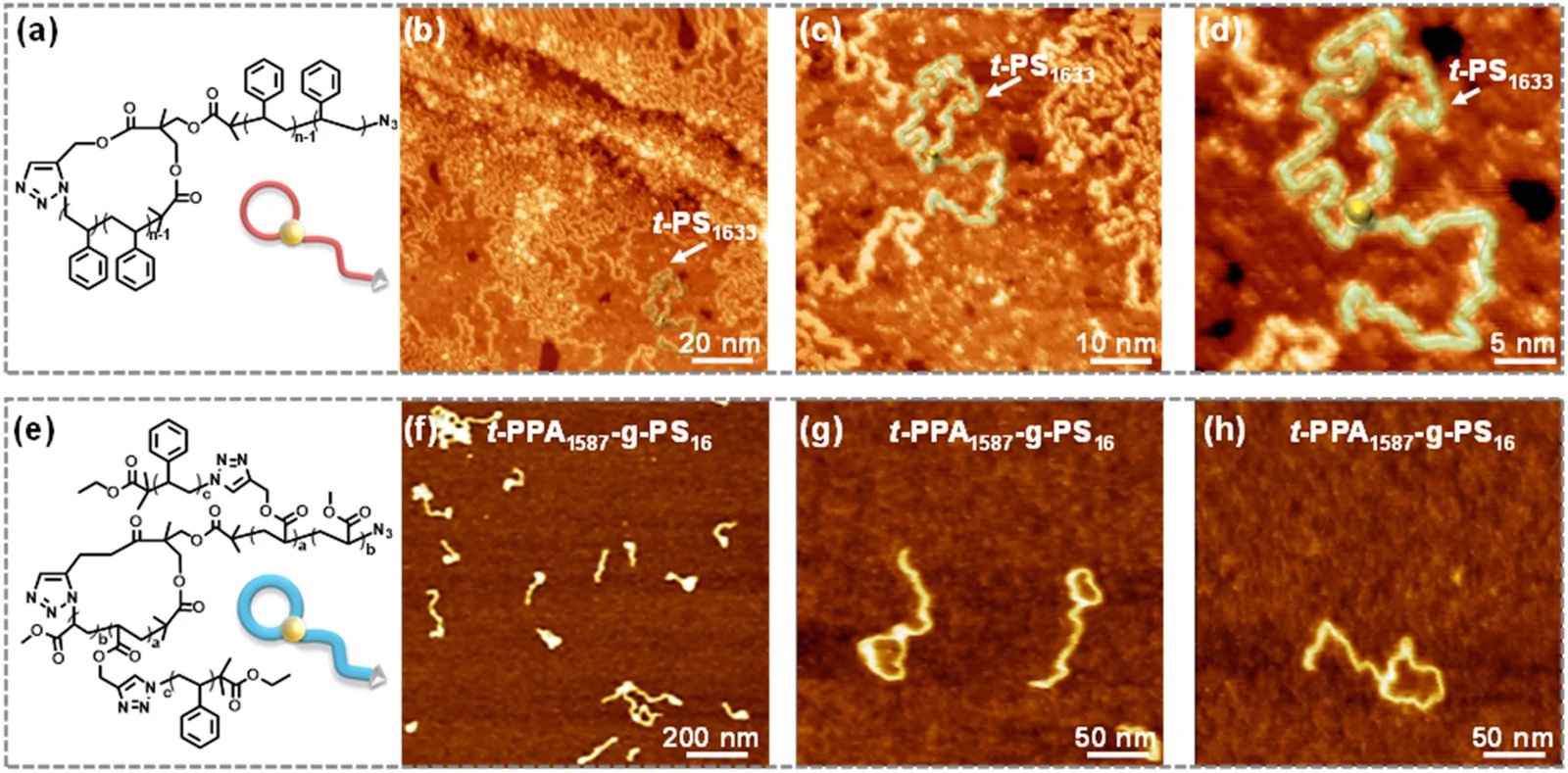
Single-molecule microscopy of original and contour-thickened tadpoles. (a–d) Chemical structure and scanning tunneling microscopy (STM) images of deposited *t*-PS_1633_ tadpole polymer chains on the surface of Ag(111) substrates. Scanning parameters: *U* = 3.0 V, *I* = 5 × 10^−12^ A; (e–h) chemical structure and atomic force microscope (AFM) phase images of tadpole polymer chains with thickened contour (*t*-PPA_1587_-graft-PS_16_) on the surface of silicon substrates.

Though STM enabled direct imaging of *t*-PS_*n*_, the low electronic contrast made it difficult to characterize *t*-PtBA_*n*_ accurately. As such, we synthesized a tadpole comb polymer (*t*-PPA_1587_-g-PS_16_) *via* the CuAAC click reaction grafting of PS_16_-N_3_ side chains for high-resolution AFM imaging. The grafting site was introduced by first converting the precursor *t*-PtBA_1587_ to tadpole poly(acrylic acid) (*t*-PAA_1587_), which was then functionalized into a poly(propargyl acrylate) (*t*-PPA_1587_) intermediate to install alkyne groups for the subsequent click reaction (Fig. S19).^40^ GPC confirms the topological stability of *t*-PAA_793_ after hydrolysis (Fig. S20). ^1^H NMR spectra show the characteristic signals from both the PAA-derived backbone and PS side chains, indicating the effective grafting of PS side chains onto the tadpole polymer (Fig. S21). Conventional and QD-GPC profiles further confirm the successful synthesis of PS-grafted tadpole polymers with higher molar mass (Fig. S22).

The grafting strategy significantly enhanced topographic contrast for AFM characterization. High-resolution AFM phase images clearly revealed the tadpole morphology with well-defined cyclic heads and linear tails (Fig. 4e–h). Although the irregularity in Fig. 4f is attributed to localized aggregation during spin-coating, most observed molecules exhibited a uniform width of ∼8 nm, comparable to approximately the twice sidechain contour length (∼4 nm) due to the tip size effect, consistent with the expected dimensions from the grafted side chains and indicating isolated, non-overlapping chains. Quantitative analysis of the contour lengths revealed a distribution (*e.g.*, ∼237.2 nm, ∼201.0 nm, and ∼322.5 nm). Specifically, 237.2 nm (head ∼117.9 nm and tail ∼119.3 nm), ∼201.0 nm (head ∼83.6 nm and tail ∼117.4 nm), and ∼322.5 nm (head ∼193.4 nm and tail ∼129.1 nm). The AB_2_ molecular design is expected to exhibit symmetry at the population level rather than for each individual molecule; therefore, measurements were collected from 38 individual tadpole-like molecules (Fig. S23a–c). The averaged head-to-tail contour-length ratio was approximately 1.16 : 1, close to the expected population average of 1 : 1. Furthermore, based on the morphological classification of tadpole-like and linear chains in AFM images, an estimated tadpole fraction of approximately 79.7% was obtained after excluding ambiguous structures (Fig. S23d–f). It should be noted that these AFM estimates are subject to chain flexibility, surface-deposition effects, and tip convolution, and thus should not be interpreted as solution-state contour lengths or as a definitive bulk-purity measurement. Analogous to AFM images, TEM images also provided corroborative support for the tadpole topology (Fig. S24). Therefore, the well-defined tadpole topology captured by advanced imaging technologies further supports the successful synthesis of various tadpole polymers using our developed dual-terminal intramolecular cyclization strategy.

Nevertheless, it must be acknowledged that the cyclization products described above are not exclusively tadpole polymers. Although GPC analysis under dilute conditions (*C*_i_ ≤ 18 g L^−1^) shows no detectable dimer peaks (Fig. S11–S12), residual linear precursors may remain. Liquid chromatography at critical conditions (LCCC) can in principle separate linear from tadpole topologies. Fig. S25a–c presents LCCC characterization of linear polystyrene (*l*-PS_538_) and tadpole-shaped polystyrene (*t*-PS_538_) in a tetrahydrofuran (THF)/acetonitrile (ACN) mixed solvent system. As the THF volume fraction decreases from 70% to 50%, the retention behaviors of the two species progressively diverge, indicating a transition from size-exclusion to adsorption-dominated separation. At *φ*_THF_ = 50%, the linear PS reaches the critical adsorption point (CAP) (Fig. S25c), where polymer–stationary phase interactions are balanced by entropic penalties. Under this condition, the retention volume (*V*_e_ = 19.47 mL) becomes independent of molecular weight, yielding a sharp and symmetric peak. In contrast, the tadpole-shaped polymer shows significantly delayed elution under identical conditions (Fig. S25f). The cyclic head restricts chain conformational freedom, while the linear tail enables multiple segmental contacts with the stationary phase, resulting in enhanced effective adsorption. This multivalent interaction disrupts the critical balance observed for linear chains, leading to a substantially increased retention volume (*V*_e_ = 29.48 mL) and clear separation from the linear precursor. Peak-area analysis gives an estimated tadpole fraction of 92.8% for *t*-PS_538_, supporting the usefulness of LCCC for topology-based analytical separation within this molar-mass range.

However, LCCC has important limitations. Its low loading capacity restricts it to analytical-scale assessment, and its linear-cyclic separation capability is limited, failing to resolve linear chain contaminants present in low abundance, making it impractical to obtain the large quantities required for rheological or microscopic studies. Another important limitation is that its reliability decreases markedly above ∼170 kDa because the limited pore size of the C18 columns reduces the separation efficiency for ultrahigh-molar-mass species. During attempted analysis of *t*-PS_1633,_ increased column backpressure and a substantially reduced UV response under identical injection conditions indicated sample adsorption. Therefore, reliable LCCC purity estimates are not available for *t*-PS_1633_ or *t*-PS_3766_. For the PtBA series, standardized LCCC separation protocols remain unreported, which need further exploration in future work.^41^ AFM morphology-based classification provided a complementary estimate of approximately 79.7% tadpole structures for *t*-PtBA_1587_ after excluding ambiguous molecules (Fig. S23), although this estimate is inherently influenced by surface-deposition effects. Developing scalable purification methods together with reliable purity-assessment techniques for ultrahigh-molar-mass topological polymers therefore remains an important challenge.

To demonstrate the versatility of our tadpole polymers as a functional platform, we established post-polymerization modification protocols for both PS and PtBA backbones (Fig. 5a and b). High-conversion transformations were achieved *via* Au-catalyzed bromination of *t*-PS_1633_ (>94% DS, degree of substitution, Table S11) and TFA-catalyzed hydrolysis of *t*-PtBA_1587_ (>99% DS, Tables S12–S13),^42^ yielding key intermediates *t*-PS_1633_-Br and *t*-PAA_1587_. The chemical composition of *t*-PS_1633_-Br was supported by ^1^H NMR and FTIR, while GPC showed no major high-molar-mass shoulder (Fig. S26a–c). The brominated intermediate served as a versatile handle for Pd-catalyzed C–C coupling, and diverse functional groups including alkenes (20%), alkynes (14−29%), aromatics (10−50%), ethers (44−93%), and cyano (4.5%) were incorporated through Pd-catalyzed coupling (Fig. 5c and S27–S37). Concurrently, during the synthesis of tadpole-shaped graft polymers, *t*-PtBA_1587_ was hydrolyzed *via* TFA-catalyzed hydrolysis to afford *t*-PAA_1587_ with 99% degree of substitution. The PAA intermediate underwent TMG-catalyzed esterification to introduce alkyl, fluoroalkyl, ionic, and PEG side chains with high efficiency (DS from 50% to 99%) quantified by ^1^H NMR (Fig. S38–S46 and Tables S12–S13). This modular modification strategy effectively expands the well-defined tadpole architecture into a vast functional library, paving the way for investigating the interplay between specific chemical functionalities and topological constraints in determining material properties (Fig. S47). It should be noted that the broad range of substitution levels observed for the Pd-catalyzed product series (4.5–99%) primarily reflects differences in the intrinsic reactivity of the coupling partners together with variations in the reaction conditions employed, including substrate ratio, catalyst loading, and reaction temperature. Accordingly, the reported degrees of substitution are intended to demonstrate the versatility of the post-polymerization modification platform rather than the maximum attainable conversion for each transformation.^43^

**Fig. 5:**
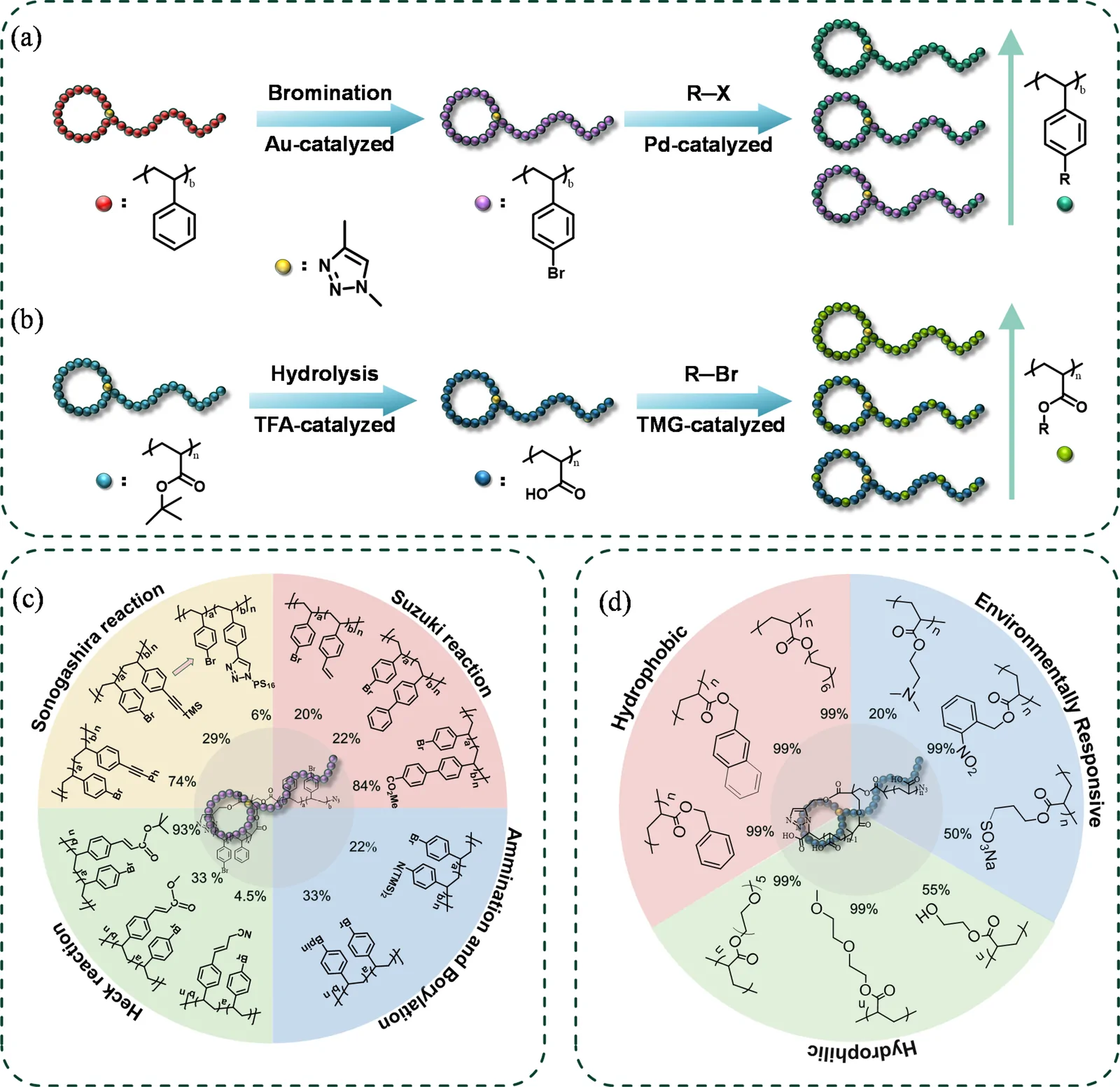
Diversifying tadpole polymer libraries *via* quantitative post-polymerization modifications. Schematic illustration of facile and universal modification routes for (a) *t*-PS_*n*_ and (b) *t*-PtBA_*n*_ backbones *via* Pd-catalyzed coupling and TMG-catalyzed esterification. (c and d) Established libraries of functional tadpole polymers demonstrating tailored incorporation of alkenes, alkynes, aromatics, cyano, alkyl, fluoroalkyl, ionic, and PEG functionalities.

Regarding the potential influence of post-polymerization modification on the integrity of the tadpole topology, the triazole linkage formed through the CuAAC reaction is generally considered chemically stable and is expected to remain intact under the Pd-catalyzed coupling conditions.^43^ Furthermore, because Pd-catalyzed post-polymerization functionalization not only alters the chemical composition of the polymer but also results in different degrees of functionalization between linear precursors and tadpole polymers, the observed GPC elution shifts can be affected by changes in molar mass, side-chain incorporation, and topology simultaneously. Therefore, GPC shifts alone cannot unambiguously verify the preservation of the tadpole topology.

## Conclusion

In summary, we have established an efficient synthetic platform with improved scalability relative to traditional intramolecular cyclization for symmetric tadpole polymers with ultrahigh molecular masses (0.4–1.0 MDa) and well-defined head-to-tail geometry—the key topological intermediate bridging linear and cyclic architectures. The platform provides structurally precise, ultra-long tadpole chains in quantities sufficient for systematic physical characterization. Using these samples, comparative rheological measurements show that only ultrahigh-molecular-weight tadpoles exhibit a markedly extended entanglement plateau (1–2 orders of magnitude beyond linear precursors), whereas their lower-molar-mass counterparts do not, indicating that a sufficiently high molecular weight is required to access threading-dominated dynamics. Although the isolated samples may contain a minor amount of residual linear precursor, the overall rheological trends consistently support topology-mediated relaxation in the ultrahigh-molecular-weight regime. Single-molecule visualization and modular post-polymerization functionalization further demonstrate the versatility of this platform. By making this topological intermediate experimentally accessible, this work provides a materials platform for systematic studies of topology–property relationships across chain dynamics, rheology, and soft materials design, moving beyond the linear–cyclic binary paradigm in future.

## Author contributions

All authors participated in the conceptualization, experimental design, data analysis, and manuscript preparation. The synthesis and characterization were carried out by W. L., J. G., Y. D., W. H., Y. W., and M. Z. The project was supervised by X. G., Z. C., L. L., and W. Z., Z.-C. Y., X. L., and Y. S. provided theoretical support and contributed to the interpretation of results. All authors reviewed and approved the final manuscript.

## Conflicts of interest

The authors declare no competing interest.

## Supplementary Material

SC-017-D6SC04605C-s001

## Data Availability

The datasets supporting this article have been uploaded as part of the supplementary information (SI). Supplementary information is available. See DOI: https://doi.org/10.1039/d6sc04605c.
