# Supplementary material for: Ultrahigh-molecular-weight tadpole polymers with statistically symmetric head-to-tail geometry: the key topological intermediate bridging linear and cyclic architectures
Source: Chem Sci. 2026 Aug 11;17(39):18701–10. doi: 10.1039/d6sc04605c (PMC13482460; doi:10.1039/d6sc04605c)
Supplement: Supplementary file 1 [file SC-017-D6SC04605C-s001.pdf]

Supplementary Information (SI) for Chemical Science.
This journal is © The Royal Society of Chemistry 2026

Supporting Information

Ultrahigh-Molecular-Weight Tadpole Polymers with Statistically Symmetric Head-to-Tail Geometry: The Key Topological Intermediate Bridging Linear and Cyclic Architectures

Wendi Liang[a],#, Jun Gong[a],#, Yongkang Deng[b], Wenjie He[a], Yiren Wang[a], Mo Zhu[a], Xin Guan[a]*, Zhi-Chao Yan[c], Zhi Chen[a]*, Xiaopeng Li[a],[d], Yu Song[b], Lianwei Li[a]*, Wenke Zhang[b]

[a] College of Chemistry and Environmental Engineering, Shenzhen University, Shenzhen 518060, China

[b] State Key Laboratory of Supramolecular Structure and Materials, College of Chemistry, Jilin University, Changchun, 130012, China

[c] School of Chemical Engineering and Light Industry, Guangdong University of Technology, Guangzhou 510006, China

[d] State Key Laboratory of Organometallic Chemistry, Shanghai Institute of Organic Chemistry, University of Chinese Academy of Sciences, Shanghai 200032, China

Email: xinguan@szu.edu.cn; zhi.chen@szu.edu.cn; lianweili@szu.edu.cn

Author Contributions: W.L. and J.G. contribute equally to this work.

Table of Contents

1) Materials..7
2) Characterization..8
^{1}H Nuclear Magnetic Resonance (^{1}H NMR) 8
Fourier Transform Infrared Spectroscopy (FTIR)..8
Matrix-Assisted Laser Desorption/Ionization Time-of-Flight Mass Spectrometry (MALDI-ToF-MS).. 9
Dynamic Light Scattering (DLS) 10
Gel Permeation Chromatography (GPC) 10
Rheological measurements. 11
Ultrahigh-Vacuum Cryogenic Scanning Tunneling Microscopy (UHV-STM). 11
Atomic Force Microscopy (AFM)...11
Transmission Electron Microscope (TEM). 12
Liquid Chromatography at the Critical Condition (LCCC)..12
3) Experimental procedures for the synthesis of tadpole polymers. 13
3-1. Preparation of bromo-terminated polystyrene (*l*-PS_n-2Br) linear precursors. 13
3-2. Preparation of azide-terminated polystyrene (*l*-PS_n-$2N_3$) macromonomers........................13
3-3. Preparation of tadpole-shaped polystyrene (*t*-PS_n)...14
3-4. Preparation of tadpole poly(tert-butyl acrylate). 15
3-5. Chain length and concentration effects on the synthesis of tadpole polystyrene. 15
4) Preparation of tadpole-bottlebrush polymers from *t*-PtBA 16
4-1. Conversion of tadpole PtBA into poly(acrylic acid) (PAA)...16
4-2. Synthesis of the poly(propargyl acrylate) (PPA) backbone 17
4-3. Synthesis of benzyl azide (BA) 17
4-4. Synthesis of tadpole-bottlebrush polymers *t*-PPA_{1587}-g-PS_{16}...18
5) Modifications of tadpole polymer 19
5-1. Bromination of tadpole polystyrene 19
5-2. Derivatizations of brominated polystyrene...20
5-2-1. General procedure for sonogashira coupling 20
5-2-2. General procedure for suzuki-miyaura coupling 23

5-2-3. General procedure for heck coupling 26

5-2-4. Miyaura borylation with $B_2(pin)_2$ 28

5-2-5. Ammination with LiHMDS 29

5-3. General procedure for TMG-Catalyzed side-chain grafting of tadpole poly(acrylic acid) ..30

5-4. A general blueprint for functional tadpole polymers via backbone-specific cyclization 31

6) Supporting Tables 33

Table S1. Experiment conditions for *l*-PS_n 33

Table S2. Experiment conditions for *l*-$PtBA_n$ 33

Table S3. Summary of synthetic scale, isolated yield, and batch-to-batch reproducibility of representative ultrahigh-molecular-weight tadpole polymers 33

Table S4. Molecular parameters of *l*-PS_n and *t*-PS_n samples from conventional GPC and DLS 34

Table S5. Molecular parameters of *l*-PS_n and *t*-PS_n obtained from QD-GPC 34

Table S6. Molecular parameters of *l*-$PtBA_n$ and *t*-$PtBA_n$ samples from conventional GPC and DLS 35

Table S7. Molecular parameters of *l*-$PtBA_n$ and *t*-$PtBA_n$ obtained from QD-GPC 35

Table S8. Peak retention volumes for *l*-PS_{3766} ($V_{e, l}$) and *t*-PS_{3766} ($V_{e, t}$) from five replicate SEC injections and the corresponding peak shift (ΔV_e)....36

Table S9. Experimental parameters for molecular chain length and concentration gradient in the ring-closing reaction of tadpole polymers 36

Table S10. Absolute molecular weight of unimer and multimer peaks for linear precursors (*l*-PS_n) and tadpole polymers (*t*-PS_n) under high-concentration conditions (C_i = 162 g/L)....37

Table S11. Experimental conditions for polystyrene bromination 37

Table S12. Functionalization reagents for poly(acrylic acid) 38

Table S13. 1H NMR characterization for grafted polymers D1-D9 38

7) Supporting Figures 40

Figure S1. MALDI-TOF MS of the *l*-PS_{56} sample (a) with two bromide ends and (b) with two azide ends 40

Figure S2. MALDI-TOF MS of the *l*-$PtBA_{63}$ sample (a) with two bromide ends and (b) with two azide ends 40

Figure S3. (a) ^{1}H NMR spectrum of *t*-PS_{56} in deuterated chloroform. (b) MALDI-TOF MS of *t*-PS_{56} sample 40

Figure S4. (a) ^{1}H NMR spectrum of *t*-$PtBA_{63}$ in deuterated chloroform. (b) MALDI-TOF MS of *t*-$PtBA_{63}$ 41

Figure S5. (a-d) FTIR spectra of *l*-PS_n-2Br, *l*-PS_n-$2N_3$ and *t*-PS_n ($n \geq 218$) samples..................41

Figure S6. (a-c) FTIR spectra of *l*-$PtBA_n$-2Br, *l*-$PtBA_n$-$2N_3$ and *t*-$PtBA_n$ ($n \geq 214$) samples.....41

Figure S7. Conventional traces for the remaining l-PtBAn and their corresponding tadpole polymers (*t*-$PtBA_n$) using THF as the eluent, where traces (a), (b), and (c) correspond to polymers with a degree of polymerization (n) of 593, 1587, and 5283, respectively 42

Figure S8. (a-f) Individual presentation of GPC elution curves from repeated cyclization r eaction tests for *l*-PS_{3766} and *t*-PS_{3766} 42

Figure S9. QD-GPC traces comparing the differential refractive index (RI), ultraviolet (UV), low-angle light scattering (LALS), and viscosity (DP) signals for *l*-PS_n and *t*-PS_n, with degrees of polymerization (n) of (a) 538 and (b) 1633, respectively 43

Figure S10. QD-GPC traces comparing differential refractive index (RI), ultraviolet (UV), low an gle light scattering (LALS), and viscosity (DP) signals for *l*-$PtBA_n$ and *t*-$PtBA_n$, with degrees o f polymerization of (a) 214 and (b) 593, (c) 1587, (d) 3174 and (e) 5283 respectively 43

Figure S11. Conventional GPC curves of *t*-PS_n obtained from *l*-PS_n samples with different molecular mass under varying injection and solution concentrations (a) n = 56, (b) n = 218; (c) n = 538; (d) n = 1633 and (e) n = 3766. The injection concentration gradient is C_i = 2, 6, 18, 54 ,162, 324 g/L, and the solution concentration gradient is C_s = 0.18, 0.54, 1.64, 4.91, 14.71,29.4 5 g/L. 44

Figure S12. (a) Dependence of the peak molecular mass ratio between tadpole-shaped polymers and linear precursors ($M_{rel, p, t}/M_{rel, p, l}$) on injection concentration (C_i) for samples with varyi ng molecular weights. (b) Correlation between the unimer fraction (quantified via GPC pe ak deconvolution) of linear precursors (*l*-PS_n) and tadpole polymers (*t*-PS_n) and C_i. 45

Figure S13. QD-GPC for the *l*-PS_n and *t*-PS_n with (a) n = 218, (b) n = 538, (c) n = 1633, and (d)n = 3766, under C_i = 162 g/L solution conditions, illustrating the four signals—different ial refractive index, ultraviolet, light scattering, and viscosity. 45

Figure S14. Intrinsic viscosity ([η]) vs. elution volume for *l*-PS_n and *t*-PS_n, with degrees of polymerization of (a) 218 and (b) 538, and (c) 3766, respectively. Measured by QD-GPC. 46

Figure S15. Intrinsic viscosity ([η]) vs. elution volume for *l*-$PtBA_n$ and *t*-$PtBA_n$, with degree s of polymerization of (a) n = 214, (b) n = 593, (c) n = 793, (d) n = 3174, (e) n = 5283, and (f) n = 7936, respectively. Measured by QD-GPC...46

Figure S16. Analysis of the radius of gyration versus absolute molecular mass at the peak positions in QD-GPC for (a) *l*-PS_n/*t*-PS_n and (b) *l*-$PtBA_n$/*t*-$PtBA_n$. The data represent po lymers with degrees of polymerization of 1633, 3766 for PS, and 1587, 3174, 5283, 7936 for PtBA. Samples with insufficient molecular dimensions ($n \leq 538$ for PS; $n \leq 793$ for PtB A) were excluded due to poor signal-to-noise ratio. 46

Figure S17. Frequency dependence of the storage (G′) and loss (G″) moduli for the linear *l*-PS_{960} control. Frequency sweeps collected at 70-130 °C were horizontally shifted to construct a time–temperature superposition (TTS) master curve 47

Figure S18. Frequency dependence of the storage (G′) and loss (G″) moduli for *l*-PS_{538} and *t*-PS_{538}. Frequency sweeps collected at 70-130 °C were horizontally shifted to construct time–temperature superposition (TTS) master curves 47

Figure S19. Synthesis route of *t*-PPA_{1587}-g-PS_{16} 48

Figure S20. (a) Synthetic route from Poly(tert-butyl acrylate) to Poly(methyl acrylate). (b) Conventional curves for the remaining *l*-$PtBA_{793}$ and their corresponding tadpole polymers using THF as the eluent. (c) Conventional curves for the remaining *l*-PMA_{793} and their corresponding tadpole using THF as the eluent 48

Figure S21. ¹H NMR spectra of (a) *t*-PAA_{1587} in DMSO-d_6, (b) *t*-PPA_{1587} in $CDCl_3$, (c) benzyl azi de in $CDCl_3$, and (d) *t*-PPA_{1587}-g-PS_{16} in $CDCl_3$ 49

Figure S22. (a) Conventional GPC curves and (b) QD-GPC traces comparing of differential refractive index (RI), ultraviolet (UV), low angle light scattering (LALS), and viscosity (DP) signals for the *t*-PPA_{1587} and *t*-PPA_{1587}-g-PS_{16} 49

Figure S23. AFM-based statistical analysis of the head-to-tail geometry and morphology-based cyclization efficiency of *t*-$PtBA_{1587}$. Red circles indicate tadpole-like molecules, blue circles indicate linear precursors, and lime-green circles indicate ambiguous structures excluded from the statistical analysis 50

Figure S24. TEM images of *t*-PPA_{1587}-graft-PS_{16} deposited on a carbon-coated copper grid..... 50

Figure S25. Critical adsorption behavior of *l*-PS_{538} (a–c) and *t*-PS_{538} with triazole-ring (d–f) in LCCC: evolution of chromatograms with decreasing φ_{THF} 51

Figure S26. (a) ¹H NMR spectra, (b) GPC, and (c) FTIR spectra of *t*-PS_{1633} and *t*-PS_{1633}-$Br_{0.94}$ 51

Figure S27. 1H NMR (500 MHz, $CDCl_3$) spectrum of P1 52

Figure S28. 1H NMR (500 MHz, $CDCl_3$) spectrum of P2 52

Figure S29. 1H NMR (500 MHz, $CDCl_3$) spectrum of P3 53

Figure S30. 1H NMR (500 MHz, $CDCl_3$) spectrum of P4 53

Figure S31. 1H NMR (500 MHz, $CDCl_3$) spectrum of P5 54

Figure S32. 1H NMR (500 MHz, $CDCl_3$) spectrum of P6 54

Figure S33. 1H NMR (500 MHz, $CDCl_3$) spectrum of P7 55

Figure S34. 1H NMR (500 MHz, $CDCl_3$) spectrum of P8 55

Figure S35. 1H NMR (500 MHz, $CDCl_3$) spectrum of P9. 56

Figure S36. 1H NMR (500 MHz, $CDCl_3$) spectrum of P10. 56

Figure S37. 1H NMR (500 MHz, $CDCl_3$) spectrum of P11. 57

Figure S38. 1H NMR (500 MHz, $CDCl_3$) spectrum of D1. 57

Figure S39. 1H NMR (500 MHz, $CDCl_3$) spectrum of D2. 58

Figure S40. 1H NMR (500 MHz, $CDCl_3$) spectrum of D3. 58

Figure S41. 1H NMR (500 MHz, , DMSO-d6) spectrum of D4. 59

Figure S42. 1H NMR (500 MHz, $CDCl_3$) spectrum of D5. 59

Figure S43. 1H NMR (500 MHz, D2O) spectrum of D6. 60

Figure S44. 1H NMR (500 MHz, DMSO-d6) spectrum of D7. 60

Figure S45. 1H NMR (500 MHz, $CDCl_3$) spectrum of D8. 61

Figure S46. 1H NMR (500 MHz, $CDCl_3$) spectrum of D9. 61

Figure S47. Preparation of potential tadpole polymers library based on dual-terminal intramolecular cyclization strategy. 62

References 63

1) Materials.

All chemicals were used as received unless otherwise specified. Styrene (> 99%), phenylacetylene (> 99%), *tert*-butyl acrylate (> 99%), methyl acrylate (> 99%), were purchased form Aladdin and passed through a basic alumina column to remove inhibitors, distilled under vacuum, and stored at -20 °C. *N,N*-dimethylformamide (DMF, Sinopharm, AR) was dried over anhydrous $MgSO_4$ and purified by reduced-pressure distillation. Dichloromethane (DCM, Sinopharm, AR), tetrahydrofuran (THF, Aladdin, AR), acetonitrile (CAN, Aladdin, AR), acetone (Sinopharm), toluene (Sinopharm), $CHCl_3$ (Sinopharm) and anisole (Aladdin, AR), 1,4-dioxane (Aladdin, AR), 1,2-dichloroethane (DCE, Adamas), were distilled over CaH_2 prior to use. Copper(I) bromide (CuBr, Alfa, 98%) was washed with glacial acetic acid to remove soluble impurities, filtered, rinsed with deoxygenated ethanol, dried under vacuum, and stored in glovebox. Propargyl 2,2-bis[(2-bromo-2-methylpropanoyloxy)methyl]propionate was synthesized according to literature procedures, with its structure confirmed by ^{1}H NMR[1]. Sodium azide (NaN_3, Aladdin, 99%), tin(II) 2-ethylhexanoate ($Sn(EH)_2$, Aladdin, 95%), *N,N,N',N'',N''*-pentamethyldiethylenetriamine (PMDETA, Aladdin, 99%), tris(2-dimethylaminoethyl)amine (Me_6TREN, Aladdin, 98%), and methanol (Sinopharm, AR), were used as received. N-bromosuccinimide (NBS, 99%, Energy Chemical) was recrystallized from hot water three times prior to use. Gold(III) chloride ($AuCl_3$, 99%, Sigma-Aldrich), tetrakis(triphenylphosphine)palladium ($Pd(PPh_3)_4$, 99%), [1,1'-

bis(diphenylphosphino)ferrocene]dichloropalladium(II) ($Pd(dppf)Cl_2$, 99.95%), bis(dibenzylideneacetone)- palladium ($Pd(dba)_2$, 99.95%), *bis*(triphenylphosphine) palladium(II) chloride ($Pd(PPh_3)_2Cl_2$, 99.95%) was purchased from Energy Chemical company. Tri-*tert*-butylphosphine ($P(t\text{-}Bu)_3$, 1.0 M in toluene), copper(I) iodide (CuI, ⩾99.95%), NaOAc (98.5%, TCI), Na_2CO_3 (99.8%), K_2CO_3 (99.8%), KOAc (99%), Diisopropylamine(DIPA), phenylboronic acid(99%), 4-(methoxycarbonyl)phenylboronic acid (99%), allyl cyanide (98%), bis(pinacolato)diboron ($B_2(pin)_2$, 98%), lithium bis(trimethylsilyl)amide (LiHMDS, 1.0 M in THF) were purchased from Aladdin. Potassium vinyltrifluoroborate (98%) and trimethylsilylacetylene (TMSA, 98%) were purchased from Adams Chemicals. 1,1,3,3-tetramethylguanidine (TMG, 99%), trifluoro-acetic acid (TFA, 98%), triethylamine(TEA, AR), diethyl ether (Sinopharm, AR), ethyl α-bromoisobutyrate (98%), benzyl bromide (97%), 2-bromoethanol (95%), 1-(bromomethyl)naphthalene (97%), sodium 3-bromopropane-1-sulfonate (98%), 2-(dimethylamino)ethyl bromide hydrobromide (97%), di(ethylene glycol) 2-bromoethyl methyl ether (95%), 1-bromo-2-(2-methoxyethoxy)ethane (95%), and 2-nitrobenzyl bromide (95%), 1-bromododecane (98%) and 1-bromooctadecane (98%), iodomethane (99%) were obtained from Aladdin and were used as received.

2) Characterization

^{1}H Nuclear Magnetic Resonance (^{1}H-NMR). ^{1}H NMR Spectra were recorded on a Bruker Avance III Ascend 400 spectrometer (400 MHz) using tetramethylsilane (TMS)

as an internal standard and $CDCl_3$ as the solvent.

Fourier Transform Infrared Spectroscopy (FTIR). Measurements were performed on a Bruker VECTOR-22 IR spectrometer. Samples were prepared as KBr pellets, with spectra acquired over 64 scans at a resolution of 4.0 cm^{-1}.

Matrix-Assisted Laser Desorption/Ionization Time-of-Flight Mass Spectrometry (MALDI-TOF-MS). Analysis was conducted on a Bruker UltrafleXtreme instrument. Samples were prepared using trans-2-[3-(4-tert-butylphenyl)-2-methyl-2-propenylidene]malononitrile (DCTB) as the matrix and sodium trifluoroacetate (NaTFA) as the cationizing agent. The matrix and NaTFA were mixed at a 10:1 (v/v) ratio, and data were collected in positive reflection mode. The theoretical m/z values for linear and tadpole-shaped polymers, including PS and PtBA were calculated using the following formula[2, 3]

$$M_1 = M_i + M_{st} \times DP - 2M_{HBr} + M_{Na^+}$$

$$M_2 = M_i + M_{st} \times DP - 2M_{Br} + 2M_{N_3} - 2M_{N_2} + M_{Na^+}$$

$$M_3 = M_i + M_{st} \times DP - 2M_{Br} + 2M_{N_3} - M_{N_2} + M_{Na^+}$$

$$M_4 = M_i + M_{tBA} \times DP + M_{Na^+}$$

$$M_5 = M_i + M_{tBA} \times DP - 2M_{Br} + 2M_{N_3} - 2M_{N_2} + M_{Na^+}$$

$$M_6 = M_i + M_{tBA} \times DP - 2M_{Br} + 2M_{N_3} + M_{Na^+}$$

M_1, M_2, M_3, M_4, M_5, and M_6 represent the theoretical molecular masses of the main peak ions for *l*-PS_n-2Br, *l*-PS_n-$2N_3$, *t*-PS_n, *l*-$PtBA_n$-2Br, *l*-$PtBA_n$-$2N_3$, and *t*-$PtBA_n$, respectively. M_{st} is the molecular weight of styrene (104.18 g/mol), M_{tBA} is the

molecular weight of *tert*-butyl acrylate (128.17 g/mol), M_{Br} is the mass of the bromine atom (79.90 g/mol), M_{N_3} is the molecular weight of the azido group (42.02 g/mol), M_{N_2} is the molecular weight of the nitrogen molecule (28.01 g/mol), and M_{Na^+} represents the molecular weight of the sodium ion (23 g/mol).

Dynamic Light Scattering (DLS). Hydrodynamic radius (R_h) distributions were derived from intensity-intensity time correlation functions $G^{(2)}(q,t)$ measured in self-beating mode. Laplace inversion and the Stokes-Einstein equation $R_h = (k_BT/6\pi\eta_0)/D$ were employed, where k_B, T, η_0, and D represent the Boltzmann constant, absolute temperature, solvent viscosity, and translational diffusion coefficient, respectively. Data analysis included cumulant analysis and the CONTIN algorithm. The linear polystyrene macromonomer samples were dissolved in toluene (poly(tert-butyl acrylate) were dissolved in THF), filtered through 0.25 μm membranes (Millipore) at 25 °C, and measured at a scattering angle of 15[3].

Gel Permeation Chromatography (GPC). Two GPC systems (Conventional GPC/QD-GPC) were utilized. Conventional GPC system is equipped with a P98II isocratic HPLC pump, T98 column oven (three Waters Styragel HR columns: HR6, HR4, HR3), and Shodex RI-201H refractive index detector. THF (HPLC grade) served as the eluent at 1.0 mL/min (35 °C). Calibration employed seven narrow-distribution polystyrene standards ($M_w = 2.05 \times 10^3 \sim 3.56 \times 10^6$ g/mol) to determine apparent M_n, M_w, M_p, and dispersity ($Đ = M_w/M_n$). Quadruple-detector absolute molecular weight system (QD-GPC) includes Waters 1515 isocratic HPLC pump, three Viscotek

columns (D6000M, D4000, D2000), Waters 717 PLUS autosampler, Waters 2414 RI detector, photodiode array detector (PDA, 190 ~ 900 nm), Wyatt mini-DAWN HELEOS-II multi-angle light scattering detector (7° and 90°), and Wyatt Visco Star viscometer. THF (HPLC grade) eluted at 1.0 mL/min. Sample concentrations (2 ~ 10 mg/mL) were filtered through 0.22 μm PTFE membranes prior to injection. Absolute M_p, radius of gyration (R_g), and intrinsic viscosity ([η]) were determined at 25 °C.

Rheological Measurements. Shear rheology frequency-sweep tests were conducted on an Anton Paar MCR 302e rheometer using an 8 mm parallel-plate geometry at temperatures from 70 to 130 °C and a strain of 0.3%. Data collected at different temperatures were horizontally shifted along the frequency axis to construct time–temperature superposition (TTS) master curves. The rheological curves shown in Figures 3e–f and S17–S18 are TTS master curves.

Ultrahigh-Vacuum Low-Temperature Scanning Tunneling Microscopy (UHV-LT-STM). Experiments were performed in a CreaTec instrument (Fischer & Co. GmbH) under an ultra-high vacuum (base pressure < 2.0×10^{-10} mbar). The Ag(111) substrate was cleaned through three cycles of Ar^+ sputtering (1.0 keV, 10 min) followed by annealing (500 °C, 10 min). Commercial Pt-Ir tips were employed for imaging. A 20 μL *t*-PS_{1633} solution (1×10^{-7} mol/L in DCM) was drop-casted onto Ag(111) under a N_2 atmosphere. STM imaging was carried out at -196 °C in a constant-current mode, with typical imaging parameters set to a bias voltage of 3.0 V and a tunneling current of 5×10^{-12} A. Data were processed using the WSxM software.

Atomic Force Microscopy (AFM). Samples for AFM analysis were dissolved in toluene and then introduced onto substrates by spin-casting. Practically, two drops of a dilute solution (0.001 g/L) were spin-cast on a 1 × 1 cm^2 silicon wafer at approximately 8000 rpm. All AFM images were recorded in air with a Cypher ES (Asylum Research, Santa Barbara, CA), operated in tapping mode. We used Silicon nitride cantilevers (SNL-10 from Bruker) with a nominal resonance frequency of 65 kHz, a nominal spring constant of 0.35 N/m and a nominal tip radius of 2 nm. Head and tail contour lengths were traced from AFM phase images in ImageJ. The head-to-tail ratio distribution was calculated from 38 individual tadpole-like molecules. For morphology-based cyclization estimates, clearly resolved tadpole and linear structures were classified separately, while overlapped, aggregated, or otherwise ambiguous structures were excluded from the population count.

Transmission Electron Microscope (TEM). The sample was dissolved in chloroform and subjected to overnight agitation using an orbital shaker to achieve a homogeneous solution. A 5 μL aliquot of this solution was pipetted and dropped onto a copper grid coated with an ultra-thin carbon film. After allowing the sample to adsorb onto the substrate for several minutes, any residual liquid was carefully wicked away from the edge of the grid using filter paper. The grid was subsequently air-dried in a petri dish or dried under an infrared lamp prior to TEM characterization. TEM imaging was performed on a JEOL JEM-2100F instrument operated at an acceleration voltage of 200 kV. The incident electron beam was adjusted to the lowest feasible intensity to

minimize radiation-induced damage to the sample. An objective aperture was inserted to enhance contrast under bright-field imaging conditions while further reducing electron beam exposure. All images were acquired with a consistent exposure time of 0.5 s to mitigate beam-induced degradation. All images were annotated with scale bars using the open-source tool Image J[4].

Liquid Chromatography at the Critical Condition (LCCC). The LCCC system consists an autosampler, P98I binary gradient pump, column group (two Waters C18 columns: 250 mm×4.6 mm, 100 Å pore， 5 um bead size；250 mm×4.6 mm, 300 Å pore， 5 um bead size) and UV-98II detector. The flow rate of mixed eluent (THF and ACN) was 0.3 mL/min and T = 35°C. The volume ratio of eluent was regulated to specific value by gradient pump. Before injection, all the sample solutions were filtrated by PTFE membranes (pore size: 0.45 um).

3) Experimental procedures for the synthesis of tadpole polymers[5]

3-1. Preparation of bromo-terminated polystyrene (*l*-PS_n-2Br) linear precursors

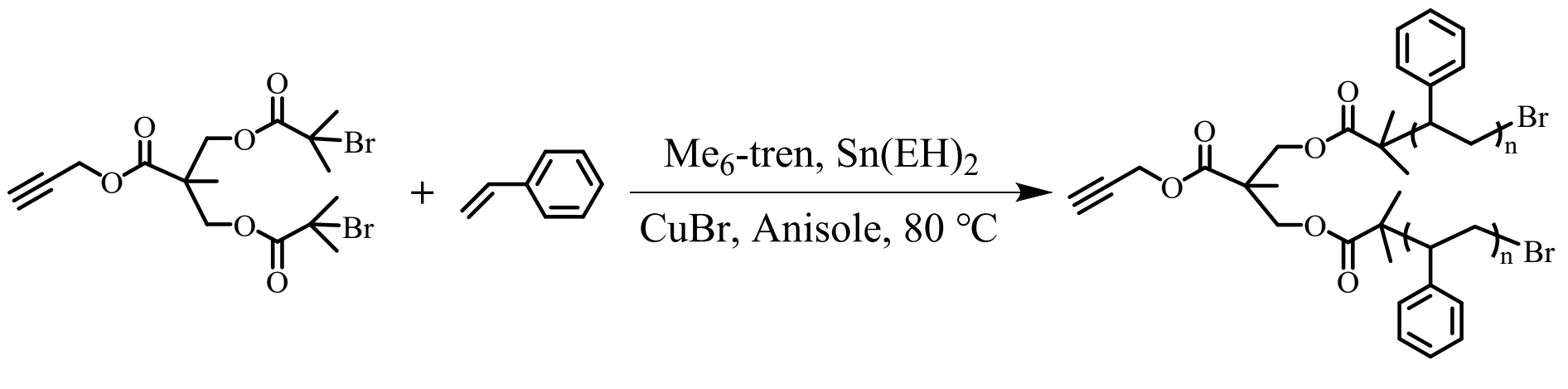

A dry glass tube charged with a stir bar was sequentially loaded with propargyl 2,2-*bis*[(2-bromo-2-methylpropanoyloxy)methyl]propionate (733.9 mg, 1.56 mmol), Me_6TREN (143.6 mg, 0.62 mmol), $Sn(EH)_2$ (252.9 mg, 0.62 mmol), styrene (St, 81.2 g, 0.78 mol), and anisole (60 mL). Oxygen was removed via three freeze-vacuum-thaw

cycles. CuBr (23.2 mg, 0.16 mmol) was added under frozen conditions, and the tube was flame-sealed. Polymerization proceeded at 80 °C for 3 h. The crude product was diluted with THF, passed through a neutral alumina column to remove metal salts, concentrated by rotary evaporation, precipitated into methanol/H_2O (1:1, v/v), and vacuum-dried at 40 °C (yield: 9.2 g, *l*-PS_{56}-2Br). *l*-PS_{218}-2Br, *l*-PS_{538}-2Br, *l*-PS_{1633}-2Br, and *l*-PS_{3766}-2Br were synthesized analogously (Detailed experimental conditions are provided in Table S1.).

3-2. Preparation of azide-terminated polystyrene (*l*-PS_n-$2N_3$) macromonomers

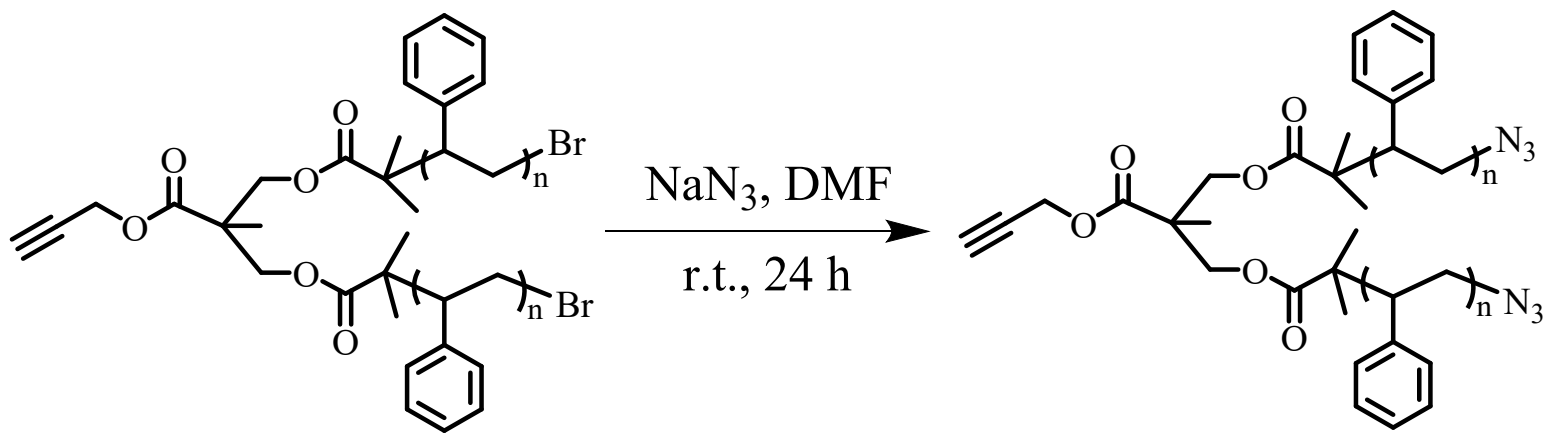

A mixture of *l*-PS_{56}-2Br (2.00 g, 0.32 mmol), DMF (10 mL), and NaN_3 (210 mg, 3.2 mmol) was stirred in the dark at room temperature for 24 h. DMF was removed by rotary evaporation (35 °C), and the residue was redissolved in THF, centrifuged to eliminate sodium salts, precipitated into cold methanol/H_2O (1:1, v/v), and dried under vacuum at 40 °C (yield: 1.80 g, *l*-PS_{56}-$2N_3$). Other *l*-PS_n-$2N_3$ derivatives were prepared similarly.

3-3. Preparation of tadpole-shaped polystyrene (*t*-PS_n)

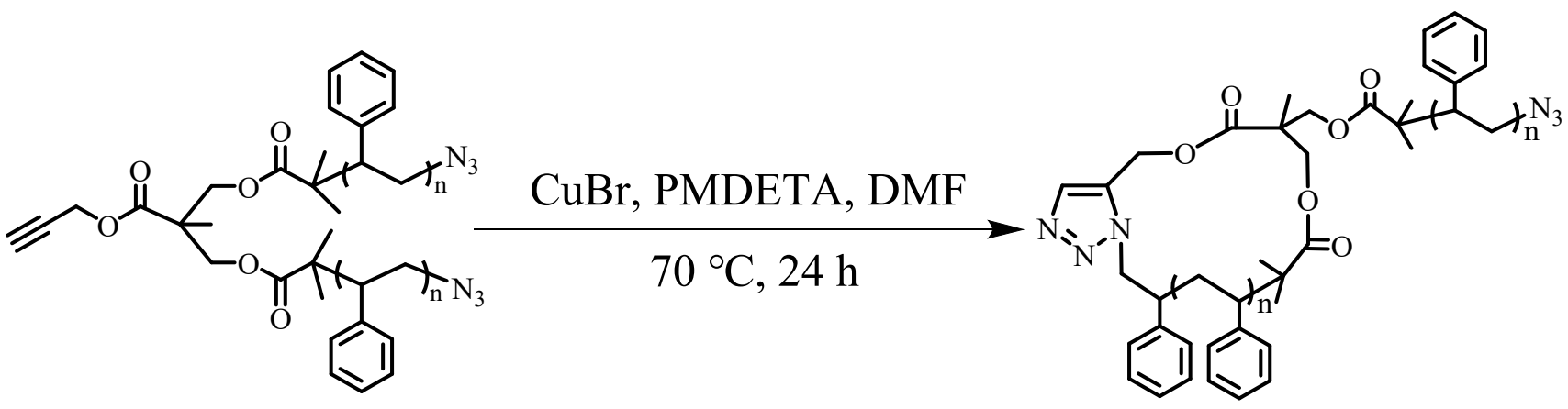

A 2 L flask charged with DMF (1.50 L) was degassed via Ar bubbling for 4 h. CuBr (1.45 g, 10.0 mmol) and PMDETA (2.64 g, 15.0 mmol) were added. A solution of *l*-PS_{56}-$2N_3$ (500 mg, 0.1 mmol) in degassed DMF (50.0 mL) was injected via syringe pump (0.5 mL/h, 70 °C). After completion, stirring continued for 24 h. DMF was removed by distillation, and the product was dissolved in THF, filtered through neutral alumina, concentrated, precipitated twice into methanol/H_2O (1:1, v/v), and dried under vacuum at 40°C (yield: 464 mg, *t*-PS_{56}). Other *t*-PS_n derivatives were synthesized analogously. For the representative high-molar-mass samples used in rheology, the same work-up comprised neutral-alumina filtration and repeated precipitation to remove copper catalyst and low-molar-mass species. No topology-selective preparative separation was performed, so a minor residual linear or coupled fraction may remain.

3-4. Preparation of tadpole poly(*tert*-butyl acrylate)

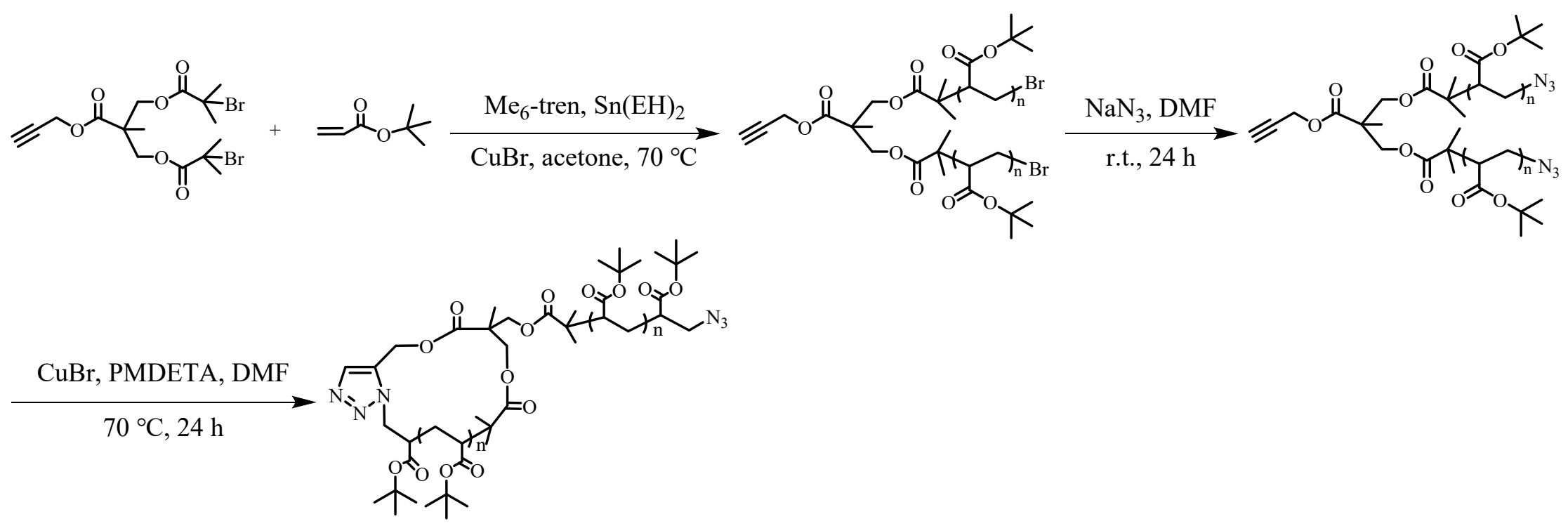

The synthesis of tadpole-shaped poly(*tert*-butyl acrylate) polymers (*t*-$PtBA_n$) was performed using the identical modular strategy as the PS system: (i) Synthesis of the linear precursor (*l*-$PtBA_n$-$2N_3$), detailed experimental conditions are provided in Table S2, (ii) copper-catalyzed azide-alkyne cycloaddition (CuAAC) click cyclization

reaction (for experimental procedures, refer to PS).

3-5. Chain length and concentration effects on the synthesis of tadpole polystyrene

Six 40 mL pressure-rated vials each containing anhydrous DMF (18 mL) were purged with Ar (30 min) via a dual-needle gas exchange system (refer to Figure 1b). PMDETA/CuBr solution (50 μL PMDETA + 20 mg CuBr in 2 mL DMF) was injected through gas-tight syringes, followed by additional Ar purging (30 min). Six precursor solutions of *l*-PS_n-$2N_3$ (2.5 mL, concentration gradient: 2, 6, 18, 54, 162, 324 g/L) were loaded into 2.5 mL SGE syringes mounted on a multi-channel pump. Injection parameters: syringe diameter = 7.4 mm, volume = 2 mL, flow rate = 0.5 mL/h (full conditions in Table S9).

4) Preparation of tadpole-bottlebrush polymers from *t*-PtBA[6]

4-1. Conversion of tadpole PtBA into poly(acrylic acid) (PAA)

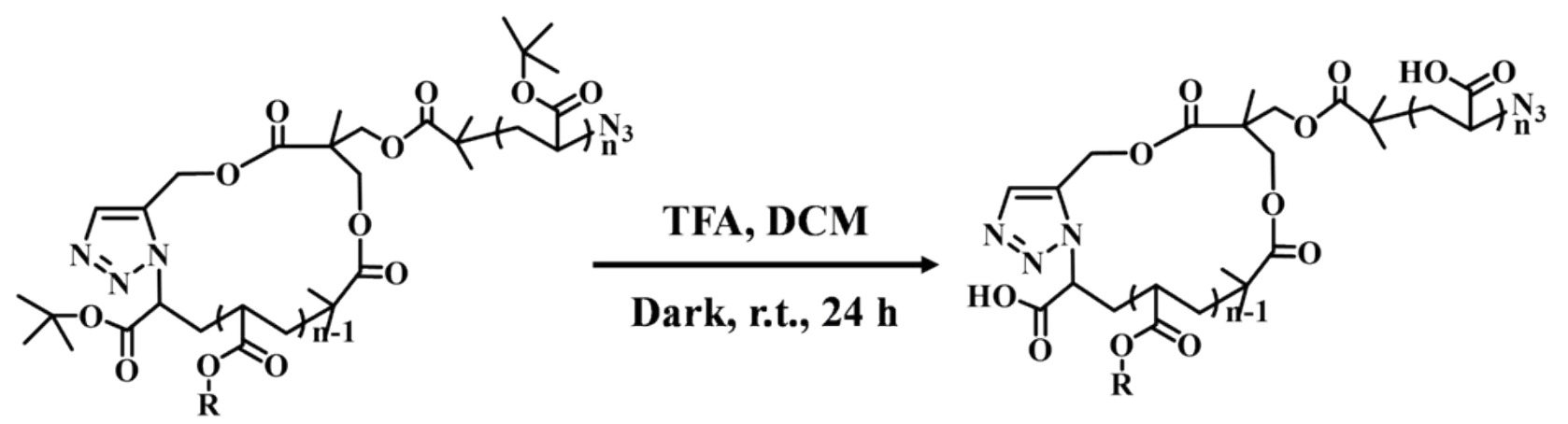

T-$PtBA_{1587}$ (6 g, 46.8 mmol with respect to the repeating unit) was dissolved in DCM (120 mL) and hydrolyzed for 24 h using TFA (26.7 g, 234 mmol) to produce PAA. At least two independent reactions were required to fully hydrolyze *tert*-butyl acrylate. After each hydrolysis, solvent was subsequently removed by filtration. The polymer was then washed thrice with diethyl ether and separated again. Upon the

completion of the third ether washing and filtration, the sample was dried under vacuum over night to remove any remaining volatiles. Once the polymer sample was completely dried after first hydrolysis cycle, we need to grind it into small particles to ensure adequate hydrolysis next time. Given the potential risk of initiator-core ester linkage cleavage during the TFA-mediated hydrolysis step, we evaluated the topological stability of *t*-PAA_{793} by comparing its hydrodynamic volume with that of its linear analog (*l*-PAA_{793}) using conventional GPC after converting both samples into the corresponding PMA derivatives (*l*-PMA_{793} and *t*-PMA_{793}) via methylation with iodomethane to prevent column adsorption (Figures S20a). Should the ester linkages remain intact, the tadpole topology would be preserved after hydrolysis and exhibit a smaller hydrodynamic volume than its linear counterpart; otherwise, both architectures would display similar hydrodynamic volumes. The GPC curve of *t*-PMA_{793} shifted significantly toward larger retention volumes (Figure S20b-c), confirming the integrity of the initiator ester linkages throughout the process and establishing the chemical stability of the tadpole topology.

4-2. Synthesis of the poly(propargyl acrylate) (PPA) backbone

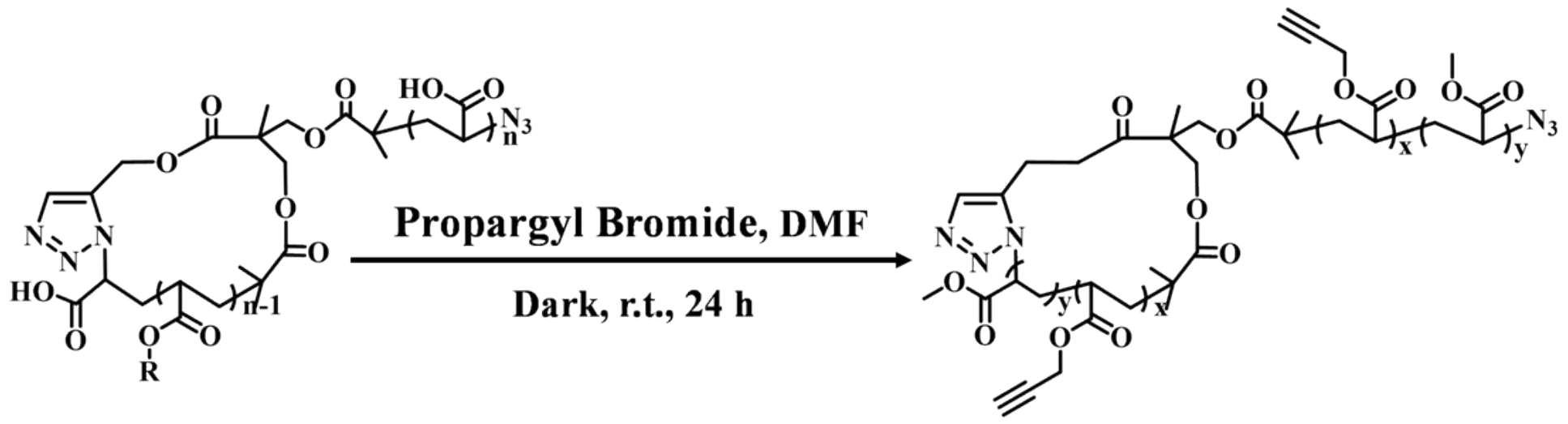

To a 5 mL vial equipped with a magnetic stir bar were successively added *N,N*-dimethylformamide (DMF, 1.5 mL), PAA (0.050 g, 0.7 mmol with respect to the

repeating unit) and propargyl bromide (0.165 g, 1.4 mmol) in a glove box. The reaction mixture was stirred at 45 °C for 8 h. Methyl iodide (CH_3I, 0.098 g, 0.7 mmol) was then added to cap remaining acids, and stirring continued at 45 °C for 2 hours. The reaction mixture was quenched by adding acetic acid (0.042 g, 0.7 mmol) to neutralize excess base/TMG. The polymer was purified by dialysis against an appropriate solvent, followed by solvent removal via rotary evaporation. The resulting PPA (0.03 g obtained) was dissolved in purified anhydrous DMF to make a stock solution. This stock solution was degassed by purging with nitrogen or argon and stored in a sealed vial inside the glovebox freezer at -19 °C for future use. The ^{1}H NMR spectrum of the product is shown in Figure S21b.

4-3. Synthesis of benzyl azide (BA)

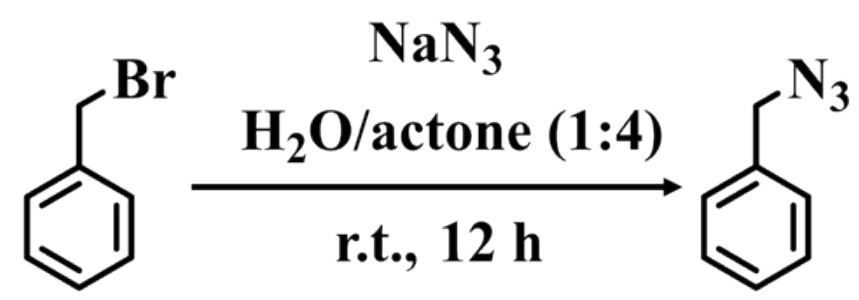

Benzyl bromide (0.300 g, 1.75 mmol) was dissolved in a mixture of water and acetone (10 mL/40 mL), followed by the addition of NaN_3 (0.244 g, 3.75 mmol). The reaction was stirred at room temperature for 12 h. Upon completion, the mixture was extracted with dichloromethane (50 mL). The organic layer was collected, and the aqueous phase was further extracted with DCM (3 × 10 mL). The combined organic extracts were dried over anhydrous Na_2SO_4, filtered, and concentrated under reduced pressure to afford benzyl azide as a yellow oil (0.340 g). The product was dissolved in DMF to prepare a standard stock solution, which was degassed with N_2, transferred to

a glovebox, and stored at –19 °C for further use. The ^{1}H NMR spectrum of the product is shown in Figure S21c.

4-4. Synthesis of tadpole-bottlebrush polymers *t*-PPA$_{1587}$-g-PS$_{16}$

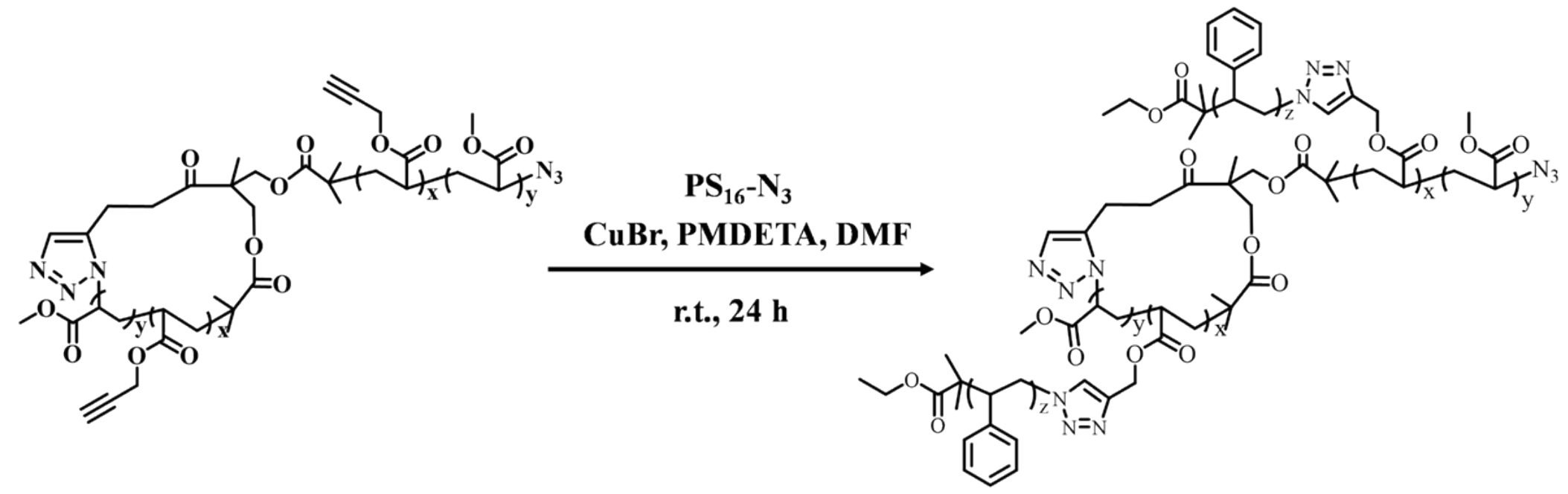

In a 5 mL vial equipped with a magnetic stir bar, PS$_{16}$-N$_3$ (100 mg, 0.06 mmol), CuBr (1.5 mg, 0.01 mmol), and DMF (1 mL) were added successively and stirred until complete dissolution. Then, Sn(EH)$_2$ (16 mg, 0.04 mmol) and PMDETA (3.5 mg, 0.02 mmol) were introduced, followed by the addition of PPA (2.8 mg, 0.018 mmol) to initiate the reaction. The mixture was stirred at 25 °C for 48 h. Subsequently, benzyl azide (BA), in two equivalents relative to the alkyne groups, was added to cap unreacted alkynes and prevent potential side reactions. After further stirring for 12 h, the reaction mixture was diluted with THF and passed through a neutral alumina column to remove the copper catalyst. The filtrate was then concentrated to 3 mL via rotary evaporation and precipitated by dropwise addition into a mixture of water and methanol (1:1, v/v) under stirring. The precipitate was collected and dried under vacuum at 35 °C to afford the final product. The ^{1}H NMR spectra of intermediate and final products (Figure S21d) revealed characteristic signals from both the PtBA-derived backbone and PS side chains, consistent with the expected grafted structure. Theoretically, the low absolute

concentration of residual terminal azides renders the probability of intermolecular collision with a pendant alkyne negligible. Combined with conventional and QD-GPC profiles (Figure S22), the grafted tadpole copolymers exhibit no discernible high-molecular-weight shoulders or insoluble fractions that would indicate significant crosslinking or interchain coupling. These results confirm the successful preparation of higher-molar-mass species, supporting the synthesis of the tadpole-shaped brush polymer.

5) Modifications of tadpole polymer

5-1 Bromination of tadpole polystyrene[7]

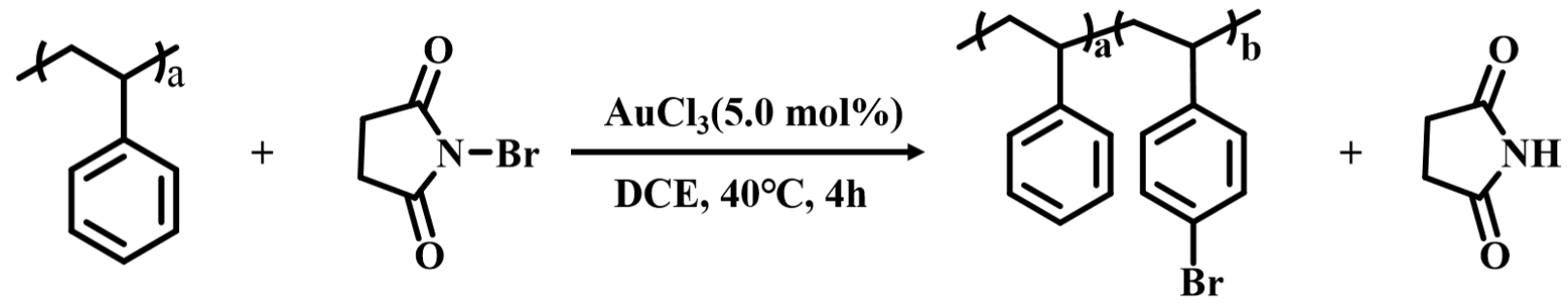

In a pre-dried 40 mL glass vial, tadpole polystyrene (t-PS_{1633}, 520 mg) was dissolved under stirring in a glovebox. A pre-homogenized solution of $AuCl_3$ (76.0 mg, 0.25 mmol) in DCE (25mL) was swiftly introduced via syringe. After complete dissolution of PS, recrystallized NBS (890 mg, 1.0 equiv) was added portionwise. The vial was immediately sealed, and stirred magnetically at 40 °C for 4 h. The mixture was then diluted with DCM, filtered through a silica gel column (10 cm height), concentrated under reduced pressure, and precipitated into cold methanol, yielding white flocculent brominated polystyrene(t-PS_{1633}-$Br_{0.94}$). Due to the introduction of bromine atoms, the ^1H NMR spectrum of brominated polystyrene showed a significant reduction in the intensity of aromatic proton signals. The degree of bromination was

calculated to be 94% based on the equation: Br% = $[5 - (3 \times S_{aromatic, Br}/S_{aliphatic})] \times 100\%$ = 94%. Consistent with successful functionalization, the GPC trace displayed a noticeable shift to lower elution volume, indicative of increased hydrodynamic volume. FTIR spectroscopy revealed new absorptions at 820 and 1074 cm^{-1}, attributed to C–Br stretching vibrations, along with an enhanced band at 1009 cm^{-1}. A concomitant decrease in the intensity of the aromatic and aliphatic C–H stretching vibrations between 3100 cm^{-1} and 2987 cm^{-1} was also observed. Detailed spectroscopic data are provided in Figure S26.

5-2 Derivatizations of brominated polystyrene

5-2-1 General procedure for sonogashira coupling

In an argon-filled glovebox, a mixture of t-PS_{1633}-$Br_{0.94}$ (mass and molar amount as specified below), the respective alkyne (as specified below), $Pd(PPh_3)_4$ (15.0 mg, 0.013 mmol, 0.05 equiv), CuI (2.5 mg, 0.013 mmol, 0.05 equiv), and iPr_2NH (132.6 mg, 1.3 mmol, 5.0 equiv) were placed in a 4 mL clear screw vial with a magnetic stirring bar. The vial was capped with a black closed-topped cap and PTFE thread seal tape, then removed from the glovebox. The vial was then placed in an oil bath at 65 °C for 24 h. After cooling to room temperature, the reaction mixture was diluted with DCM (10 mL) and filtered through a short plug of silica gel. The filtrate was concentrated by rotary evaporation, redissolved in 1 mL DCM, and precipitated into methanol (5 mL). The precipitation process was repeated once to afford the purified polymer.

Sonogashira coupling with phenylacetylene **(P1)**

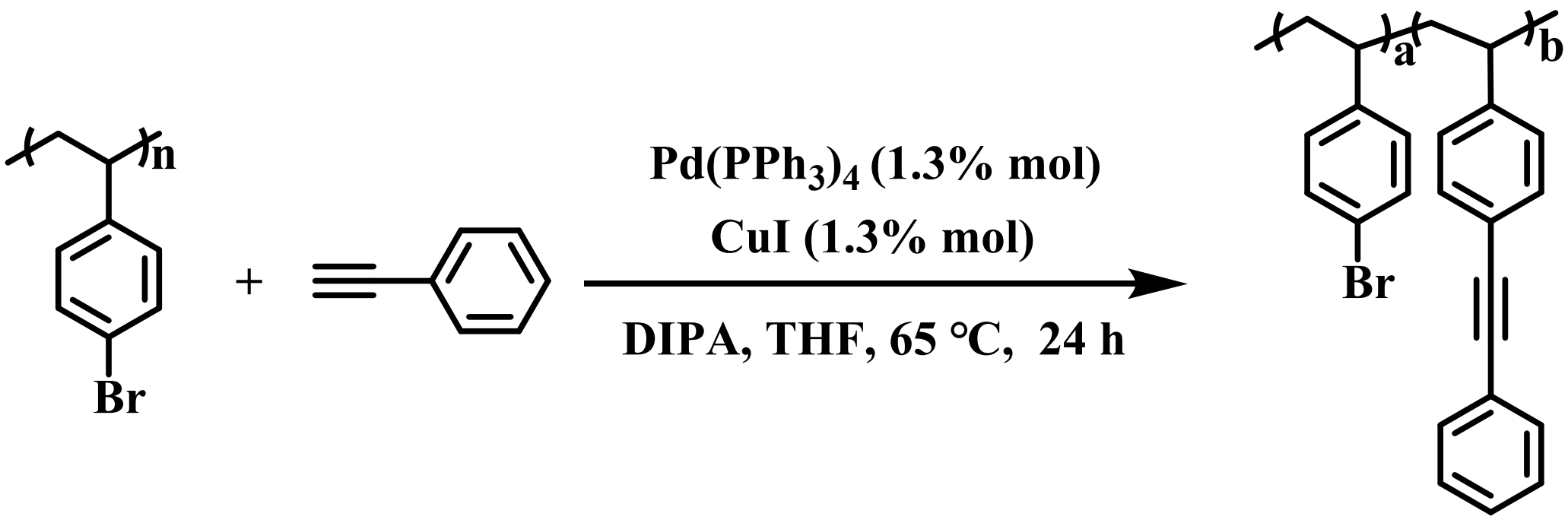

P1

Reaction scale: *t*-PS_{1633}-$Br_{0.94}$ (50.0 mg, 0.26 mmol Br).

The respective alkyne: phenylacetylene (53.1 mg, 0.52 mmol, 2.0 equiv).

Yield: 51.9 mg.

Appearance: dark green powder.

^{1}H NMR (600 MHz, dichloromethane-d2): δ 7.68–7.30 (br, $C_6\underline{H}_4$), 7.29–6.76 (br, $C_6\underline{H}_4$), 6.75–5.99 (br, $C_6\underline{H}_4$), 2.21–1.51 (br, 1H, C$\underline{H}$$CH_2$), 1.50–0.65 (br, 2H, CHC$\underline{H}_2$).

The ^{1}H NMR spectrum of the product is shown in Figure S27.

Sonogashira coupling with trimethylsilylacetylene **(P2)**

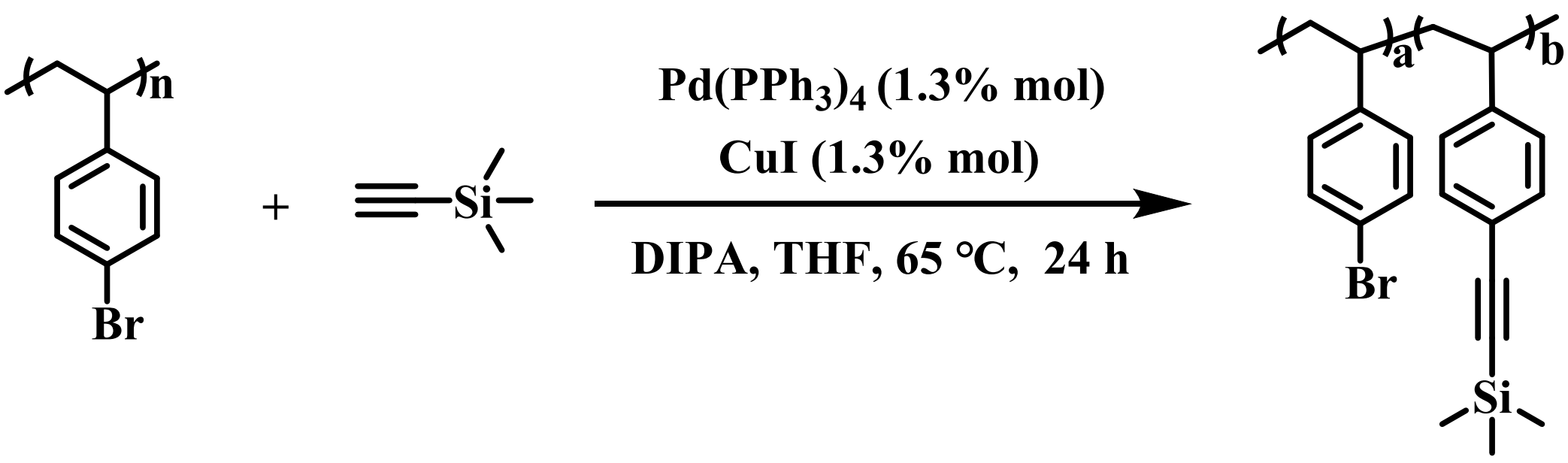

P2

Reaction scale: *t*-PS_{1633}-$Br_{0.94}$ (50 mg, 0.26 mmol Br).

The respective alkyne: trimethylsilylacetylene (51.0 mg, 0.52 mmol, 2.0 equiv).

Yield: 15.0 mg.

Appearance: pale yellow powder.

^{1}H NMR (500 MHz, Chloroform-d): δ 7.25–6.83 (br, $C_6\underline{H}_4$), 6.81–6.00 (br, $C_6\underline{H}_4$), 2.07–1.48 (br, 1H, $C\underline{H}CH_2$), 1.47–0.67 (br, 2H, $CHC\underline{H}_2$). The ^{1}H NMR spectrum of the product is shown in Figure S28.

Grafting modification of P2 with *l*-PS_{16}-N_3 **(P3)**

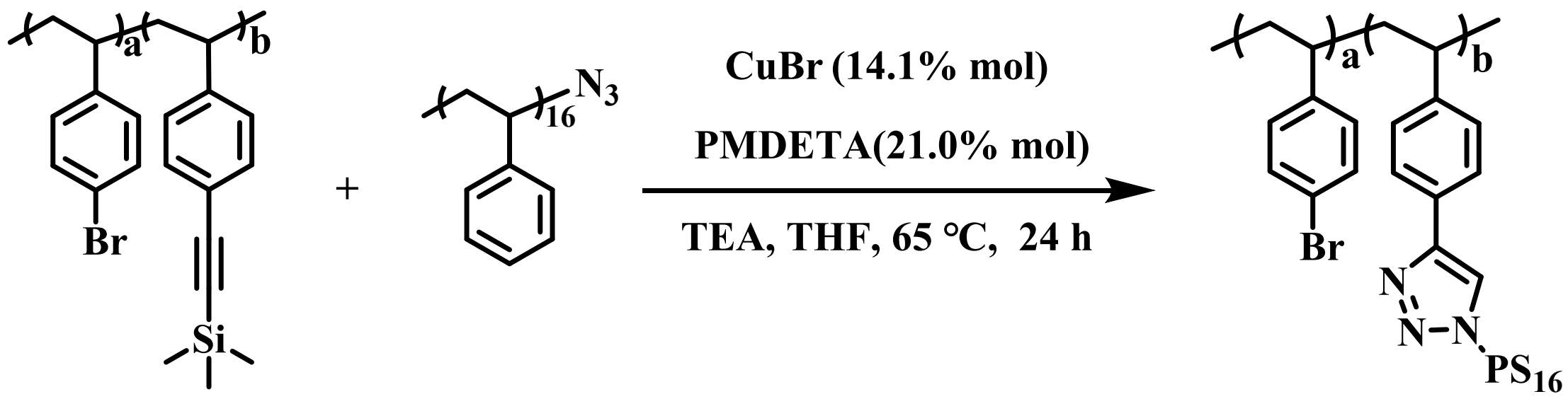

P2 **P3**

In an argon-filled glovebox, a mixture of **P2** (50.0 mg, 0.07 mmol, 1.0 equiv), an azide-terminated linear polystyrene (*l*-PS_{16}-N_3, 224.0 mg, 0.14 mmol, 2.0 equiv), triethylamine (TEA, 21.2 mg, 0.21mmol, 3.0 equiv), CuBr (20.3 mg, 0.14 mmol, 3.0 equiv), PMDETA (36.3 mg, 0.21 mmol, 3.0 equiv) and DMF (2 mL) were placed in a 4 mL clear screw vial with a magnetic stirring bar. The vial was then placed in a metal heating block at 40 °C for 24 h, during which the trimethylsilyl (TMS) protecting group on **P2** was removed in situ by triethylamine, thus generating the reactive terminal alkyne for the copper-catalyzed azide-alkyne cycloaddition with l-PS_{16}-N_3. Then the reaction mixture was diluted with DCM (10 mL), and filtered through a short plug of neutral alumina. The filtrate was concentrated by rotary evaporation and dissolved in 1 mL THF, and methanol/water (1:1) was added to precipitate the polymer. The precipitation process was repeated one more time to ensure complete removal of small molecules trapped in the polymer **P3**.

Yield: 54.4 mg.

Appearance: yellow powder.

^{1}H NMR (500 MHz, Chloroform-d): δ 7.57–6.79 (br, $C_6\underline{H}_4$), 6.78–5.97 (br, $C_6\underline{H}_4$), 5.32–4.82 (s, $NC\underline{H}CH_2$) 2.82–.2.44 (br, 1H, $C\underline{H}CH_2$), 2.43–.2.11 (br, 1H, $C\underline{H}CH_2$), 2.10–1.75 (br, 1H, $C\underline{H}CH_2$), 1.74–0.65 (br, 2H, $CHC\underline{H}_2$). The ^{1}H NMR spectrum of the product is shown in Figure S29.

5-2-2 General procedure for Suzuki-Miyaura coupling

In an argon-filled glovebox, a mixture of *t*-PS_{1633}-$Br_{0.94}$ (50 mg, 0.26 mmol Br, 1.0 equiv), the respective boronic acid/ester (as specified below), palladium catalyst (as specified below), base (as specified below), and a THF/H_2O mixture (ratio as specified below) were placed in a 10 mL clear screw vial with a magnetic stirring bar. The vial was sealed with a black cap and PTFE thread seal tape, then removed from the glovebox. The reaction mixture was stirred in an oil bath at the specified temperature for 24 h. After cooling to room temperature, the mixture was diluted with DCM and filtered through a short silica gel plug. The filtrate was concentrated by rotary evaporation, redissolved in 1 mL of DCM, and precipitated into cold methanol. The precipitation was repeated once to afford the purified polymer.

Suzuki-Miyaura coupling with potassium vinyltrifluoroborate **(P4)**

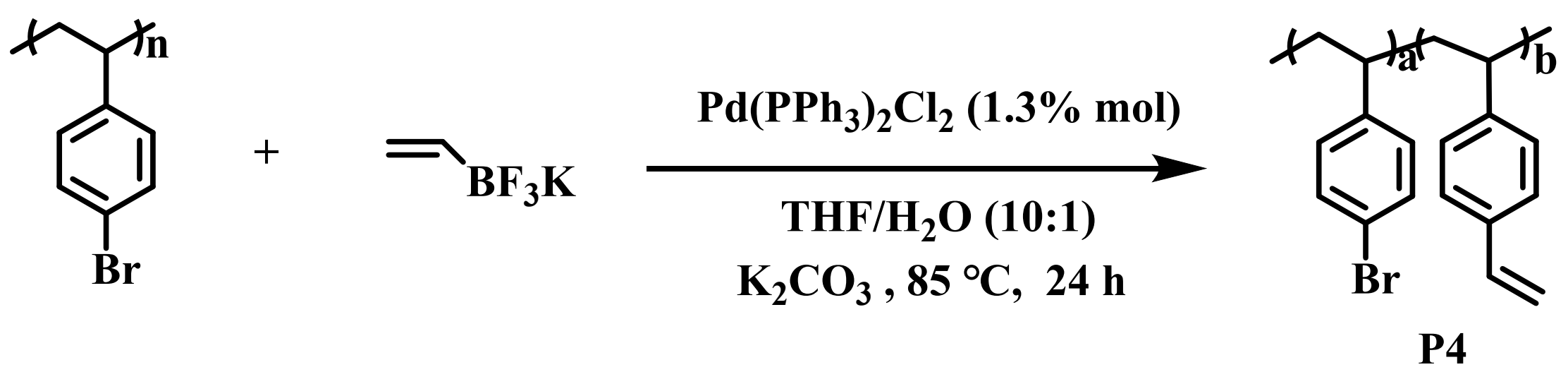

Reaction conditions: potassium vinyltrifluoroborate (104.5 mg, 0.78 mmol, 3.0 equiv), $Pd(PPh_3)_2Cl_2$ (9.1 mg, 0.013 mmol, 0.05 equiv), K_2CO_3 (71.8 mg, 0.52 mmol, 2.0 equiv), THF/H_2O (4.0 mL/0.4 mL), 85 °C.

Yield: 59.2 mg.

Appearance: yellow powder.

^{1}H NMR (500 MHz, Chloroform-d): δ 7.53–6.86 (br, $C_6\underline{H}_4$), 6.85–6.28 (br, $C_6\underline{H}_4$), 5.39–5.22 (m, $CH{=}C\underline{H}_2$), 2.15–1.69 (br, 1H, $C\underline{H}CH_2$), 1.68–0.68 (br, 2H, $CHC\underline{H}_2$). The ^{1}H NMR spectrum of the product is shown in Figure S30.

Suzuki-Miyaura coupling with phenylboronic acid **(P5)**

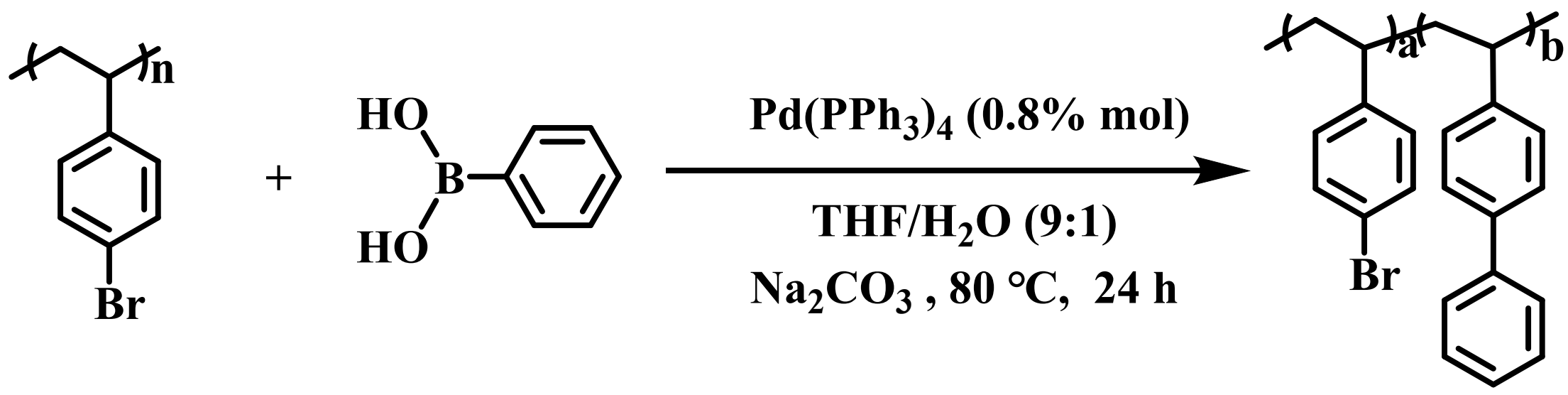

P5

Reaction conditions: phenylboronic acid (63.4 mg, 0.52 mmol, 2.0 equiv), $Pd(PPh_3)_4$ (9.0 mg, 0.0078 mmol, 0.03 equiv), Na_2CO_3 (55.1 mg, 0.52 mmol, 2.0 equiv), THF/H_2O (3.6 mL/0.4 mL), 80 °C.

Yield: 15.1 mg.

Appearance: off-white powder.

^{1}H NMR (500 MHz, Chloroform-d): δ 7.79–7.56(br, $C_6\underline{H}_4$), 7.55–7.27 (br, $C_6\underline{H}_4$), 7.13–6.79 (br, $C_6\underline{H}_4$), 6.69–6.19 (br, $C_6\underline{H}_4$), 2.20–1.46(br, 1H, $C\underline{H}CH_2$), 1.45–0.57 (br, 2H, $CHC\underline{H}_2$). The ^{1}H NMR spectrum of the product is shown in Figure S31.

Suzuki-Miyaura coupling with 4-(methoxycarbonyl)phenylboronic acid **(P6)**

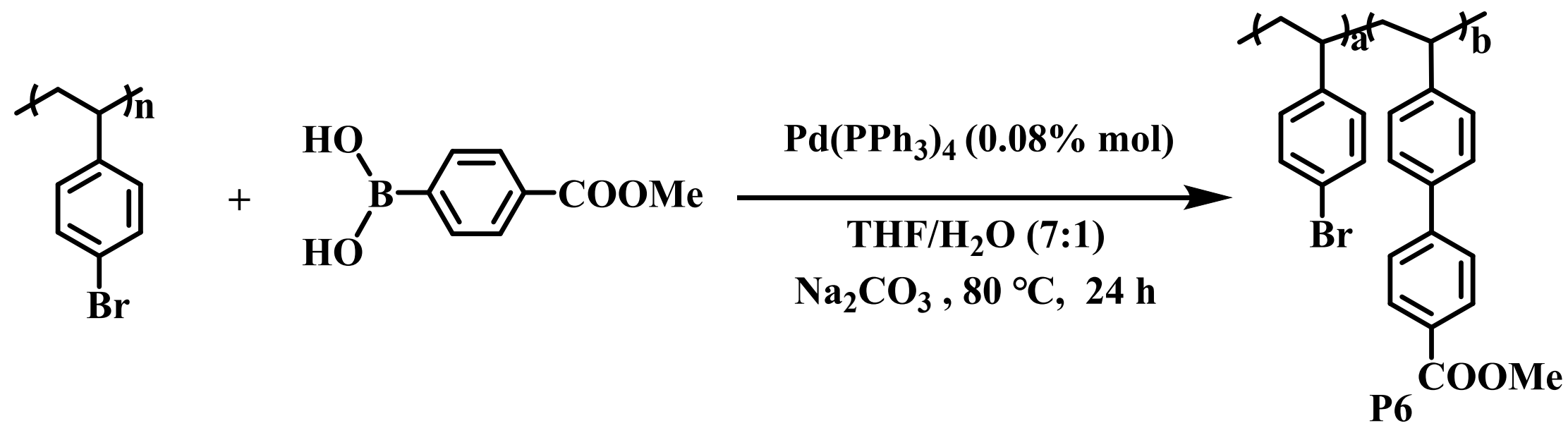

Reaction conditions: 4-(methoxycarbonyl)phenylboronic acid (93.6 mg, 0.52 mmol, 2.0 equiv), $Pd(PPh_3)_4$ (9.0 mg, 0.0078 mmol, 0.03 equiv), Na_2CO_3 (55.1 mg, 0.52 mmol, 2.0 equiv), THF/H_2O (3.5 mL/0.5 mL), 80 °C.

Yield: 59.2 mg

Appearance: off-white powder.

^{1}H NMR (500 MHz, Chloroform-d): δ 8.40-7.86(br, $C_6\underline{H}_4$), 7.83–7.48 (br, $C_6\underline{H}_4$), 7.45–6.75 (br, $C_6\underline{H}_4$), 6.73–5.71 (br, $C_6\underline{H}_4$), 3.98-3.72 (s, OCH_3), 3.70-3.53 (s, OCH_3), 2.23–1.42 (br, 1H, $C\underline{H}CH_2$), 1.41–0.58 (br, 2H,$CHC\underline{H}_2$). The ^{1}H NMR spectrum of the product is shown in Figure S32.

5-2-3. General procedure for Heck coupling

In an Argon-filled glovebox, a mixture of *t*-PS_{1633}-$Br_{0.94}$ (mass and molar amount as specified below), the respective alkene (as specified below), $Pd(dba)_2$ (14.9 mg, 0.026 mmol, 0.05 equiv), P(*t*-Bu)$_3$ (1.0 M in toluene, 52 µL, 0.052 mmol, 0.1 equiv), NaOAc (64.0 mg, 0.78 mmol, 1.5 equiv), and DMF (2 mL) were placed in a 4 mL clear screw vial with a magnetic stirring bar. The vial was capped with a black closed-topped cap and PTFE thread seal tape, then removed from the glovebox. The vial was then

placed in an oil bath at 80 °C for 24 h. After cooling to room temperature, the reaction mixture was diluted with DCM (10 mL) and filtered through a short plug of silica gel. The filtrate was concentrated by rotary evaporation, redissolved in 1 mL DCM, and precipitated into methanol (5 mL). The precipitation process was repeated once to afford the purified polymer.

Heck reaction with methyl acrylate **(P7)**

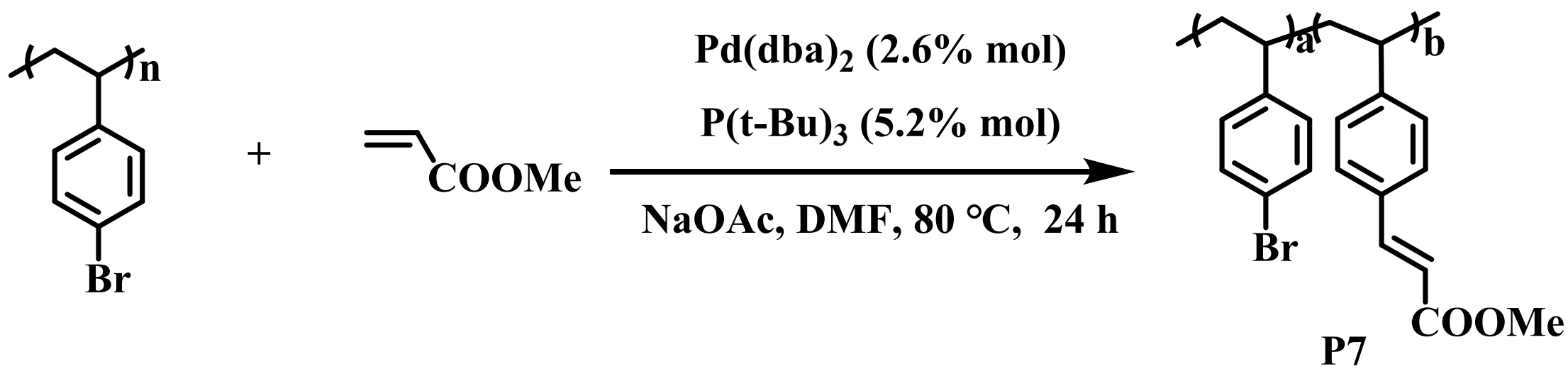

Reaction scale: *t*-PS_{1633}-$Br_{0.94}$ (100 mg, 0.52 mmol Br).

the respective alkene: Methyl acrylate (89.4 mg, 1.04 mmol, 2.0 equiv).

Yield: 73.2 mg (67%).

Appearance: off-white powder.

^{1}H NMR (500 MHz, Chloroform-d): 7.87–7.32 (br, $C_6\underline{H}_4$), 7.31–6.77 (br, $C_6\underline{H}_4$), 6.76–5.92 (br, $C_6\underline{H}_4$), 3.96-3.74 (s, OCH_3), 3.72-3.52 (s, OCH_3), 2.22–1.53 (br, 1H, $C\underline{H}CH_2$), 1.52–0.74 (br, 2H, $CHC\underline{H}_2$). The ^{1}H NMR spectrum of the product is shown in Figure S33.

Heck reaction with *tert*-butyl acrylate **(P8)**

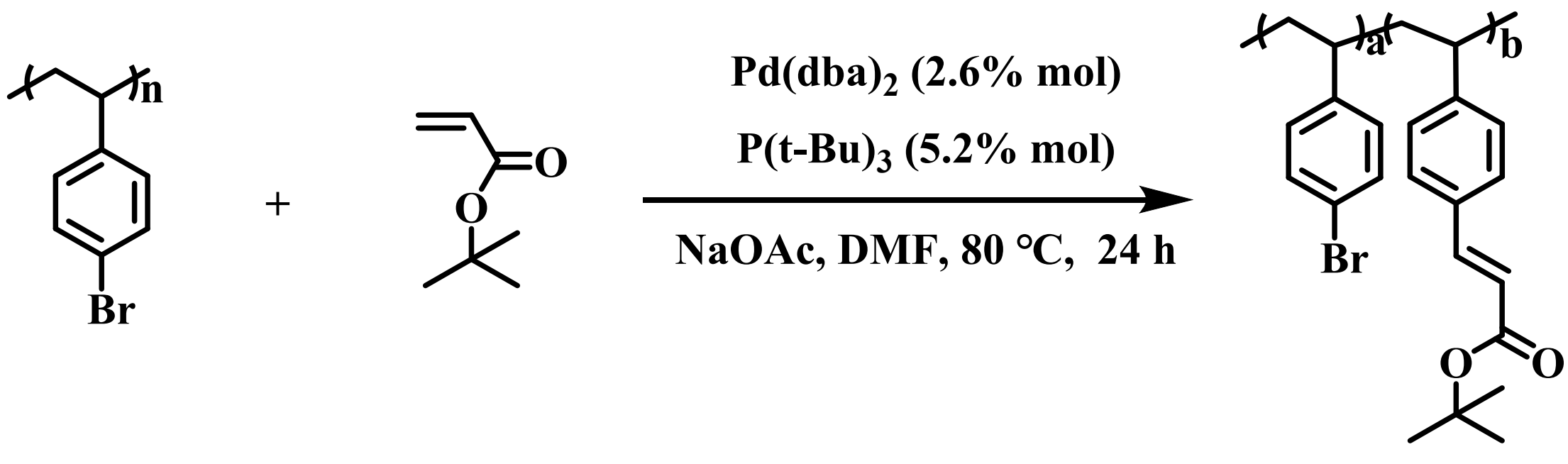

P8

Reaction scale: *t*-PS_{1633}-$Br_{0.94}$ (50 mg, 0.26 mmol Br).

Conditions: *tert*-Butyl acrylate (66.6 mg, 0.52 mmol, 2.0 equiv).

Yield: 22.0 mg.

Appearance: off-white powder.

^{1}H NMR (500 MHz, Chloroform-d): δ 7.67–7.40 (br, $C_6\underline{H}_4$), 7.39–6.85 (br, $C_6\underline{H}_4$), 6.84–5.92 (br, $C_6\underline{H}_4$), 2.34–2.11 (br, 1H, C$\underline{H}$$CH_2$), 2.10–1.64(br, 1H, C$\underline{H}$$CH_2$), 1.63–1.30 (br, –C(C$\underline{H}_3$), CHC$\underline{H}_2$), 1.29–0.73 (br, CHC$\underline{H}_2$). The ^{1}H NMR spectrum of the product is shown in Figure S34.

Heck reaction with 3-butenenitrile **(P9)**

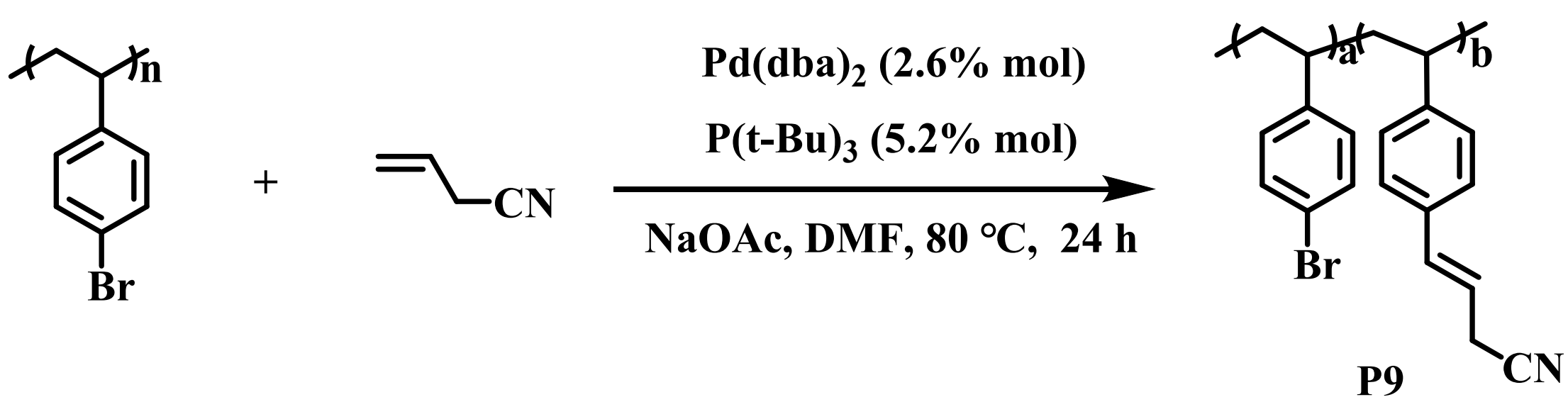

P9

Reaction scale: *t*-PS_{1633}-$Br_{0.94}$ (50 mg, 0.26 mmol Br).

Conditions: 3-butenenitrile (34.9 mg, 0.52 mmol, 2.0 equiv).

Yield: 14.7 mg.

Appearance: gray powder.

^{1}H NMR (500 MHz, Chloroform-d): δ 8.03–6.90 (br, $C_6\underline{H}_4$), 6.89–6.20(br, $C_6\underline{H}_4$), 6.17–5.75 (br, -C$\underline{H}$=C$\underline{H}$-CH_2-CN), 3.50–3.11(s, -CH=CH-C$\underline{H}_2$-CN), 2.45–2.17 (br, 1H, C$\underline{H}$$CH_2$), 2.16–1.64 (br, 1H, C$\underline{H}$$CH_2$), 1.63–0.54 (br, CHC$\underline{H}_2$). The ^{1}H NMR spectrum of the product is shown in Figure S35.

5-2-4. Miyaura borylation with $B_2(pin)_2$ (P10)

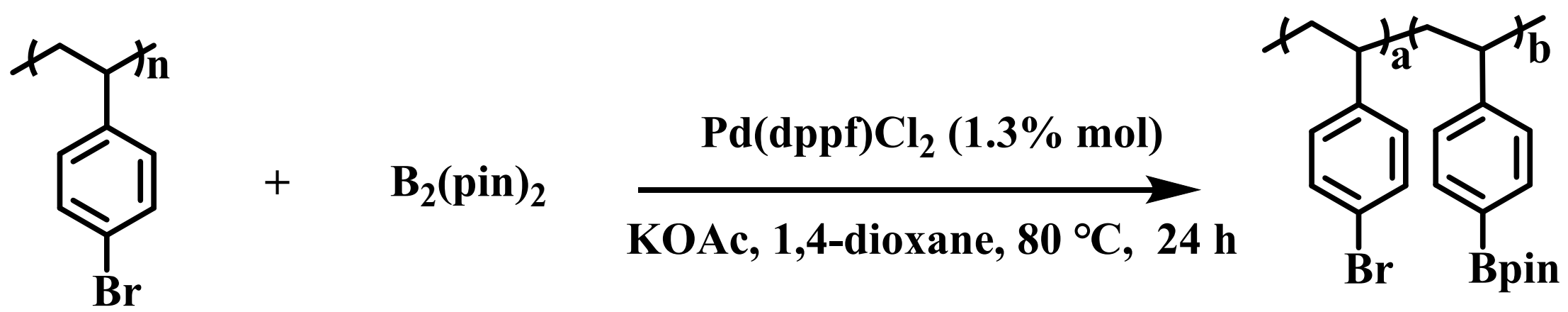

P10

In an argon-filled glovebox, a mixture of *t*-PS_{1633}-$Br_{0.94}$ (50 mg, 0.26 mmol, 1.0 equiv), $B_2(pin)_2$ (132.0 mg, 0.52 mmol, 2.0 equiv), $Pd(dppf)Cl_2$ (9.5 mg, 0.013 mmol, 0.05 equiv), KOAc (76.4 mg, 0.78 mmol, 3.0 equiv) and 1,4-dioxane (4.0 mL) were placed in a 10 mL clear screw vial with a magnetic stirring bar. The vial was sealed and removed from the glovebox. The reaction mixture was stirred in an oil bath at 80 °C for 24 h. After cooling to room temperature, the mixture was diluted with DCM (10 mL) and filtered through a short silica gel plug. The filtrate was concentrated, redissolved in 1-2 mL of DCM, and precipitated into cold methanol (5 mL). The precipitation was repeated once to afford polymer **P10**.

Appearance: off-white powder.

Yield: 59.8 mg.

^{1}H NMR (500 MHz, Chloroform-d): δ 8.18–7.26 (br, $C_6\underline{H}_4$), 7.15–6.77 (br, $C_6\underline{H}_4$), 6.76–6.00 (br, $C_6\underline{H}_4$), 2.97–2.46 (C$\underline{H}$$CH_2$), 2.45–0.97 (C$\underline{H}$$CH_2$, CHC$\underline{H}_2$, OC(C$\underline{H}_3$)$_2$),

0.96–0.73 (br, CHC$\underline{H}_2$). The ^{1}H NMR spectrum of the product is shown in Figure S36.

5-2-5. Amination with LiHMDS (P11)

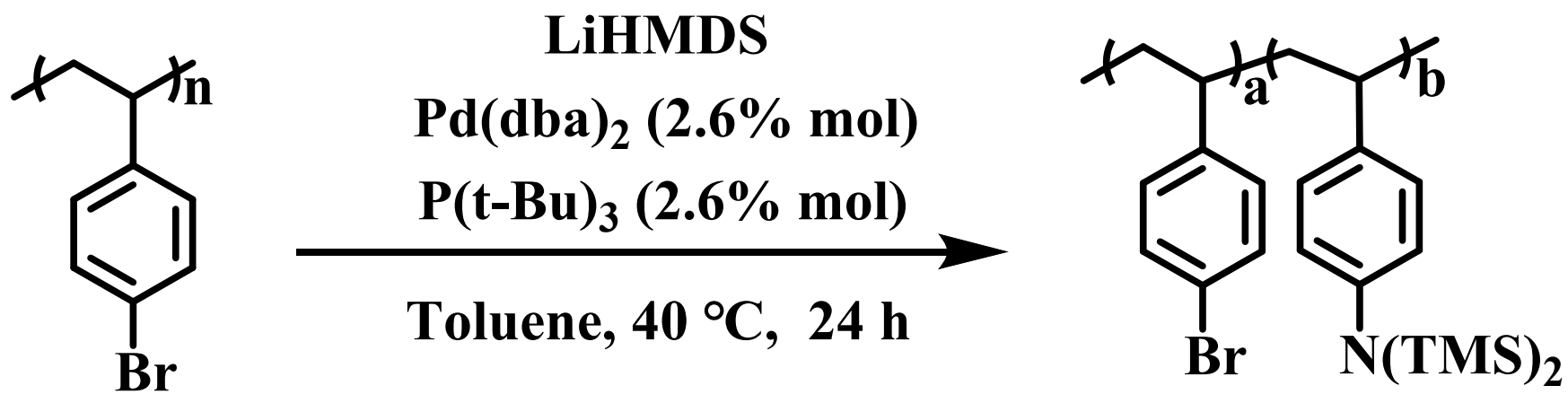

P11

In an argon-filled glovebox, a mixture of *t*-PS_{1633}-$Br_{0.94}$ (100 mg, 0.52 mmol, 1.0 equiv), LiHMDS (1.0 M in THF, 570 µL, 0.57 mmol, 1.1 equiv), $Pd(dba)_2$ (15.0 mg, 0.026 mmol, 0.05 equiv), $P(t\text{-}Bu)_3$ (1.0 M in toluene, 26 µL, 0.026 mmol, 0.05 equiv) and toluene (3.0 mL) were placed in a 10 mL clear screw vial with a magnetic stirring bar. The vial was sealed and removed from the glovebox. The reaction mixture was stirred in an oil bath at 40 °C for 24 h. After cooling to room temperature, the mixture was diluted with DCM (10 mL) and a few drops of triethylamine, then filtered through a short silica gel plug. The filtrate was concentrated, redissolved in 1 mL of DCM, and precipitated into methanol (5 mL) containing a few drops of triethylamine. The precipitation was repeated once to afford the purified polymer.

Yield: 57.6 mg.

Appearance: off-white powder.

^{1}H NMR (500 MHz, Chloroform-d): δ 7.44–6.76 (br, $C_6\underline{H}_4$), 6.72–5.97 (br, $C_6\underline{H}_4$), 2.06-1.42 (br, 1H, C$\underline{H}$$CH_2$), 1.41–0.56 (br, CHC$\underline{H}_2$), -0.05–-0.20 (s, $N(Si(C\underline{H}_3)_3)_2$). The ^{1}H NMR spectrum of the product is shown in Figure S37.

(Polymers **P1**, **P2**, **P3**... are the products from the post-polymerization modification of *t*-PS_{1633}-$Br_{0.94}$)

5-3 General procedure for TMG-Catalyzed side-chain grafting of tadpole poly(acrylic acid)

In a standard procedure, a mixture of poly(acrylic acid) (PAA, 0.05 g, 0.7 mmol of repeating unit), the respective alkyl bromide (1.4 mmol, see Table S12 below), and DMF (1.5 mL) was stirred in a glovebox until dissolution. 1,1,3,3-Tetramethylguanidine (TMG, 0.163 g, 1.4 mmol) was added dropwise over 30 min, and the reaction was stirred at 45 °C for 8 h. Iodomethane (0.098 g, 0.7 mmol) was then added, and stirring was continued at 45 °C for 2 h to cap residual acids. The product was isolated by dialysis against dichloromethane using a dialysis membrane with a molecular weight cut-off of 2 kDa and purified by rotary evaporation followed by drying under vacuum. The synthetic route is depicted in Figure S20a. All other side-chain modifications were performed following this general procedure with the same molar equivalents, differing only in the grafting reagent used. For specific reagents, please refer to Table S12. The ^{1}H NMR spectrum of the product is shown in Figure S38 – S46.

5-4. A general blueprint for functional tadpole polymers via backbone-specific cyclization[8, 9]

The intramolecular cyclization strategy demonstrates potential generality owing to its compatibility with orthogonal modification pathways, as shown in Figure S47. The

methodology established for inert aromatic backbones—exemplified by PS through sequential Au-catalyzed bromination and Pd-catalyzed cross-coupling—can be directly extended to other vinyl aromatic systems such as poly(vinylpyridine) for constructing coordination-active topological structures or poly(phenylacetylene) for developing conjugated materials with tailored electronic properties. Concurrently, the active ester platform demonstrated with PtBA, which enables versatile functionalization through hydrolysis and esterification, proves readily transferable to other acrylate and methacrylamide scaffolds. This allows for precise incorporation of diverse side chains, including fluorinated groups for surface modification and PEG segments for enhanced biocompatibility. Collectively, this work establishes a foundational framework for creating an expansive library of functional tadpole polymers that extends well beyond the specific systems presented in this study.

6) Supporting Tables

Table S1. Experiment conditions for *l*-PS_n

	Molar ratios						
Sample	I	St	Me_6tren	$Sn(EH)_2$	CuBr	T/℃	t/h
l-PS_{56}	1	500	0.4	0.4	0.1	80	3
l-PS_{218}	1	1600	0.4	0.4	0.1	80	4
l-PS_{538}	1	5000	1	1	0.1	80	8
l-PS_{1633}	1	10000	1	1	0.1	80	25
l-PS_{3766}	1	25000	1	1	0.1	80	43

Table S2. Experiment conditions for *l*-$PtBA_n$

	Molar ratios						
Sample	I	tBA	Me_6tren	$Sn(EH)_2$	CuBr	T/℃	t/h
l-$PtBA_{63}$	1	300	0.2	0.2	0.1	70	3
l-$PtBA_{214}$	1	600	0.2	0.2	0.1	70	4
l-$PtBA_{593}$	1	1000	0.2	0.2	0.1	70	6
l-$PtBA_{793}$	1	2000	0.2	0.2	0.1	70	8
l-$PtBA_{1587}$	1	5000	0.2	0.2	0.1	70	12
l-$PtBA_{3174}$	1	10000	0.2	0.2	0.1	70	15
l-$PtBA_{5283}$	1	15000	0.2	0.2	0.1	70	18
l-$PtBA_{7936}$	1	25000	0.2	0.2	0.1	70	24

Table S3. Summary of batch scales, isolated yields, average yield, and standard deviation for representative ultrahigh-molecular-weight tadpole polymers

Sample	Batch	Batch scale (mg)	Isolated yield (%)	Mean yield (%)	Standard deviation (%)
t-PS_{1633}	1	500	92.8	90.5	2.4
	2	500	90.7		
	3	500	88.0		
t-$PtBA_{1587}$	1	500	89.5	88.0	1.4
	2	500	87.8		
	3	500	86.7		

Table S4. Molecular parameters of *l*-PS_n and *t*-PS_n samples from conventional GPC and DLS

Sample	$M_n^a/10^4$/(Da)	$M_w^a/10^4$/(Da)	$M_p^a/10^4$/(Da)	$Đ^a$	$M_{rel, p, t}/M_{rel, p, l}$	$\langle R_h \rangle^b$/nm	C^{*c}/(g/L)
l-PS_{56}	0.56	0.58	0.62	1.11	0.87	1.9	107.2
t-PS_{56}	0.49	0.50	0.54	1.13			
l-PS_{218}	2.09	2.21	2.31	1.11	0.82	3.7	45.7
t-PS_{218}	1.71	1.81	1.89	1.12			
l-PS_{538}	4.99	5.78	5.53	1.16	0.82	6.0	24.7
t-PS_{538}	4.09	4.74	4.53	1.15			
l-PS_{1633}	14.81	17.25	17.07	1.15	0.89	11.3	12.3
t-PS_{1633}	12.97	15.44	15.27	1.17			
l-PS_{3766}	32.21	38.64	39.31	1.25	0.92	19.1	7.4
t-PS_{3766}	29.06	36.11	36.36	1.25			

[a] The data were measured by conventional GPC System, and the molecular weights reported are apparent molecular weights relative to the calibration standards.

[b] The hydrodynamic radii(R_h) of *l*-PS_n were measured by DLS.

[c] The overlap concentration C* were calculated by $C^* = 3M / (4\pi N_A R_g^3)$

Table S5. Molecular parameters of *l*-PS_n and *t*-PS_n obtained from QD-GPC

Sample	$M_{p, abs}^a/10^4$/(g/mol)	$[\eta]_p^a$/(dL/g)	$R_{g, p}^a$/nm
l-PS_{218}	2.37	0.17	\
t-PS_{218}	2.36	0.14	\
l-PS_{538}	5.63	0.32	\
t-PS_{538}	5.35	0.29	\
l-PS_{1633}	18.93	0.68	13.1
t-PS_{1633}	18.68	0.63	11.0
l-PS_{3766}	45.42	1.34	24.9
t-PS_{3766}	43.59	1.24	23.6

[a] The data were measured by QD-GPC System, and the molecular weights reported are absolute molecular weights.

Table S6. Molecular parameters of *l*-$PtBA_n$ and *t*-$PtBA_n$ samples from conventional GPC and DLS

Sample	$M_n^a/10^4$/(g/mo	$M_w^a/10^4$/(g/mo	$M_p^a/10^4$/(g/mo	$Đ^a$	$M_{rel,\ p,\ t}/M_{rel,\ p,\ l}$	$\langle R_h \rangle^b$/nm
l-$PtBA_{63}$	0.64	0.75	0.77	1.20	0.90	2.1
t-$PtBA_{63}$	0.61	0.73	0.69	1.20		
l-$PtBA_{214}$	2.38	2.61	2.59	1.08	0.86	3.8
t-$PtBA_{214}$	2.00	2.22	2.23	1.11		
l-$PtBA_{593}$	5.49	6.53	6.83	1.20	0.86	
t-$PtBA_{593}$	5.00	5.89	5.89	1.22		
l-$PtBA_{793}$	8.10	10.12	11.40	1.24	0.91	7.8
t-$PtBA_{793}$	8.36	10.41	10.40	1.25		
l-$PtBA_{1587}$	12.76	15.89	15.94	1.24	0.85	
t-$PtBA_{1587}$	11.16	13.61	13.53	1.21		
l-$PtBA_{3174}$	36.83	50.03	55.12	1.38	0.81	12.5
t-$PtBA_{3174}$	30.03	43.13	44.67	1.39		
l-$PtBA_{5283}$	49.99	66.44	72.65	1.33	0.91	
t-$PtBA_{5283}$	45.00	61.10	66.25	1.38		
l-$PtBA_{7936}$	42.88	110.70	125.73	1.70	0.71	25.2
t-$PtBA_{7936}$	31.38	81.03	89.43	1.70		

[a] The data were measured by conventional GPC System, and the molecular weights reported are apparent molecular weights relative to the calibration standards.

[b] The hydrodynamic radius (R_h) of *l*-$PtBA_n$ were measured by DLS.

Table S7. Molecular parameters of *l*-$PtBA_n$ and *t*-$PtBA_n$ obtained from QD-GPC

Sample	$M_{p,\ abs}^a/10^4$/(g/mol)	$[\eta]_p^a$/(dL/g)	$R_{g,\ p}^a$/nm
l-$PtBA_{214}$	2.28	0.18	\
t-$PtBA_{214}$	2.45	0.17	\
l-$PtBA_{593}$	5.77	0.35	\
t-$PtBA_{593}$	6.29	0.32	\
l-$PtBA_{793}$	9.09	0.48	\
t-$PtBA_{793}$	9.79	0.44	\
l-$PtBA_{1587}$	15.93	0.73	13.8
t-$PtBA_{1587}$	16.42	0.61	12.0
l-$PtBA_{3174}$	35.1	1.26	20.9
t-$PtBA_{3174}$	32.7	1.08	15.6
l-$PtBA_{5283}$	61.06	1.77	36.7
t-$PtBA_{5283}$	62.26	1.63	34.7
l-$PtBA_{7936}$	79.5	1.84	49.5
t-$PtBA_{7936}$	76.8	1.75	46.0

[a] The data were measured by QD-GPC System, and the molecular weights reported are absolute molecular weights.

Table S8. Summary of peak retention volumes for *l*-PS_{3766} ($V_{e,\ l}$) and *t*-PS_{3766} ($V_{e,\ t}$) obtained from five replicate SEC injections, and the corresponding peak shift (ΔV_e)

Injection	$V_{e,\ l}$/mL	$V_{e,\ t}$/mL	ΔV_e[a]/mL
1	19.46	19.56	0.10
2	19.48	19.57	0.09
3	19.48	19.56	0.08
4	19.48	19.56	0.08
5	19.44	19.52	0.08
Mean	19.468	19.554	0.086
Standard deviation	0.018	0.020	0.009

[a] $\Delta V_e = V_{e,\,t} - V_{e,\,l}$

Table S9. Experimental parameters for molecular chain length and concentration gradient in the ring-closing reaction of tadpole polymers

Injected Concentration C_i /(g/L)	Injected Volume V / (mL)	Injection Rate r / (mL/h)	DMF (mL)	CuBr (mg)	PMDETA (μL)	Solution Concentration C_s / (g/L)
2	2	0.5	20	20	50	0.18
6	2	0.5	20	20	50	0.54
18	2	0.5	20	20	50	1.64
54	2	0.5	20	20	50	4.91
162	2	0.5	20	20	50	14.71
324	2	0.5	20	20	50	29.45

Table S10. Absolute molecular weight of unimer and multimer peaks for linear precursors (*l*-PS_n) and tadpole polymers (*t*-PS_n) under high-concentration conditions (C_i = 162 g/L)

Sample	$M_{p, abs}{}^a/10^4$/(g/mol) Unimer	$M_{p, abs}{}^a/10^4$/(g/mol) Multimer
l-PS_{218}	2.32	\
t-PS_{218}	2.38	4.18
l-PS_{538}	5.72	\
t-PS_{538}	5.74	11.48
l-PS_{1633}	19.30	\
t-PS_{1633}	21.67	40.88
l-PS_{3766}	43.88	\
t-PS_{3766}	47.77	89.80

[a] The data were measured by QD-GPC System, and the molecular weights reported are absolute molecular weights.

Table S11. Experimental conditions for polystyrene bromination

Sample	Molar ratios			T/°C	t/h
	St	NBS	$AuCl_3$		
l-PS_{1633}	1	1	0.05	40	4
t-PS_{1633}	1	1	0.05	40	4

Table S12. Functionalization reagents for poly(acrylic acid)

Alkyl Bromide	Mass Used (g)	Grafted Functionality	Product[a]
Benzyl bromide	0.239	Benzyl	D1
1-(Bromomethyl)naphthalene	0.301	Naphthalenylmethyl	D2
1-Bromododecane	0.346	Dodecyl	D3
2-(Dimethylamino)ethyl bromide hydrobromide	0.308	N,N-Dimethylaminoethyl	D4
2-Nitrobenzyl bromide	0.302	2-Nitrobenzyl	D5
Sodium 3-bromopropane-1-sulfonate	0.335	Sulfonatopropyl	D6
2-Bromoethanol	0.175	2-Hydroxyethyl	D7
Di(ethylene glycol) 2-bromoethyl methyl ether	0.295	Di(ethylene glycol)	D8
1-Bromo-2-(2-methoxyethoxy)ethane	0.269	(2-Methoxyethoxy)ethyl	D9

[a] The polymers obtained from the poly(tert-butyl acrylate) (PAA) series are labelled as D1, D2, etc., for simplicity.

Table S13. ^{1}H NMR characterization for grafted polymers D1-D9

Product[a]	Molecular Structure (Grafting Agent)	^{1}H NMR Characterization (500 MHz)[b]
D1	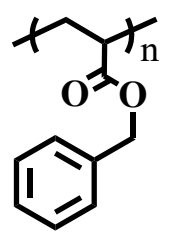	($CDCl_3$) 7.10-7.35 ($C_6\underline{H}_5CH_2$), 4.82-5.07 ($COOC\underline{H}_2$), 2.22-2.45 ($C\underline{H}CH_2$)
D2	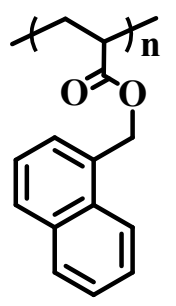	($CDCl_3$) δ 5.05-5.67 ($COOC\underline{H}_2$), 2.15-2.57 ($C\underline{H}CH_2$)

D3	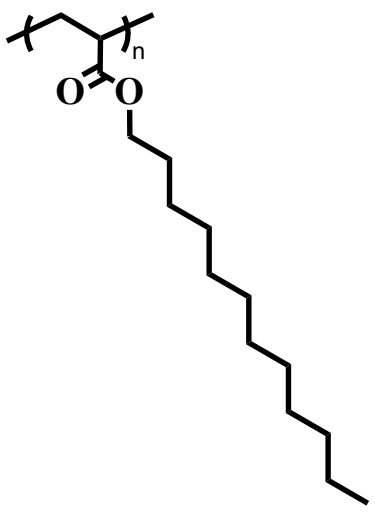	($CDCl_3$) δ 3.93-4.10 ($COOC\underline{H}_2$), 2.20-2.43 ($C\underline{H}CH_2$).
D4	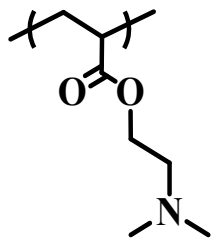	(DMSO-d_6) δ 3.82-4.20 ($COOC\underline{H}_2$), 3.62-3.82 ($COOC\underline{H}_3$).
D5	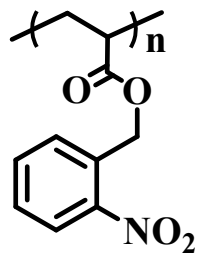	($CDCl_3$) δ 5.05-5.55 ($COOC\underline{H}_2$), 2.30-2.68 ($C\underline{H}CH_2$).
D6	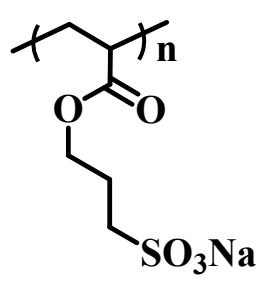	(D_2O) δ 4.00-4.35 ($COOC\underline{H}_2$), 3.51-3.82 ($COOC\underline{H}_3$).
D7	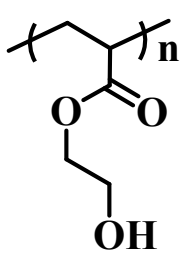	(DMSO-d_6) δ 4.73-4.90 ($CH_2CH_2O\underline{H}$), 3.96-4.15 ($COOC\underline{H}_2$) ,2.16-2.46 ($C\underline{H}CH_2$).
D8	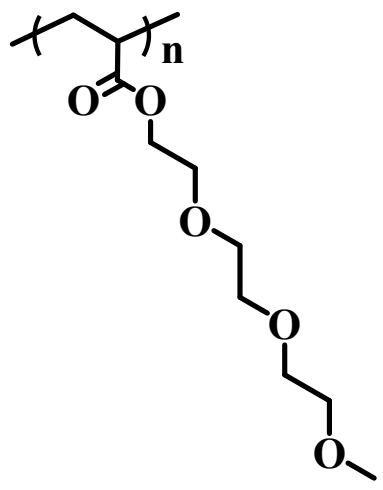	(DMSO-d_6) δ 4.00-4.18 ($COOC\underline{H}_2$), 2.17-2.38 ($C\underline{H}CH_2$).
D9	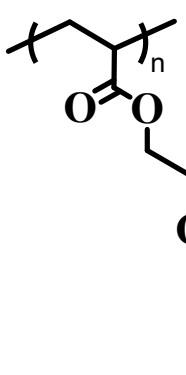	(DMSO-d_6) δ 3.99-4.15 ($COOC\underline{H}_2$), 2.15-2.36 ($C\underline{H}CH_2$).

[a] The polymers obtained from the poly(tert-butyl acrylate) (PAA) series are labelled as D1, D2, etc., for simplicity.

7) Supporting Figures

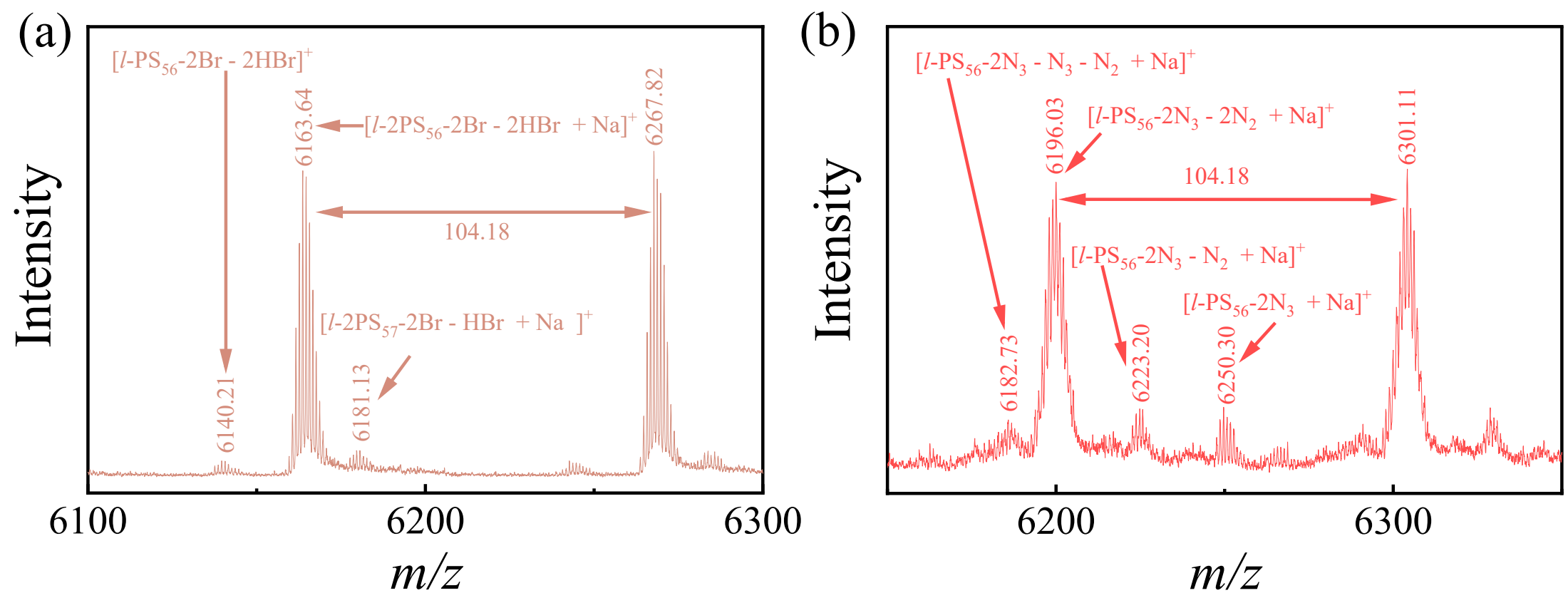

Figure S1. MALDI-TOF MS of the *l*-PS_{56} sample (a) with two bromide ends and (b) with two azide ends.

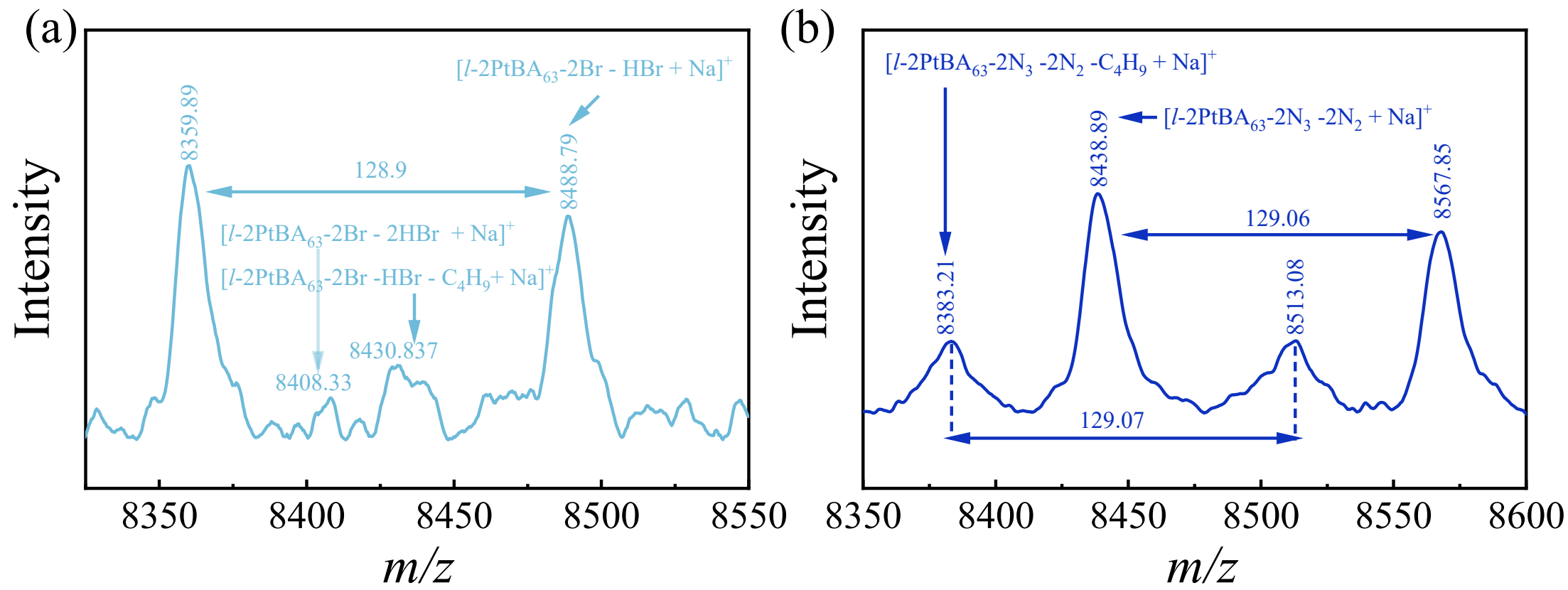

Figure S2. MALDI-TOF MS of the *l*-$PtBA_{63}$ sample (a) with two bromide ends and (b) with two azide ends.

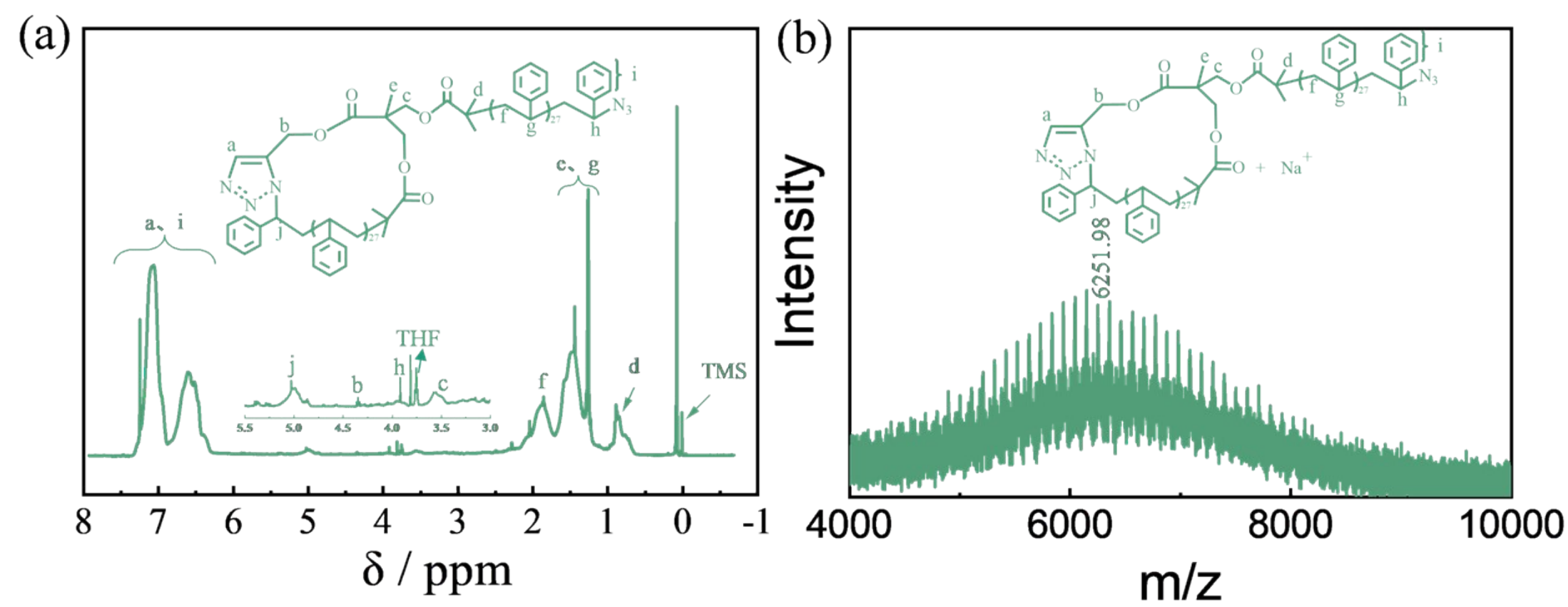

Figure S3. (a) ^{1}H NMR spectrum of *t*-PS_{56} in deuterated chloroform. (b) MALDI-TOF MS of *t*-PS_{56} sample.

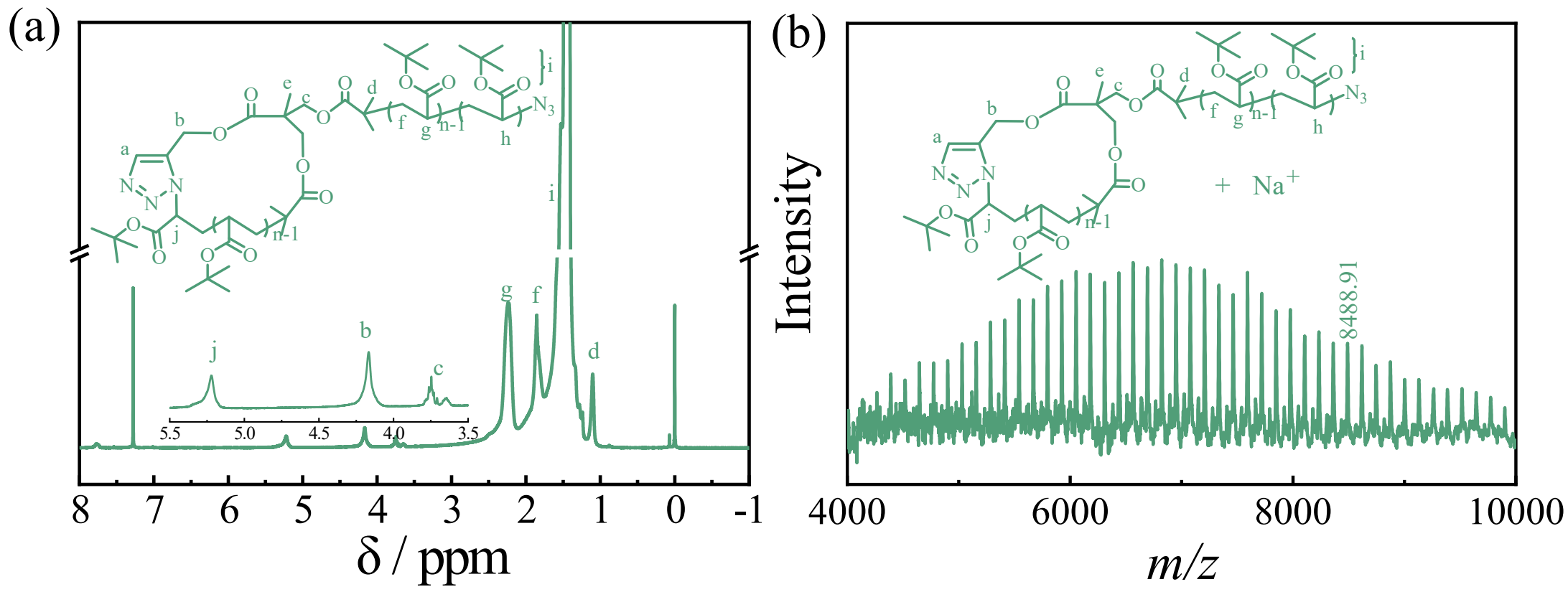

Figure S4. (a) ^{1}H NMR spectrum of t-PtBA$_{63}$ in deuterated chloroform. (b) MALDI -TOF MS of t-PtBA$_{63}$.

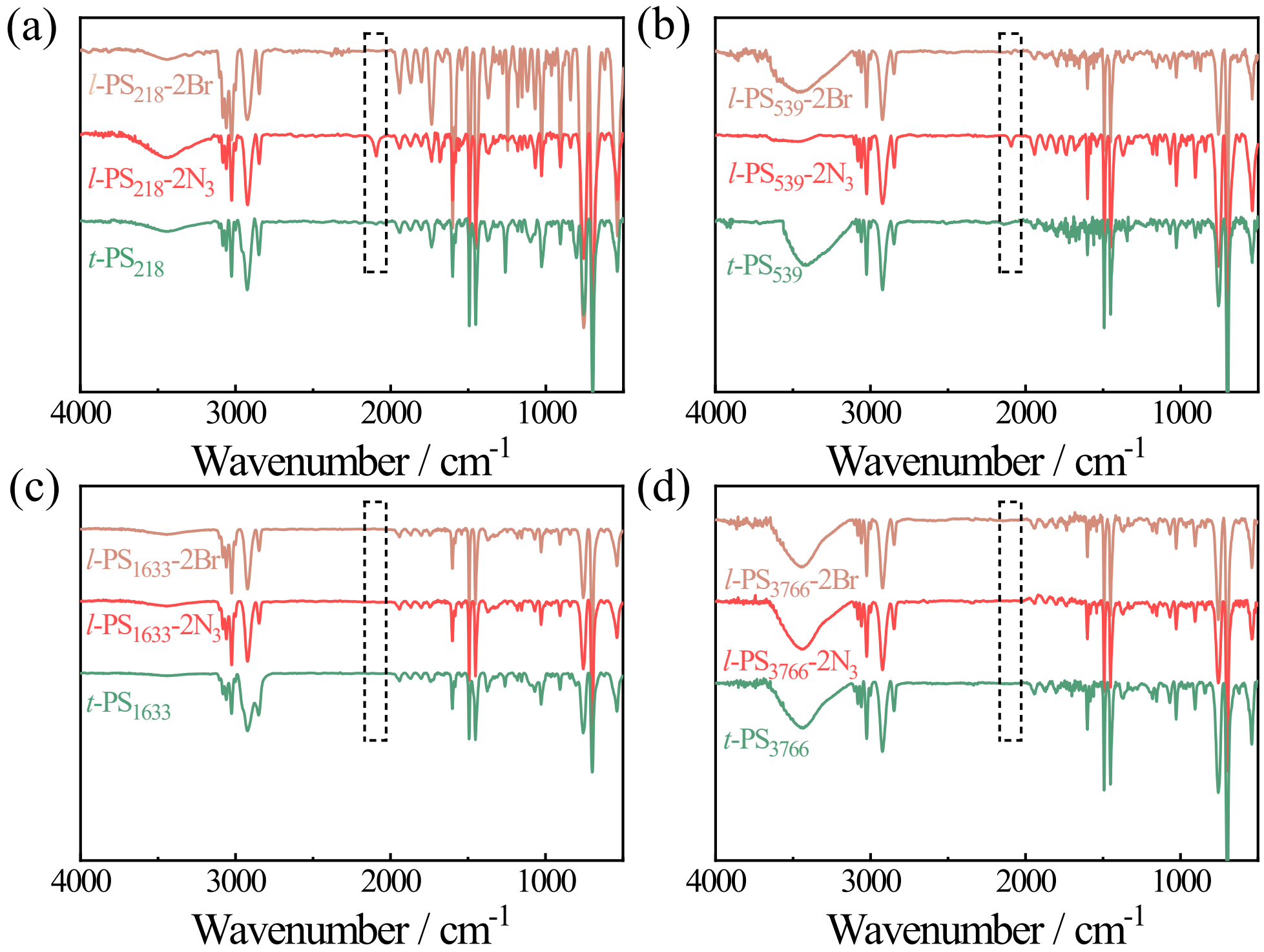

Figure S5. (a-d) FTIR spectra of l-PS$_n$-2Br, l-PS$_n$-2N$_3$ and t-PS$_n$ ($n \geq 218$) samples.

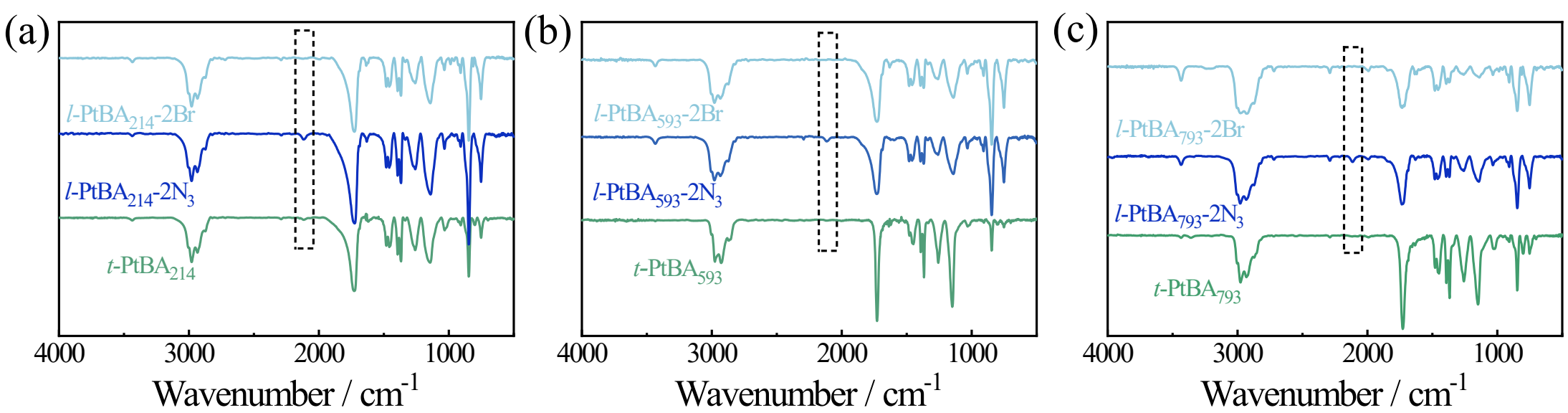

Figure S6. (a-c) FTIR spectra of l-PtBA$_n$-2Br, l-PtBA$_n$-2N$_3$ and t-PtBA$_n$ ($n \geq 214$) samples.

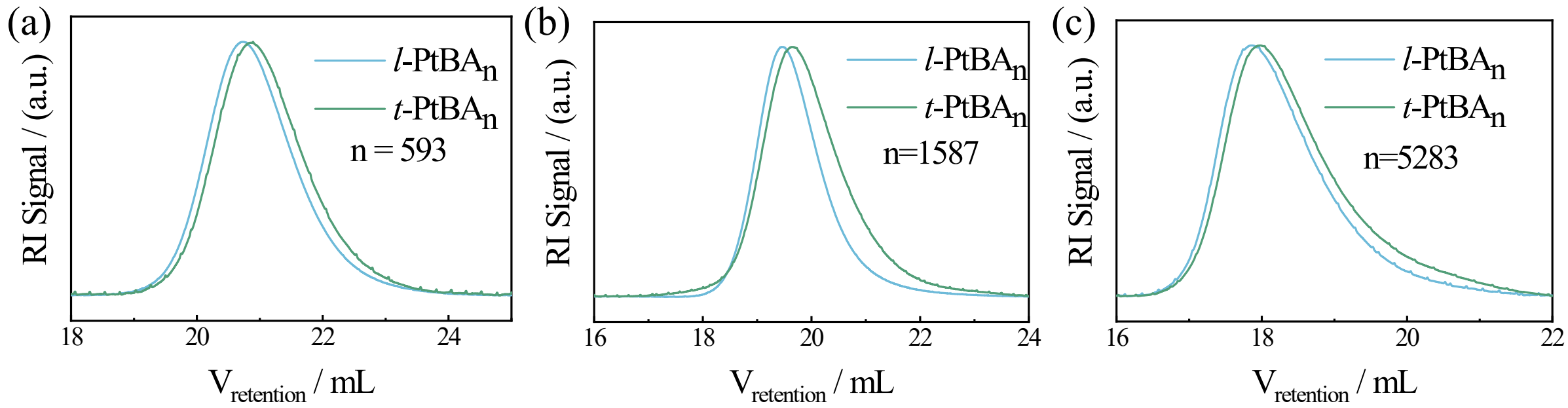

Figure S7. Conventional traces for the remaining *l*-$PtBA_n$ and their corresponding tadpole polymers (*t*-$PtBA_n$) using THF as the eluent, where traces (a), (b), and (c) correspond to polymers with a degree of polymerization (n) of 593, 1587, and 5283, respectively.

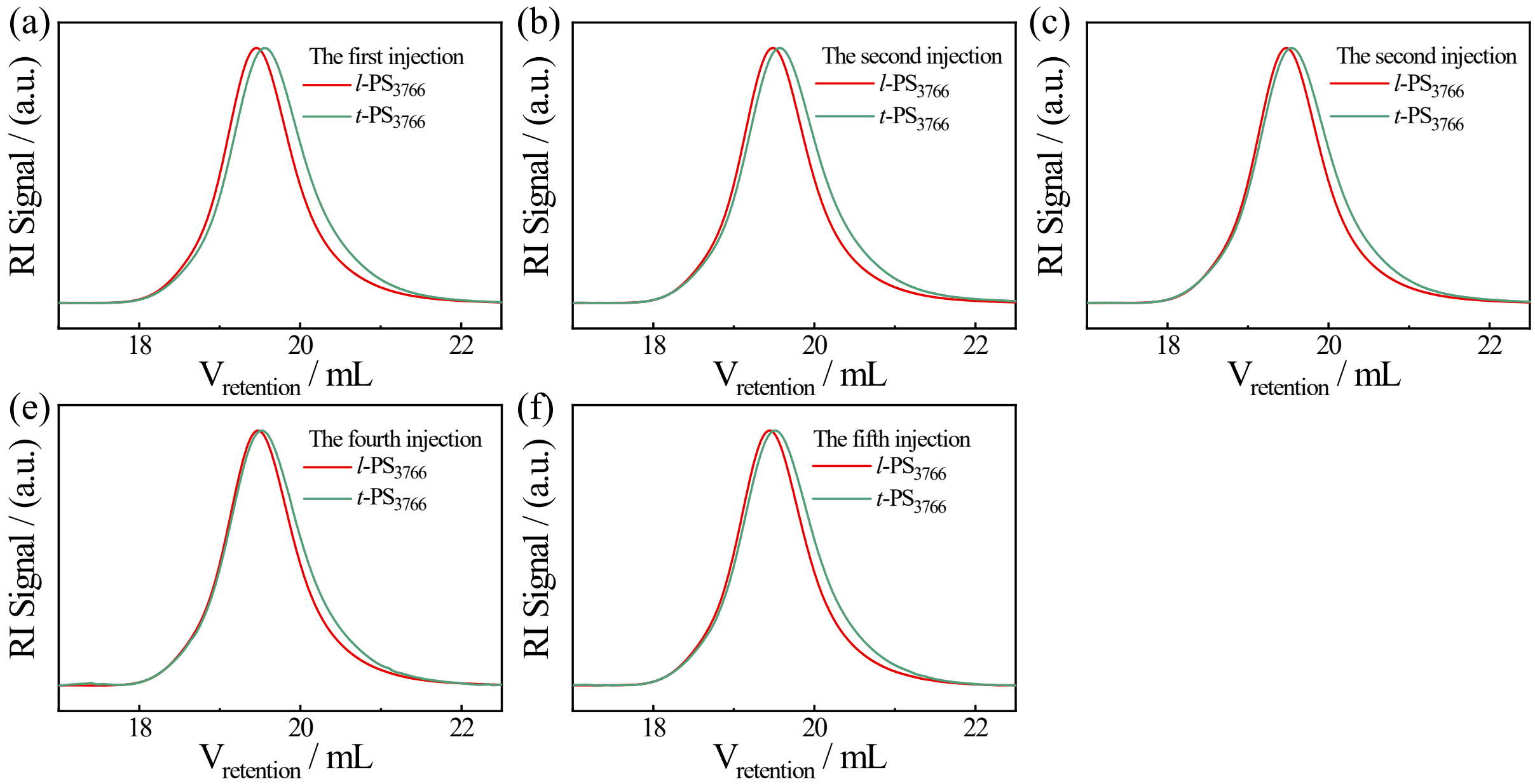

Figure S8. (a-f) Individual presentation of GPC elution curves from repeated cyclization reaction tests for *l*-PS_{3766} and *t*-PS_{3766}.

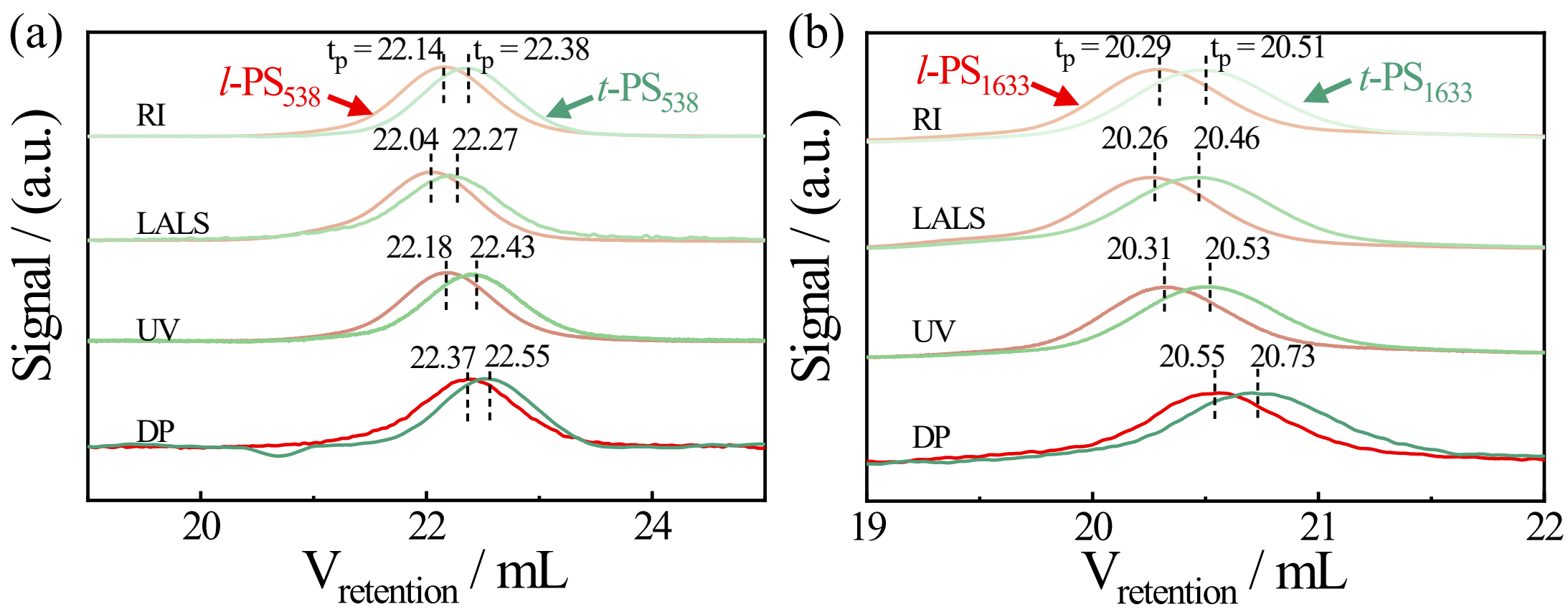

Figure S9. QD-GPC traces comparing the differential refractive index (RI), ultraviolet (UV), low-angle light scattering (LALS), and viscosity (DP) signals for l-PS$_n$ and t-PS$_n$, with degrees of polymerization (n) of (a) 538 and (b) 1633, respectively.

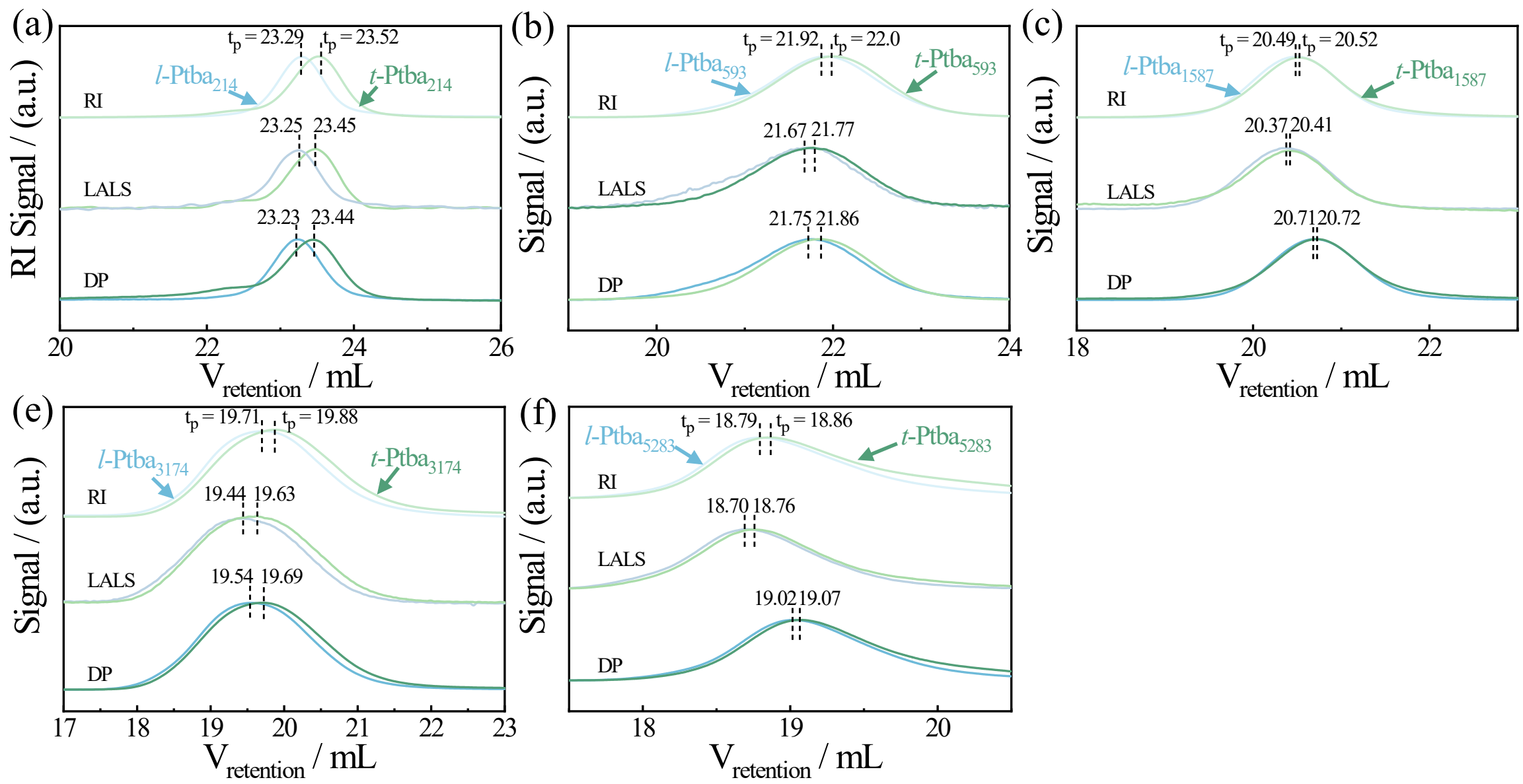

Figure S10. QD-GPC traces comparing of differential refractive index (RI), ultraviolet (UV), low angle light scattering (LALS), and viscosity (DP) signals for l-PtBA$_n$ and t-PtBA$_n$, with degrees of polymerization of (a) 214 , (b) 593, (c) 1587, (d) 3174 and (e) 5283, respectively.

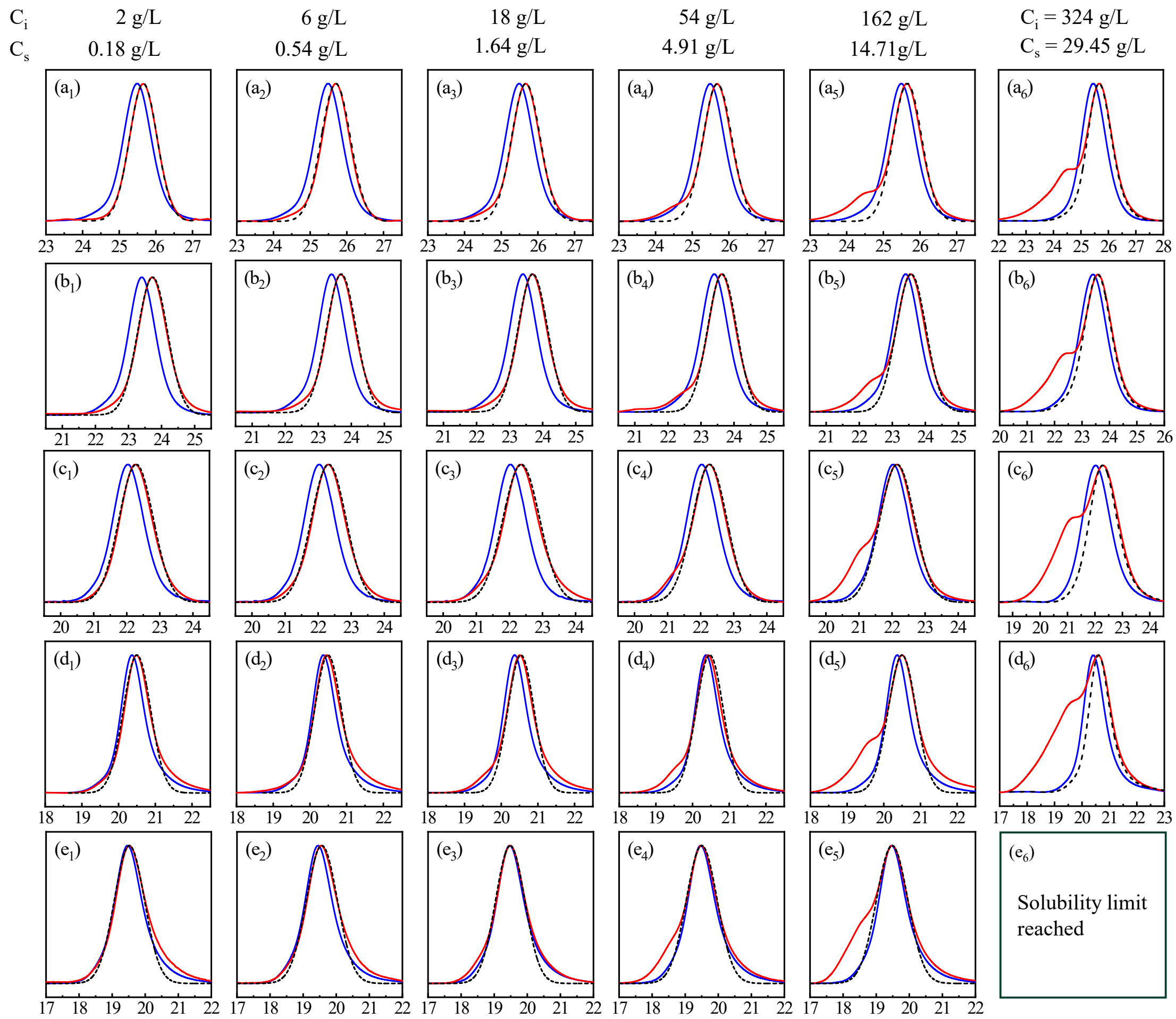

Figure S11. Conventional GPC curves of *t*-PS$_n$ obtained from *l*-PS$_n$ samples with different molecular mass under varying injection and solution concentrations (a) n = 56, (b) n = 218, (c) n = 538, (d) n = 1633, (e) n = 3766. The injection concentratio ngradient is C_i = 2, 6, 18, 54, 162, 324 g/L, and the solution concentration gradient is C_s= 0.18, 0.54, 1.64, 4.91, 14.71,29.45 g/L.

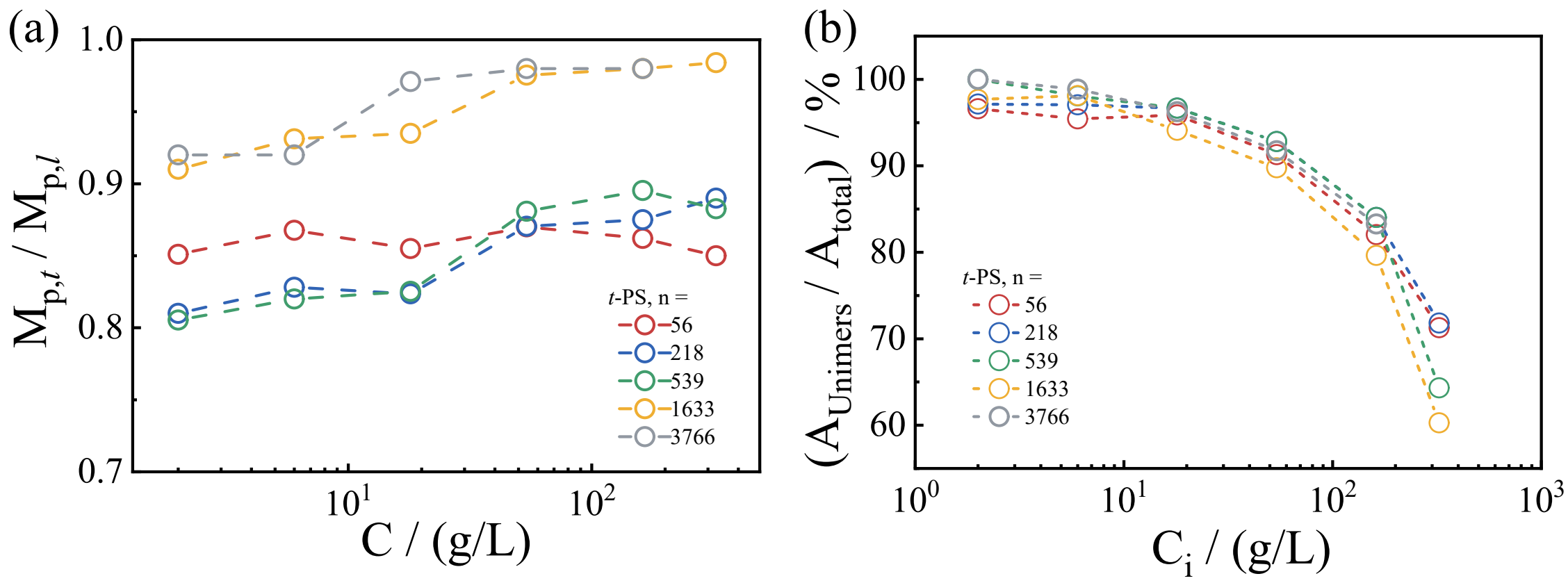

Figure S12. (a) Dependence of the peak apparent molecular mass ratio between tadpole-shaped polymers and linear precursors ($M_{rel,\ p,\ t}/M_{rel,\ p,\ l}$) on injection concentration (C_i) for samples with varying molecular weights. (b) Correlation between the unimer fraction (quantified via GPC peak deconvolution) of linear precursors (*l*-PS_n) and cyclized tadpole polymers (*t*-PS_n) and C_i.

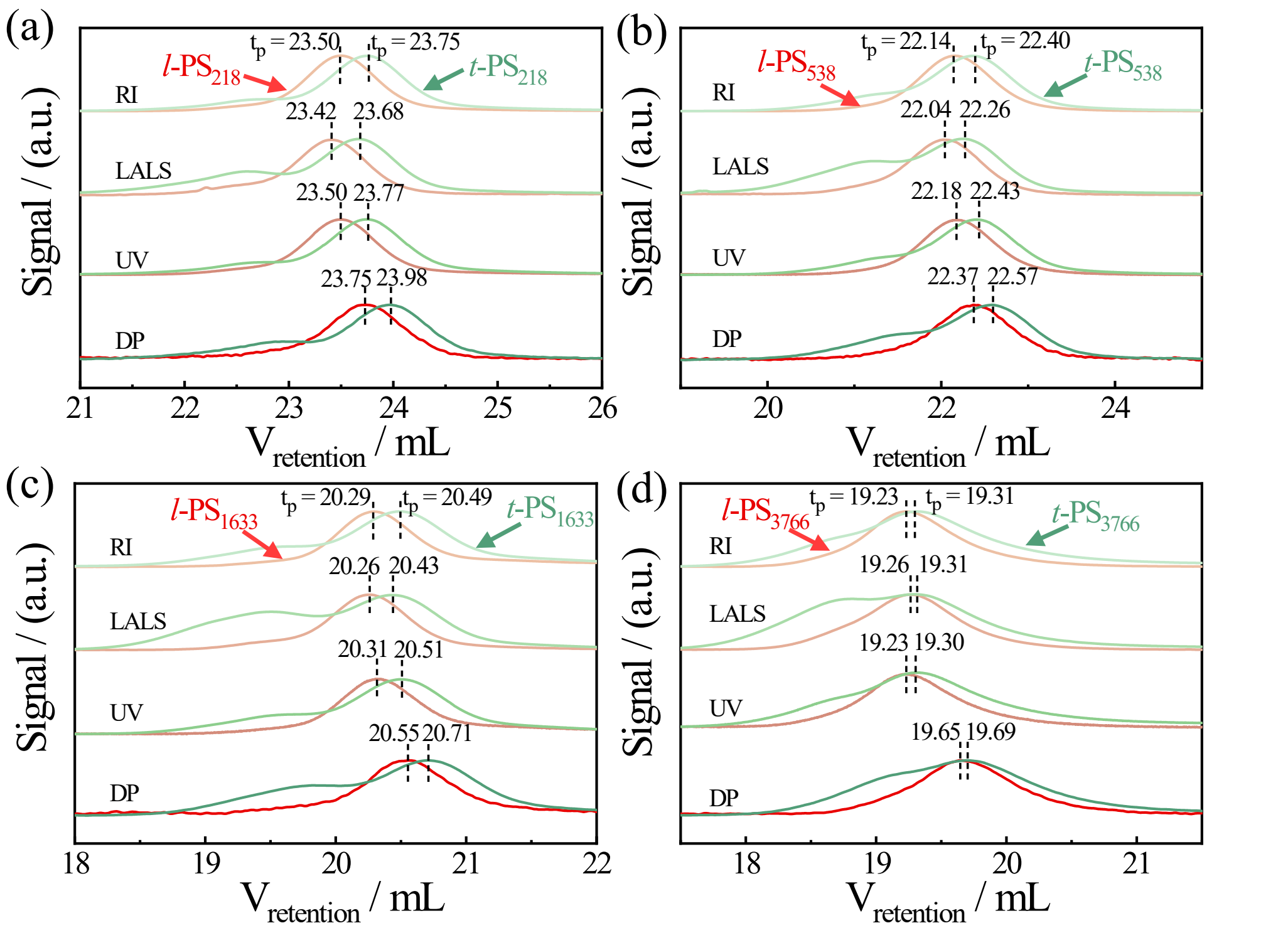

Figure S13. QD-GPC for the *l*-PS_n and *t*-PS_n with (a) n = 218, (b) n = 538, (c) n = 1633, and (d) n = 3766, under C_i = 162 g/L solution conditions, illustrating the four signals—differential refractive index, ultraviolet, light scattering, and viscosity.

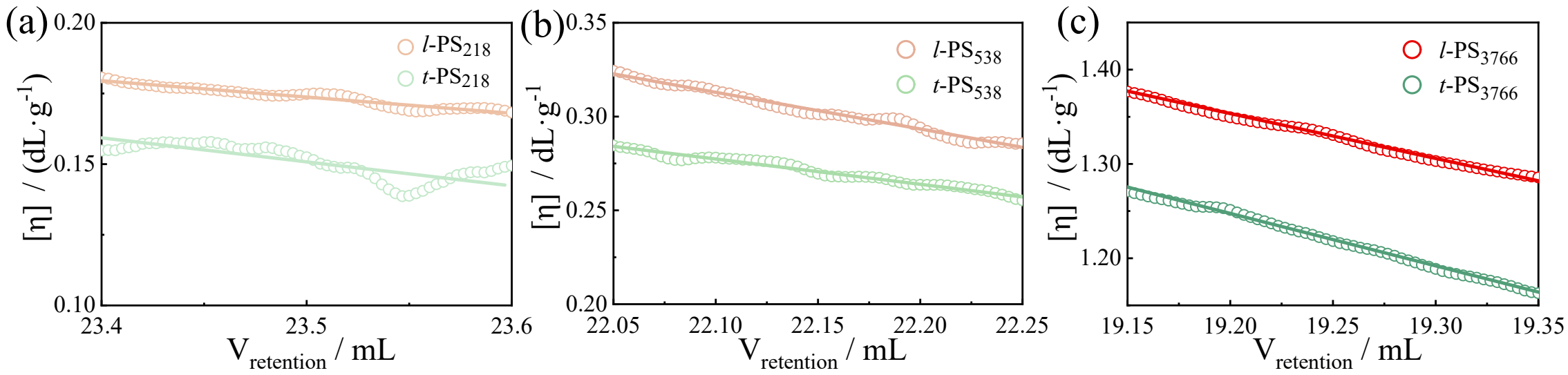

Figure S14. Intrinsic viscosity ([η]) vs. elution volume for *l*-PS$_n$ and *t*-PS$_n$, with degrees of polymerization of (a) 218, (b) 538 and (c) 3766, respectively, measured by QD-GPC.

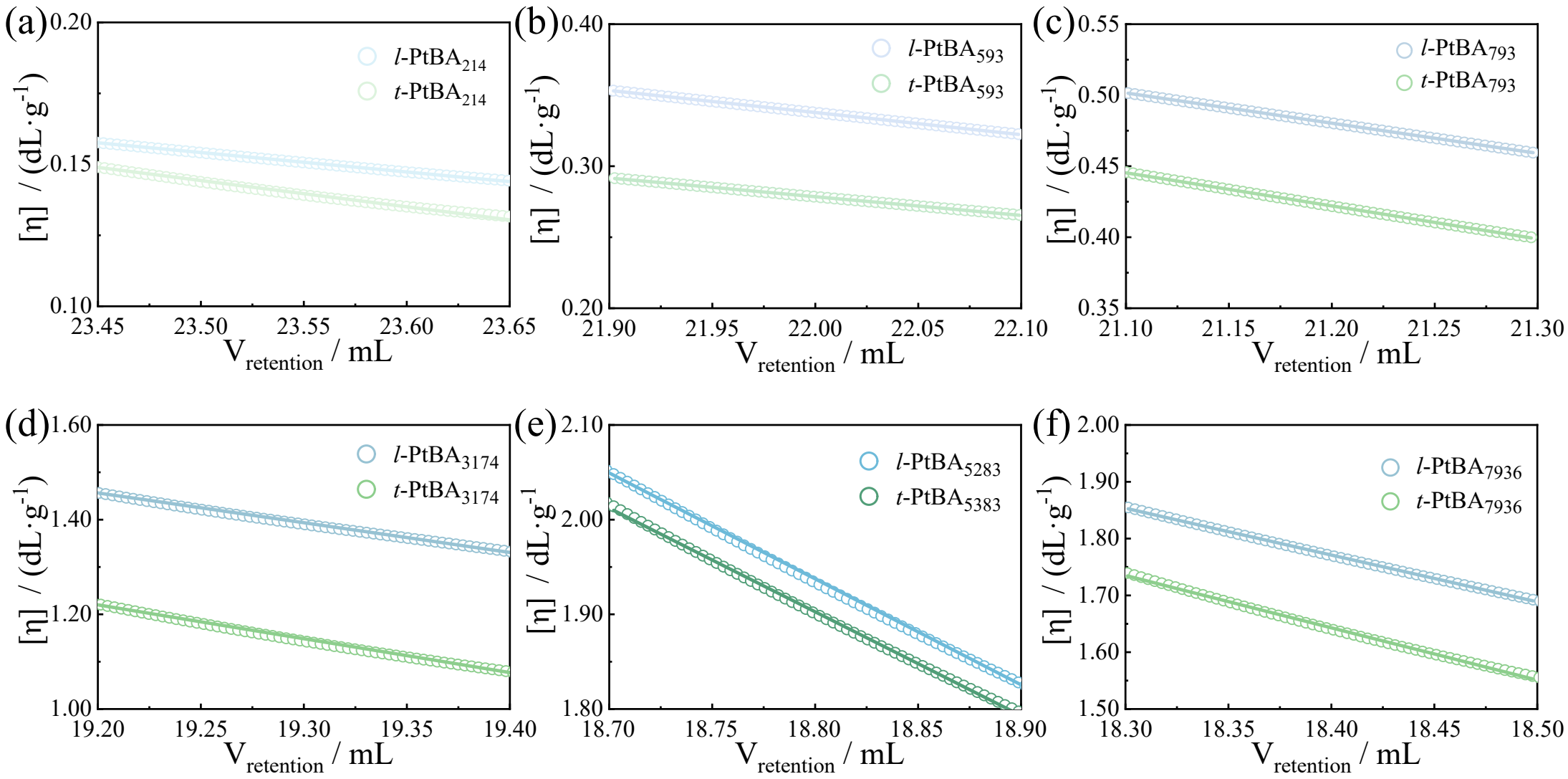

Figure S15. Intrinsic viscosity ([η]) vs. elution volume for *l*-PtBA$_n$ and *t*-PtBA$_n$, with degrees of polymerization of (a) n = 214, (b) n = 593, (c) n = 793, (d) n = 3174, (e) n = 5283 and (f) n = 7936, respectively, measured by QD-GPC.

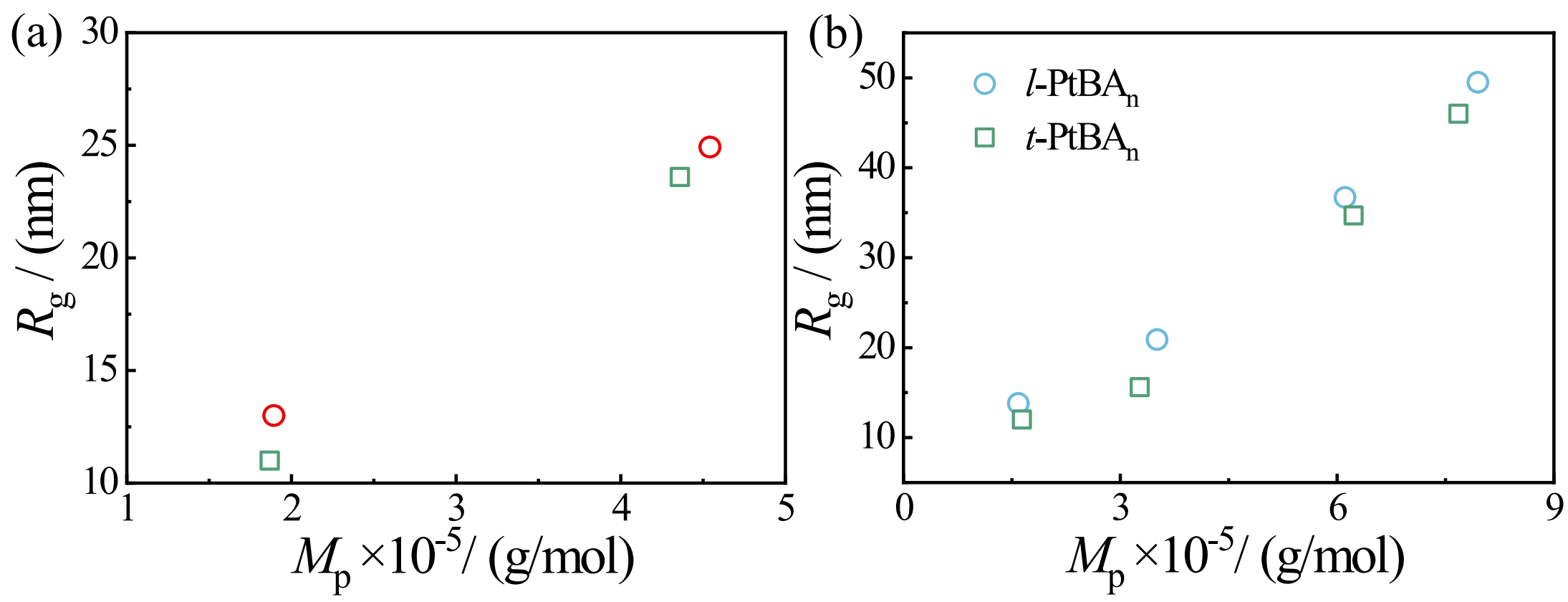

Figure S16. Analysis of the radius of gyration versus absolute molecular mass at the peak positions in QD-GPC for (a) *l*-PS$_n$/*t*-PS$_n$ and (b) *l*-PtBA$_n$/*t*-PtBA$_n$. The data represent polymers with degrees of polymerization of 1633, 3766 for PS, and 1587, 3174, 5283, 7936 for PtBA. Samples with insufficient molecular dimensions (n ≤ 538 for PS; n ≤ 793 for PtBA) were excluded due to poor signal-to-noise ratio.

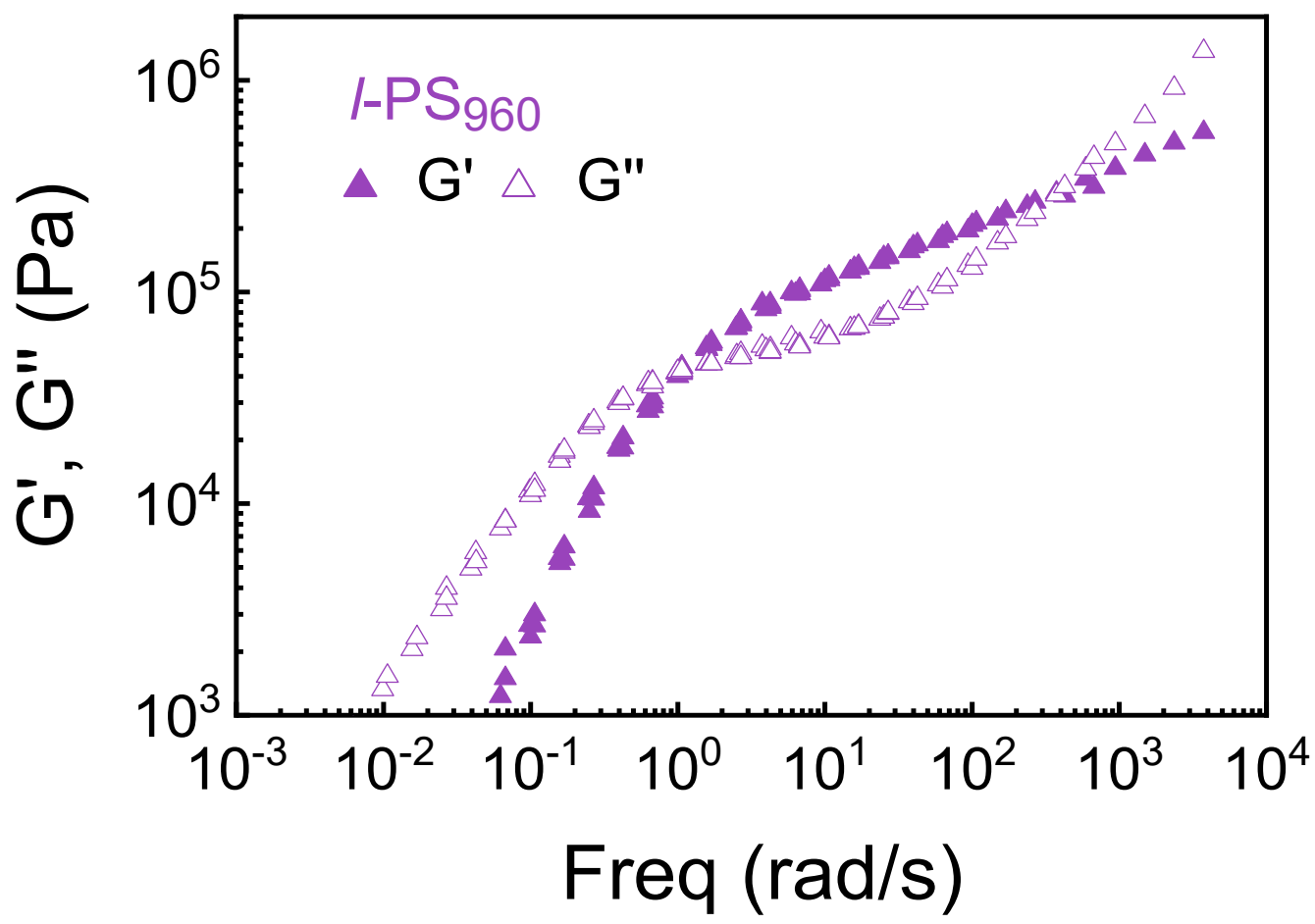

Figure S17. Frequency dependence of the storage (G′) and loss (G″) moduli for the linear *l*-PS$_{960}$ control. Frequency sweeps collected at 70-130 °C were horizontally shifted to construct a time–temperature superposition (TTS) master curve.

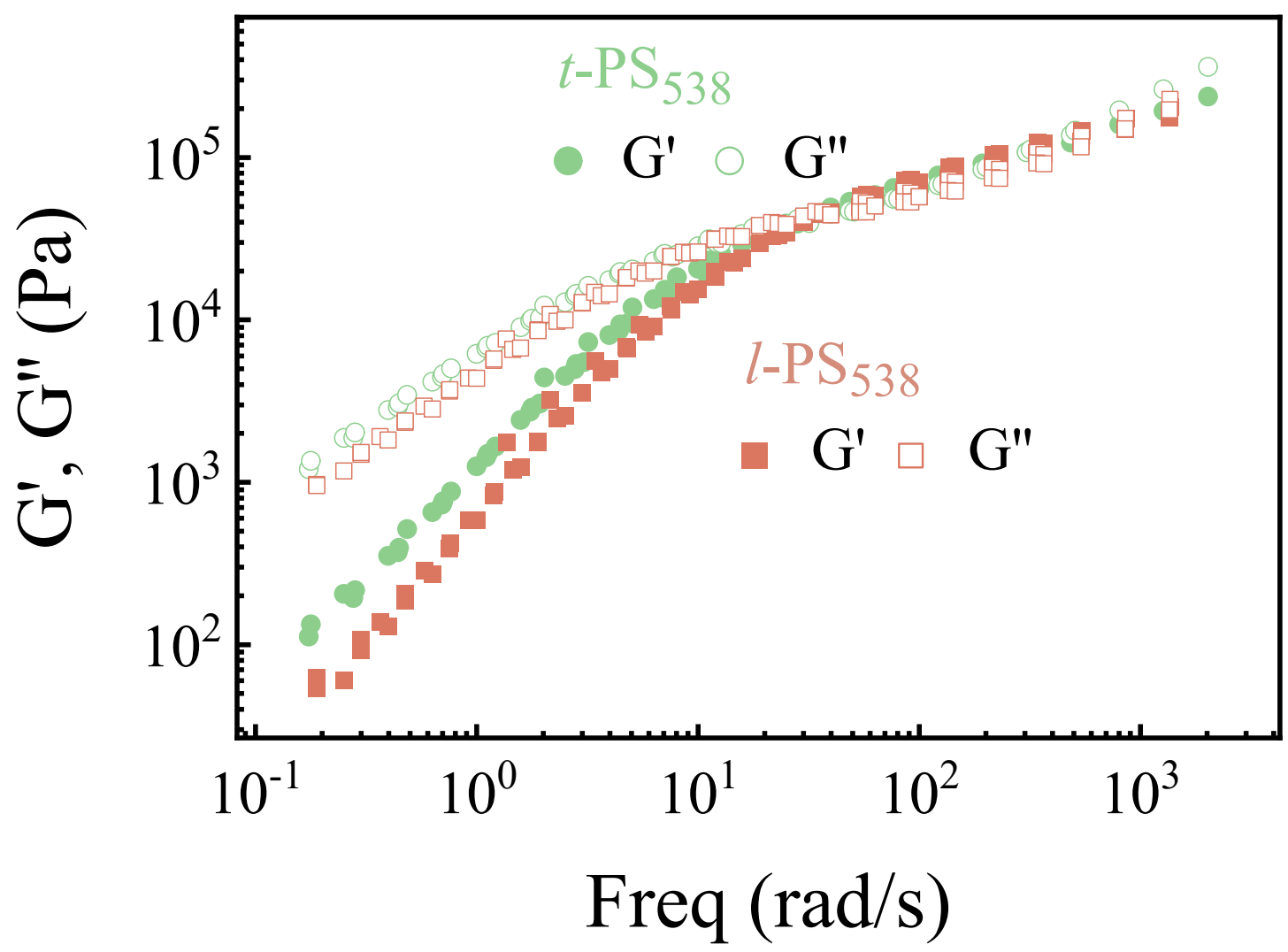

Figure S18. Frequency dependence of the storage (G′) and loss (G″) moduli for *l*-PS$_{538}$ and *t*-PS$_{538}$. Frequency sweeps collected at 70-130 °C were horizontally shifted to construct time–temperature superposition master curves.

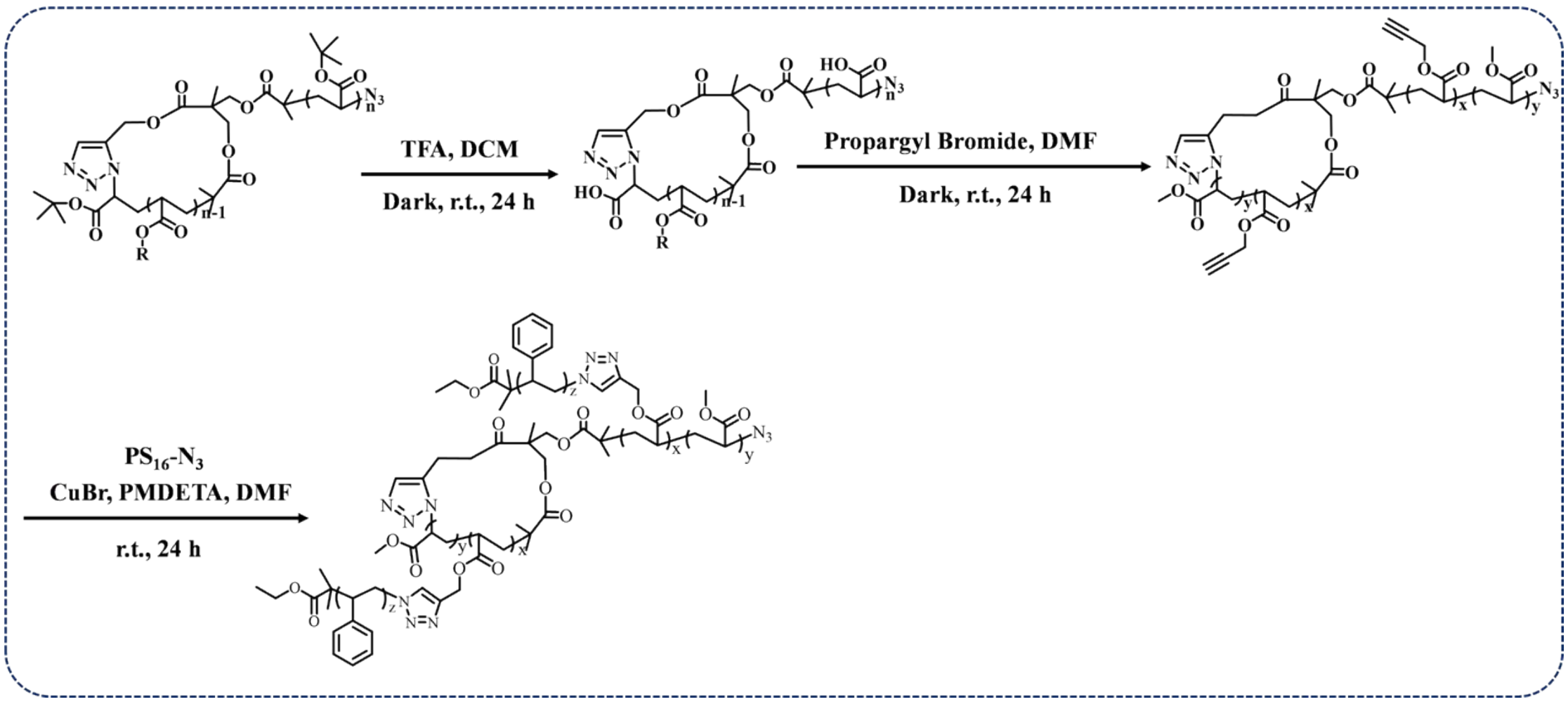

Figure S19. Synthesis route of *t*-PPA_{1587}-g-PS_{16}.

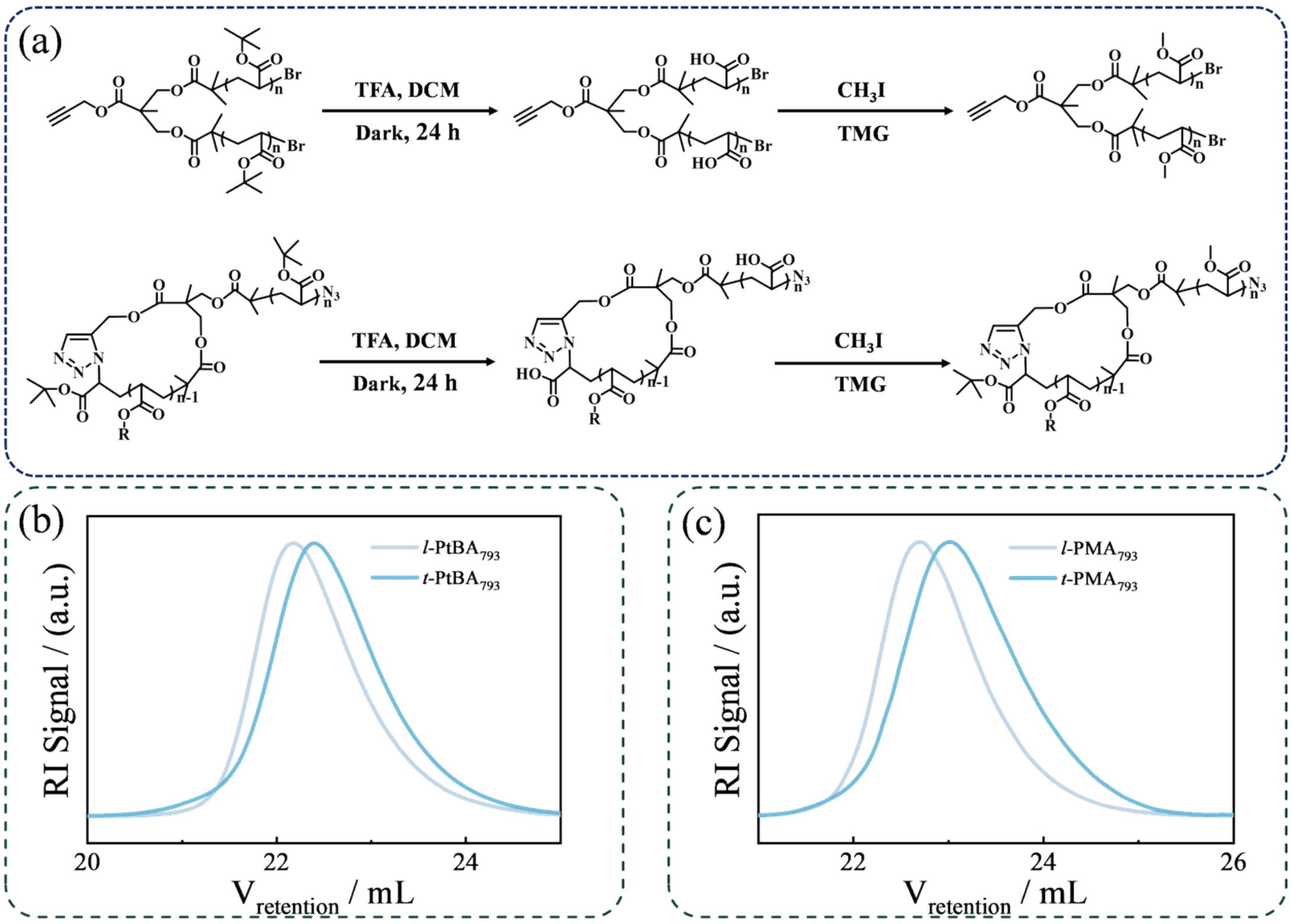

Figure S20. (a) Synthetic route from poly(tert-butyl acrylate) to poly(methyl acrylate). (b) Conventional curves for the remaining *l*-$PtBA_{793}$ and their corresponding tadpole polymers using THF as the eluent. (c) Conventional curves for the remaining *l*-PMA_{793} and their corresponding tadpole using THF as the eluent.

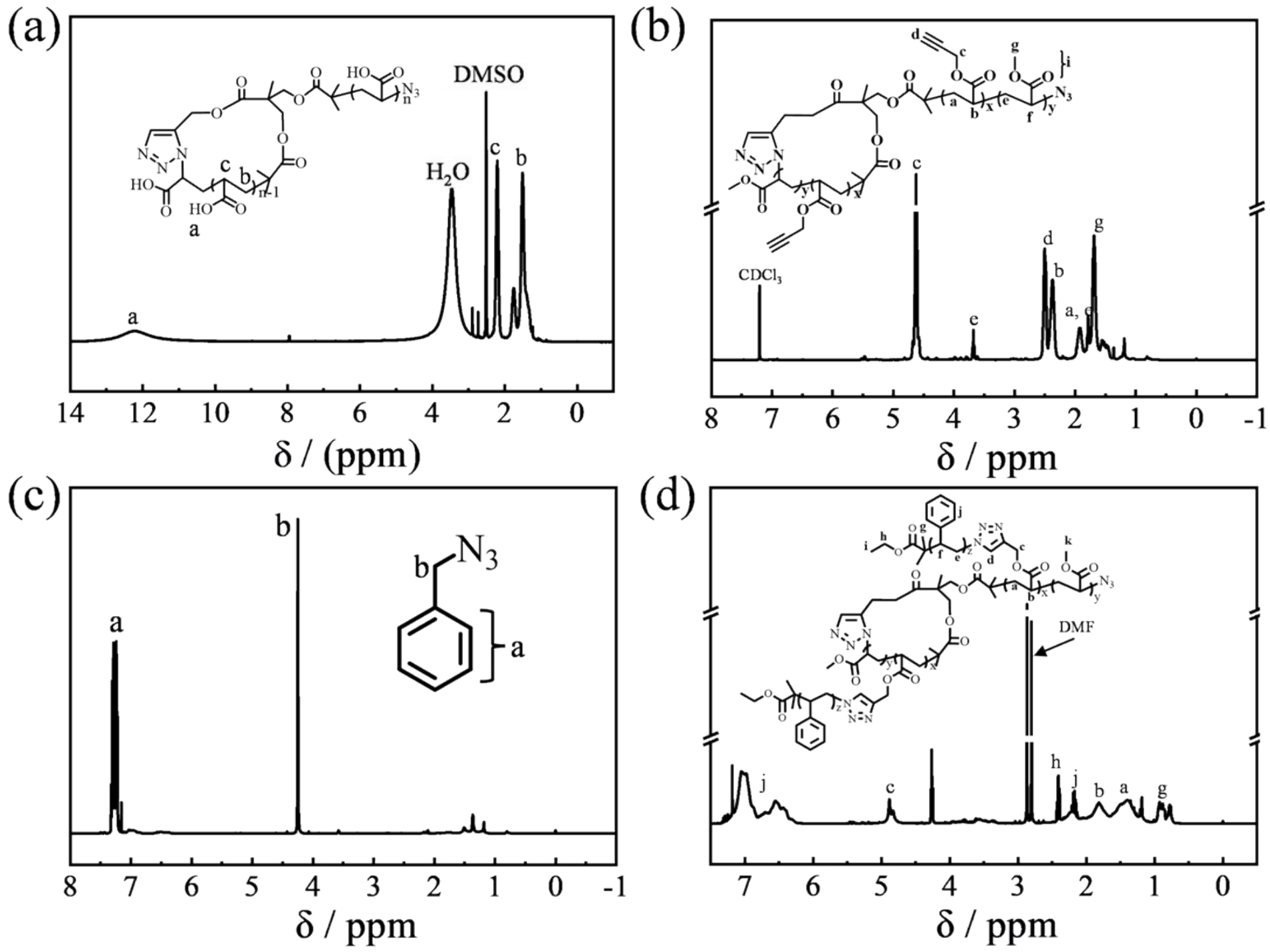

Figure S21. ^{1}H NMR spectra of (a) *t*-PAA$_{1587}$ in DMSO-d$_6$, (b) *t*-PPA$_{1587}$ in CDCl$_3$, (c) benzyl azide in CDCl$_3$, and (d)*t*-PPA$_{1587}$-g-PS$_{16}$ in CDCl$_3$.

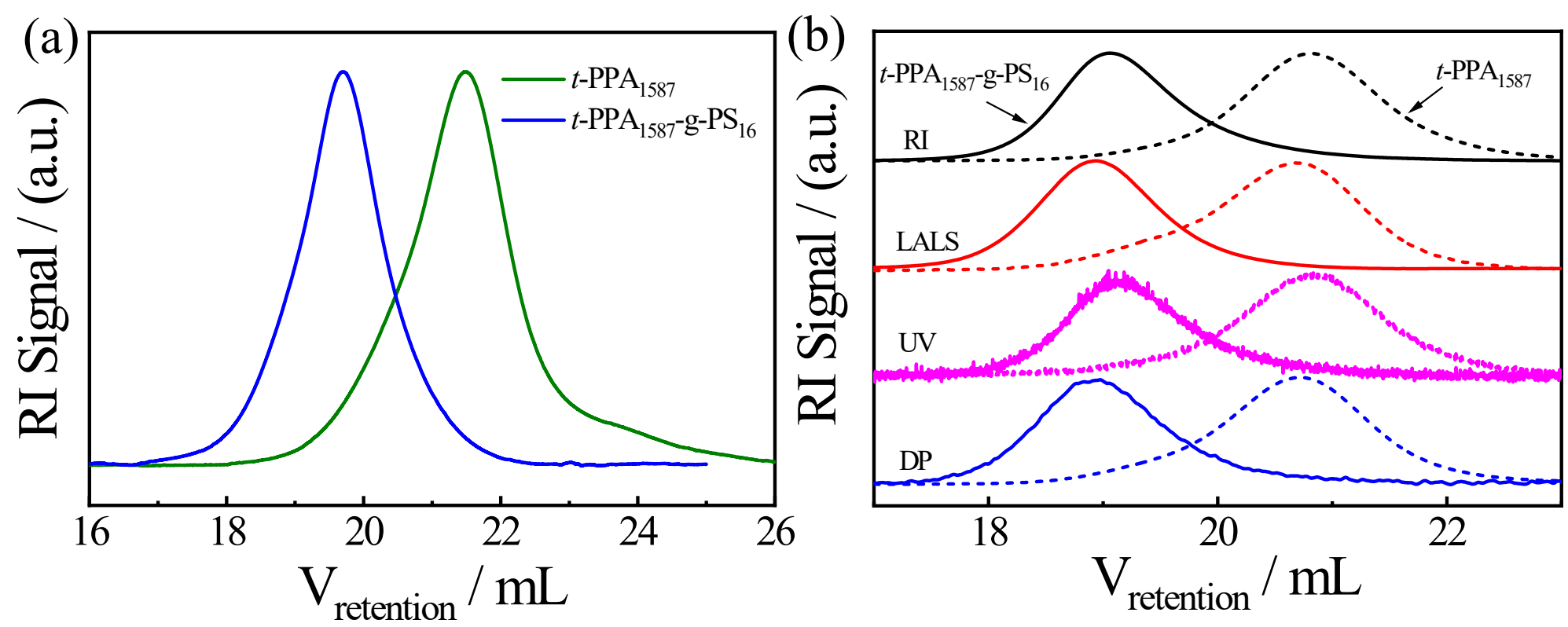

Figure S22. (a) Conventional GPC curves and (b) QD-GPC traces comparing of differential refractive index (RI), ultraviolet (UV), low angle light scattering (LALS), and viscosity (DP) signals for the *t*-PPA$_{1587}$ and *t*-PPA$_{1587}$-g-PS$_{16}$.

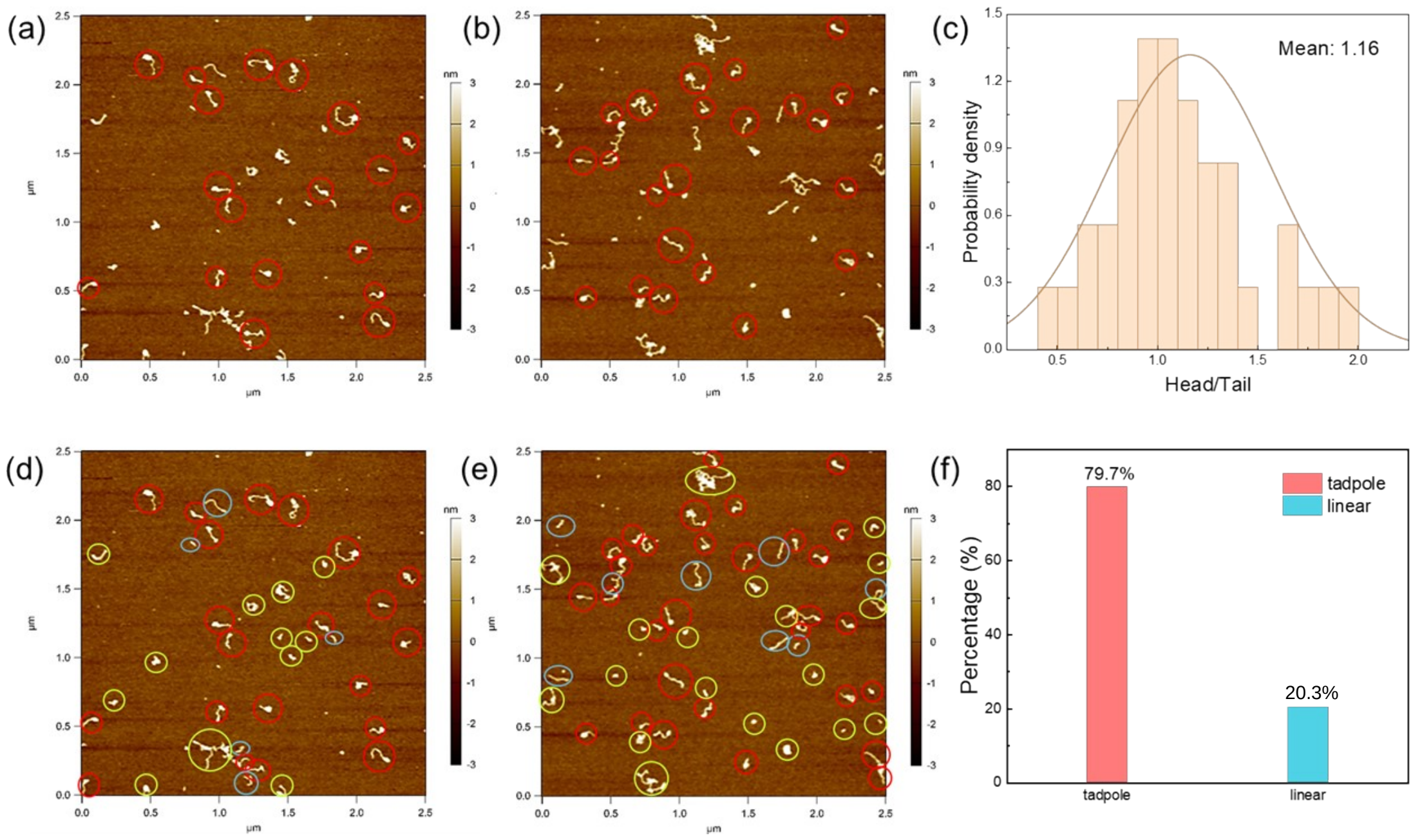

Figure S23. AFM-based statistical analysis of the head-to-tail geometry and morphology-based cyclization efficiency of *t*-PtBA$_{1587}$. (a-c) Contour-length analysis of individual tadpole-like molecules: (a-b) representative AFM phase images with backbone contour tracing and (c) the head-to-tail contour-length ratio distribution from 38 molecules (mean ≈ 1.16:1). (d-f) Morphology-based evaluation of cyclization efficiency: (d-e) representative AFM phase images used for classification and (f) population fractions after ambiguous structures were excluded, giving an estimated tadpole fraction of 79.7%. Red circles indicate tadpole-like molecules, blue circles indicate linear precursors, and yellow circles indicate ambiguous structures excluded from the statistical analysis.

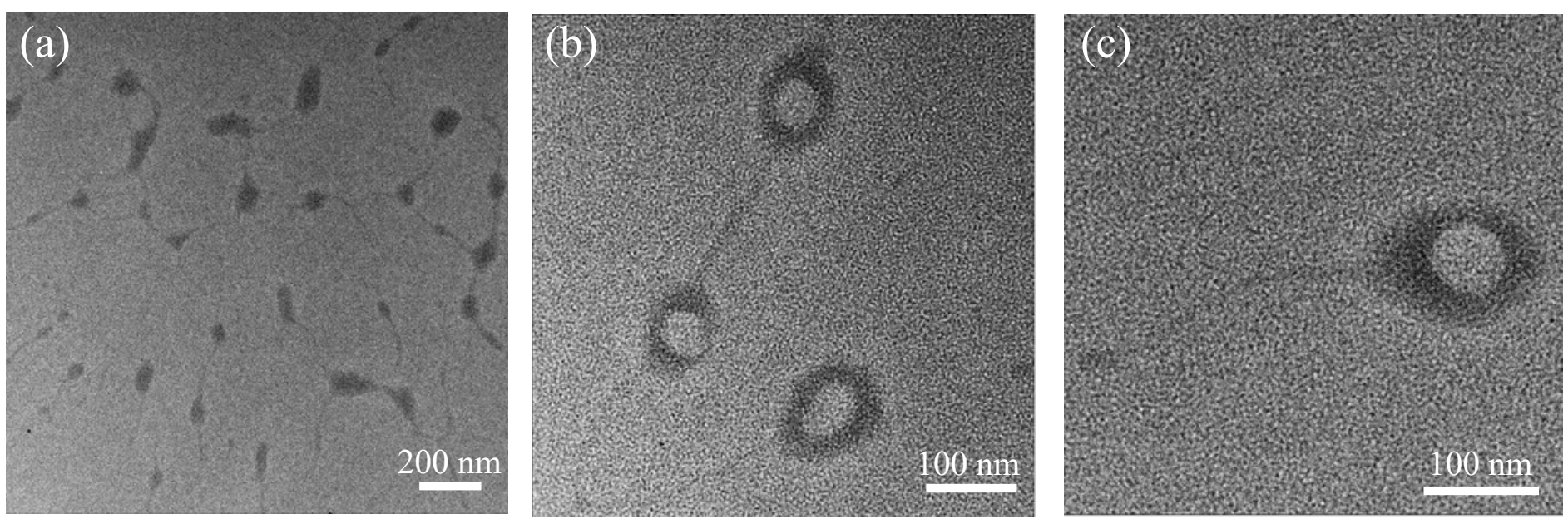

Figure S24. (a-c) TEM images of *t*-PPA$_{1587}$-graft-PS$_{16}$ deposited on a carbon-coated copper grid.

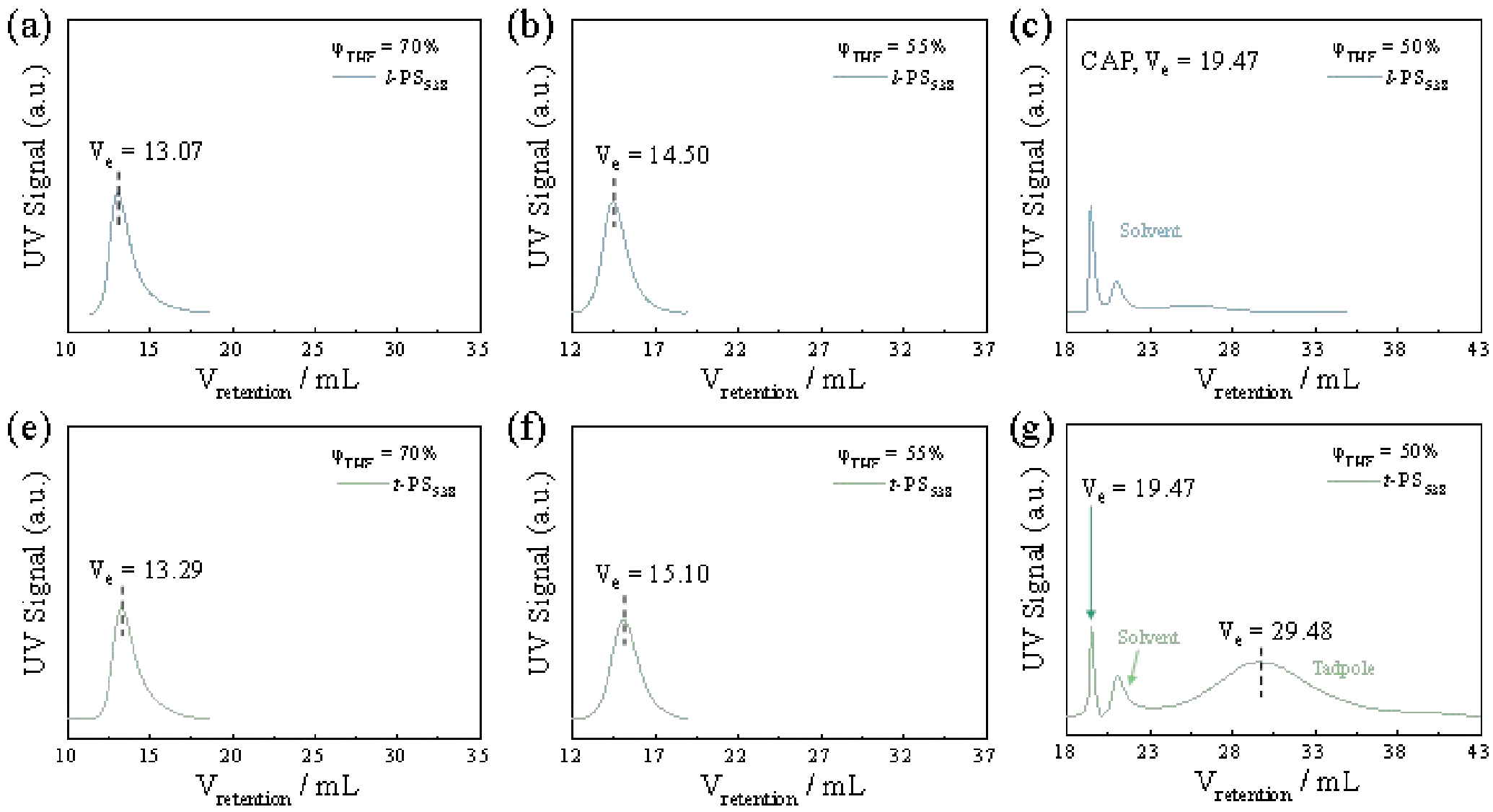

Figure S25. Critical adsorption behavior of *l*-PS_{538} (a–c) and *t*-PS_{538} with triazole-ring (d–f) in LCCC: evolution of chromatograms with decreasing φ_{THF}.

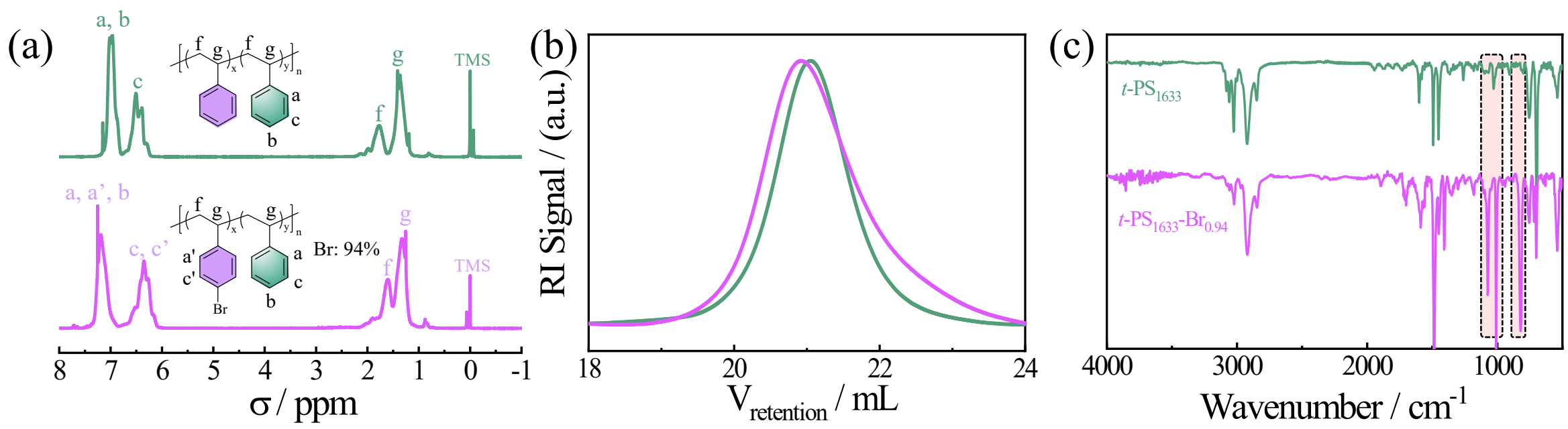

Figure S26. (a) ^{1}H NMR spectra, (b) GPC, and (c) FTIR spectra of *t*-PS_{1633} and *t*-PS_{1633}-$Br_{0.94}$.

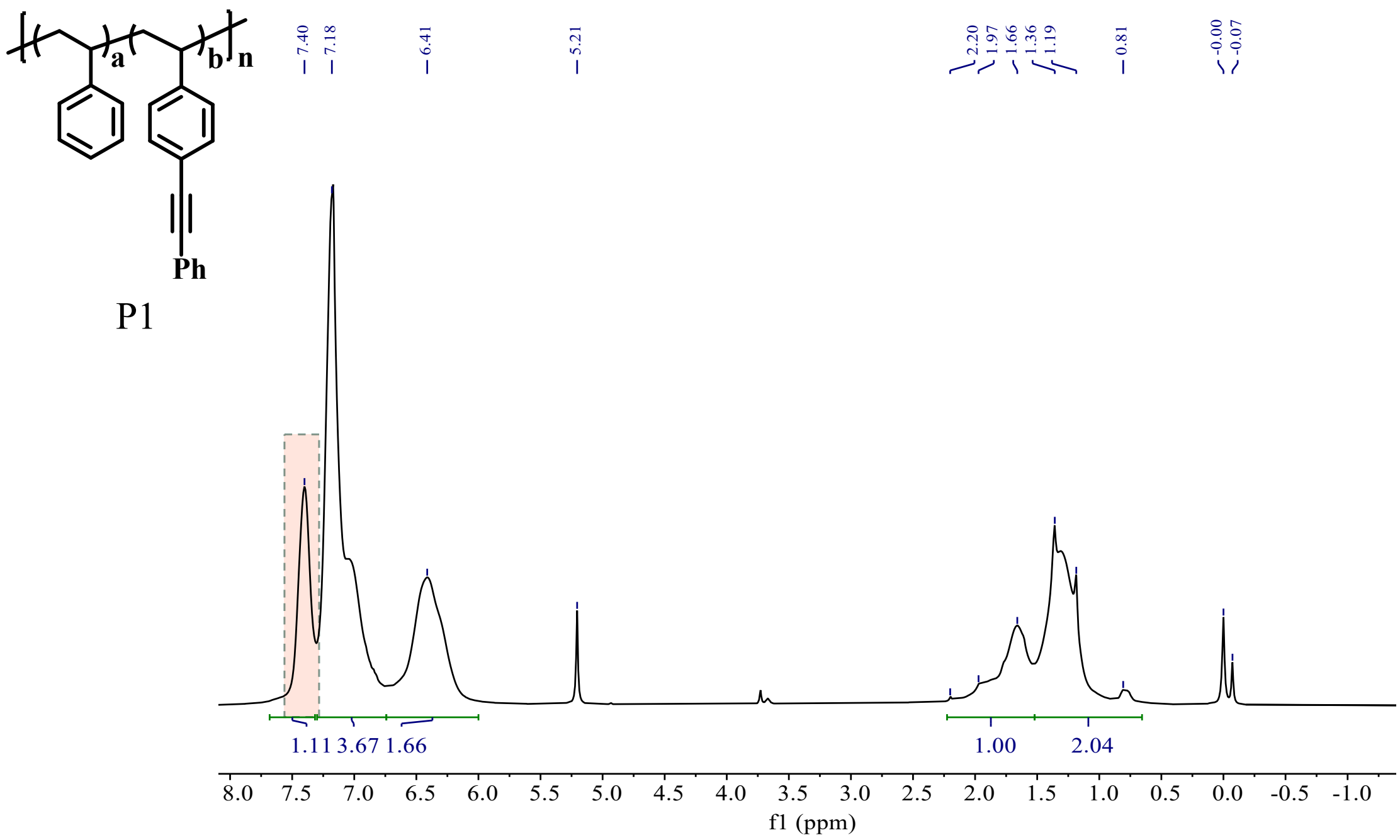

Figure S27. ^{1}H NMR (500 MHz, $CDCl_3$) spectrum of **P1**.

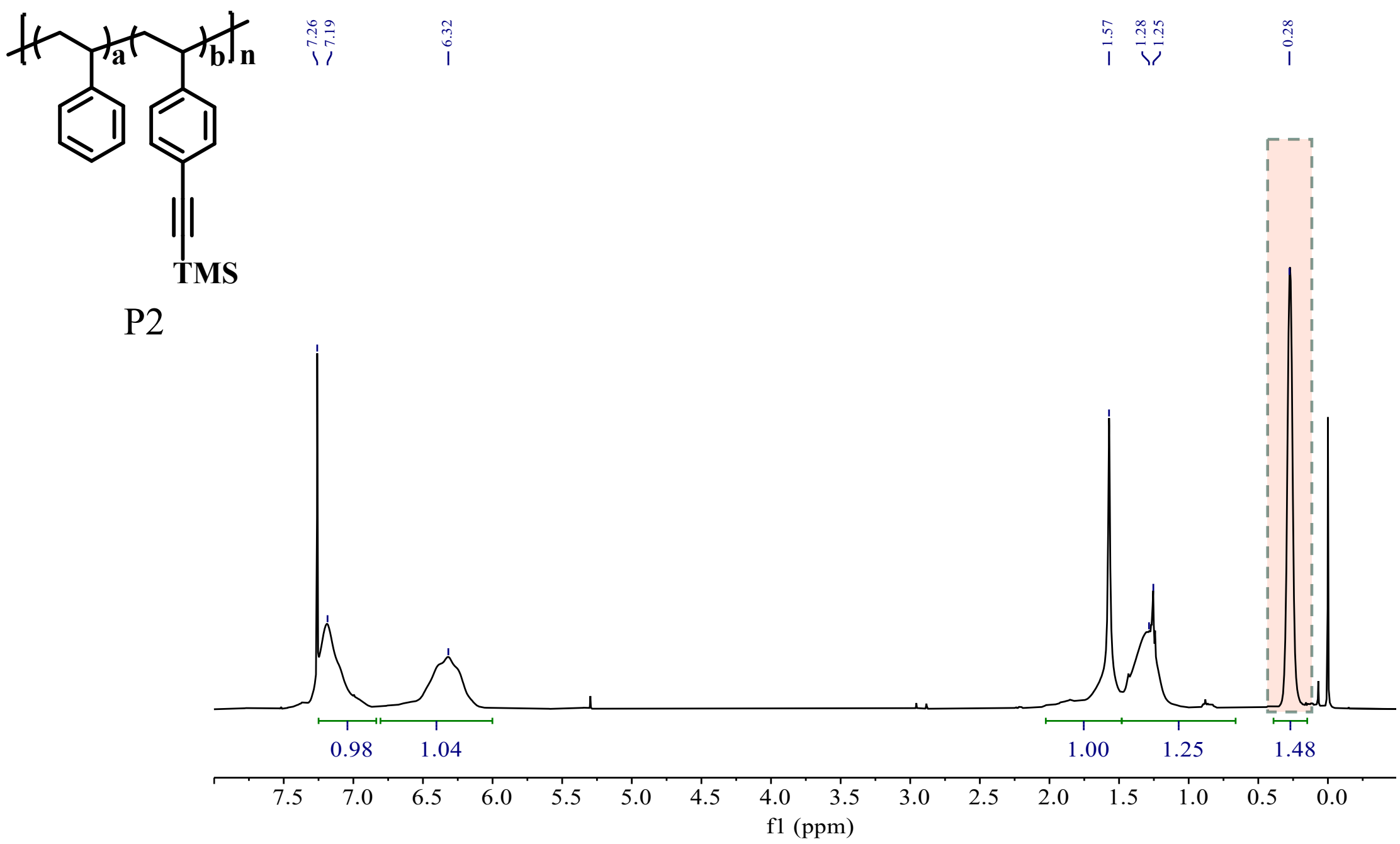

Figure S28. ^{1}H NMR (500 MHz, $CDCl_3$) spectrum of **P2**.

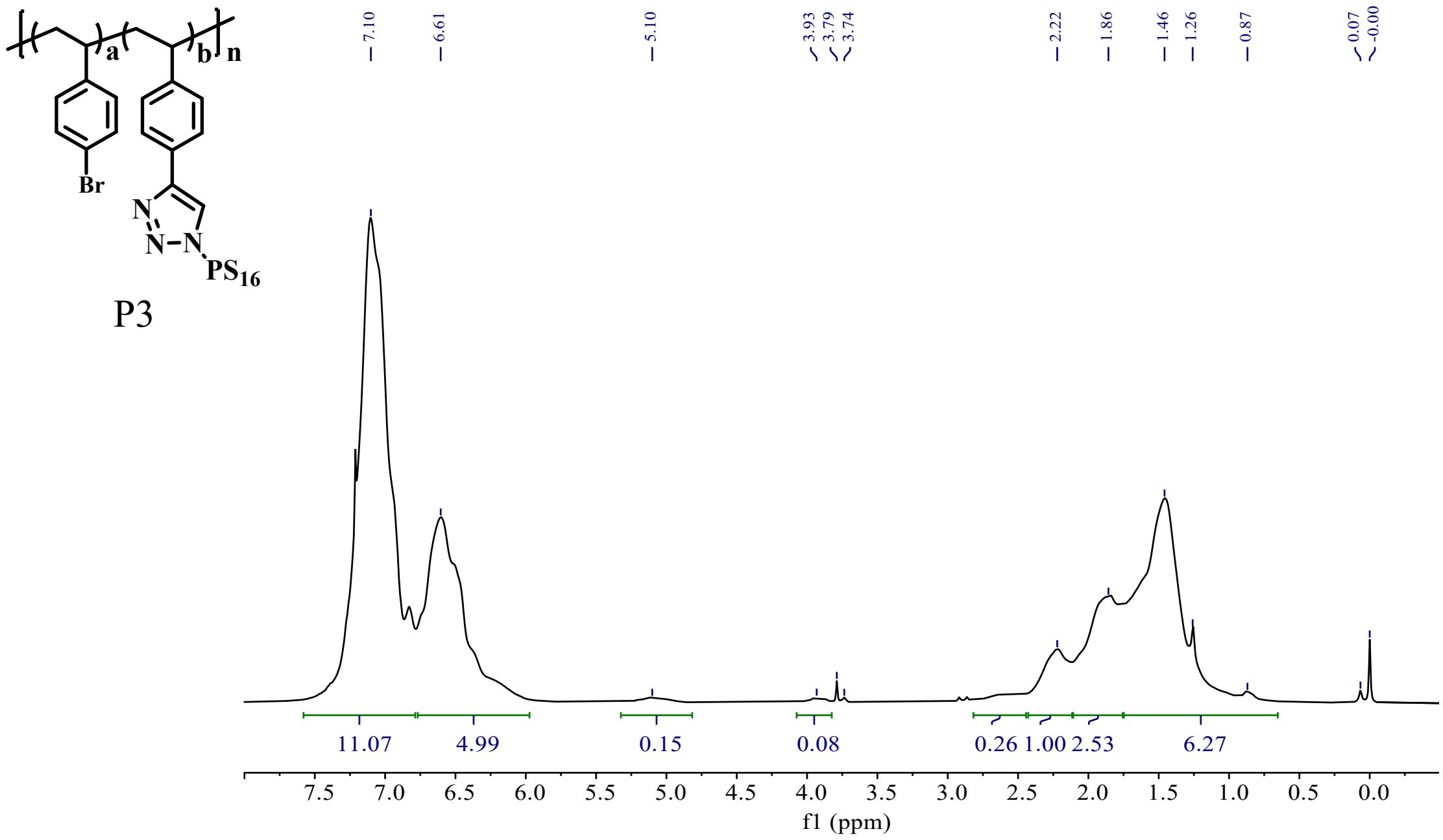

Figure S29. ^{1}H NMR (500 MHz, $CDCl_3$) spectrum of **P3**.

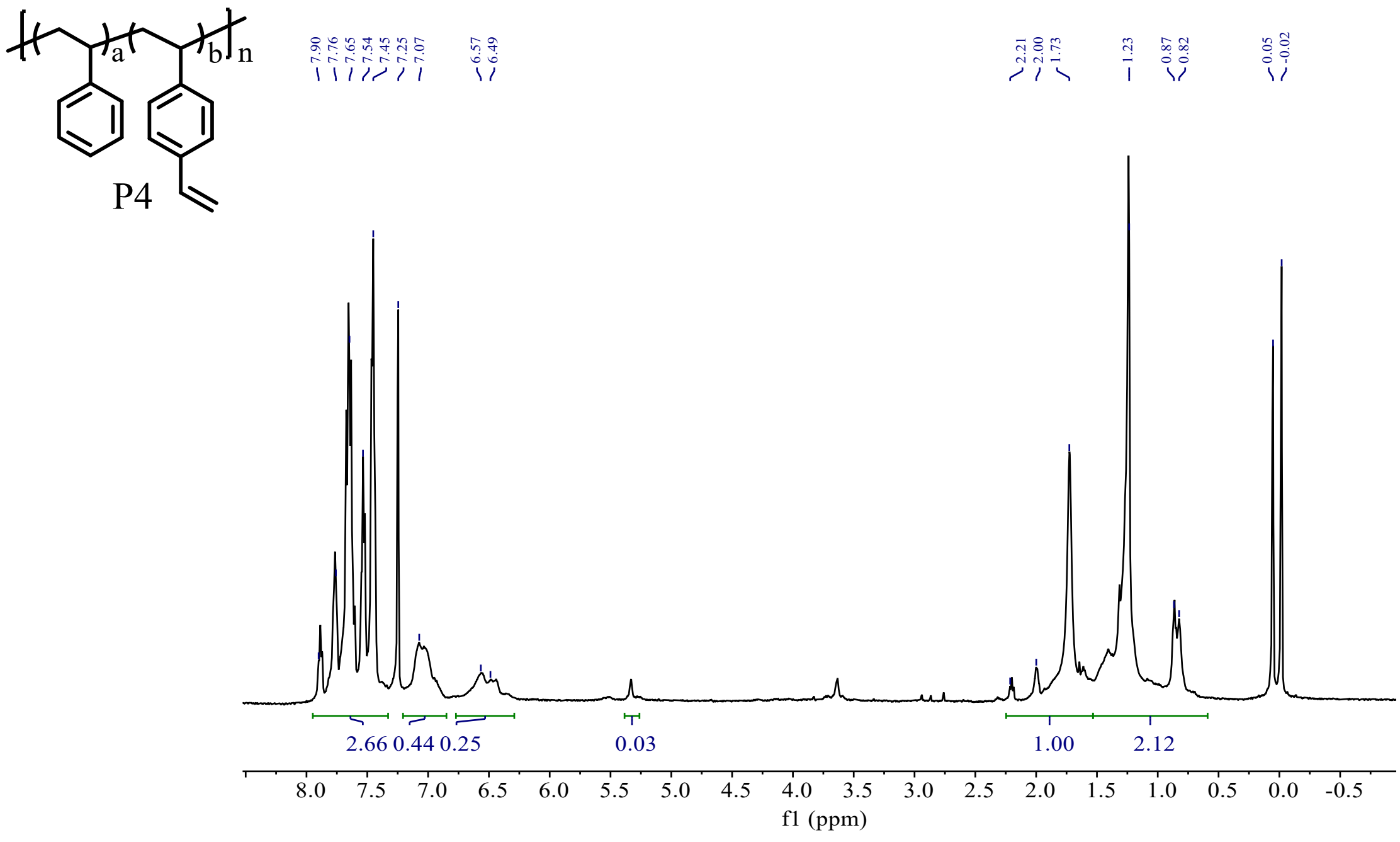

Figure S30. ^{1}H NMR (500 MHz, $CDCl_3$) spectrum of **P4**.

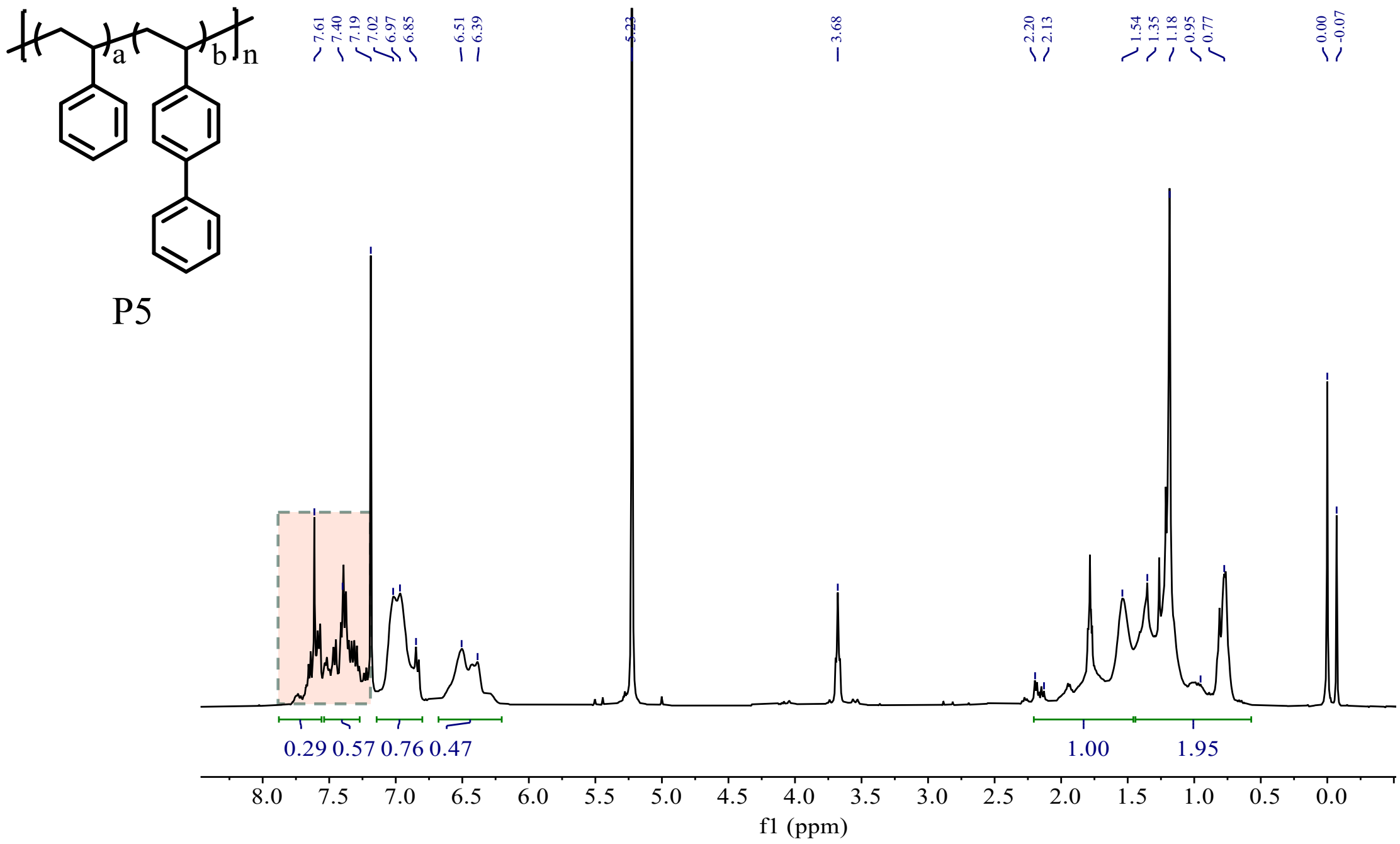

Figure S31. ^{1}H NMR (500 MHz, $CDCl_3$) spectrum of **P5**.

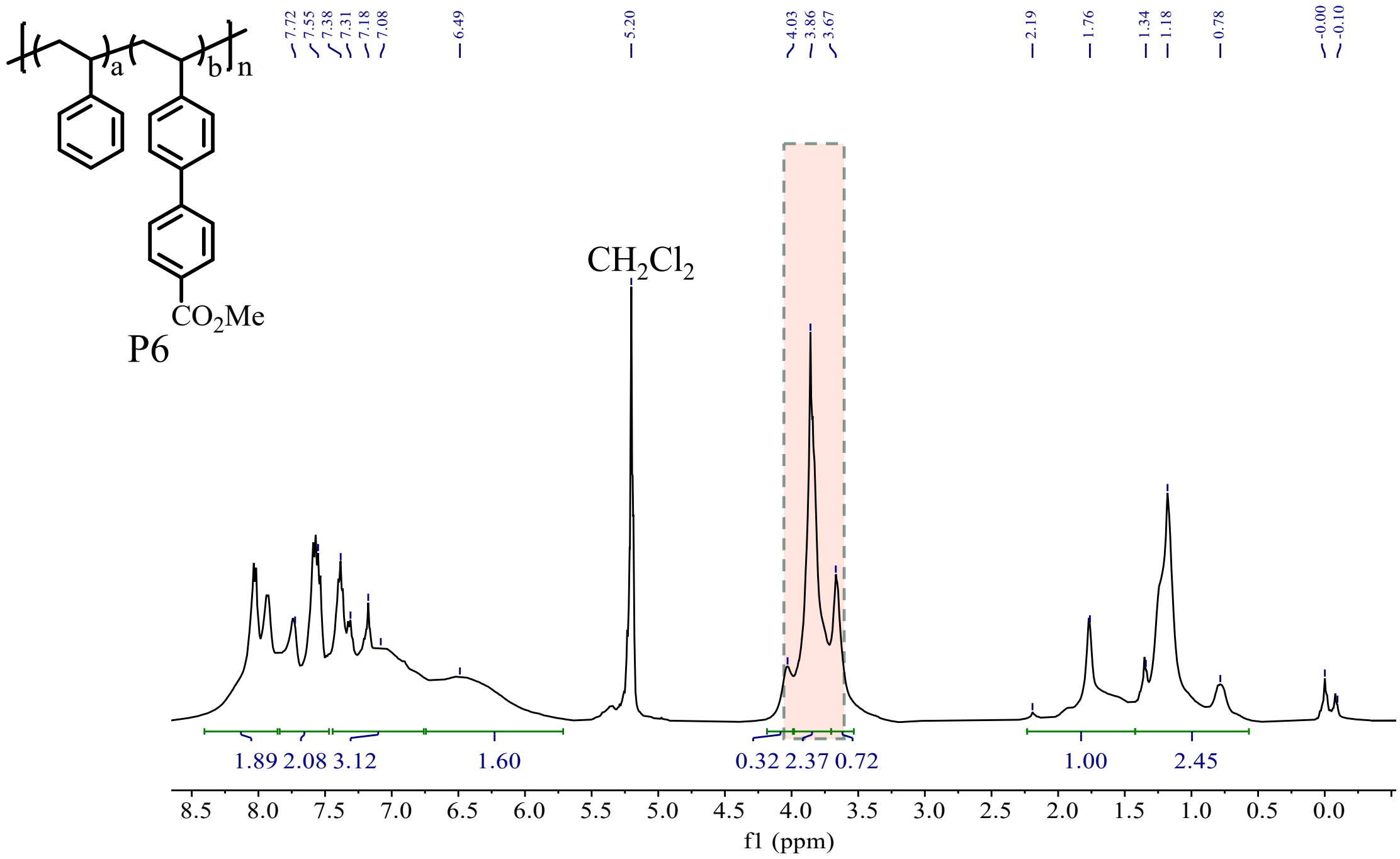

Figure S32. ^{1}H NMR (500 MHz, $CDCl_3$) spectrum of **P6**.

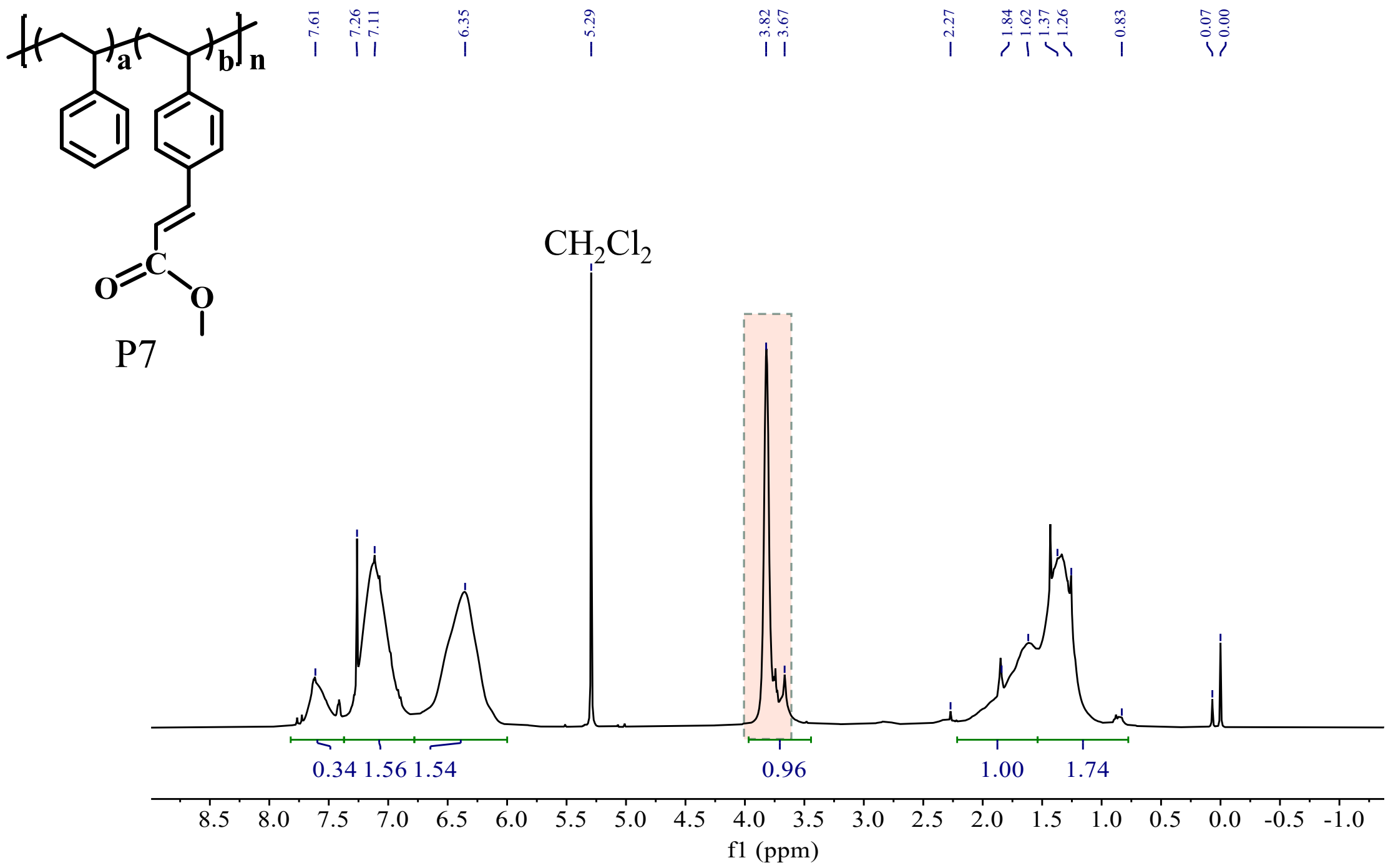

Figure S33. ^{1}H NMR (500 MHz, $CDCl_3$) spectrum of **P7**.

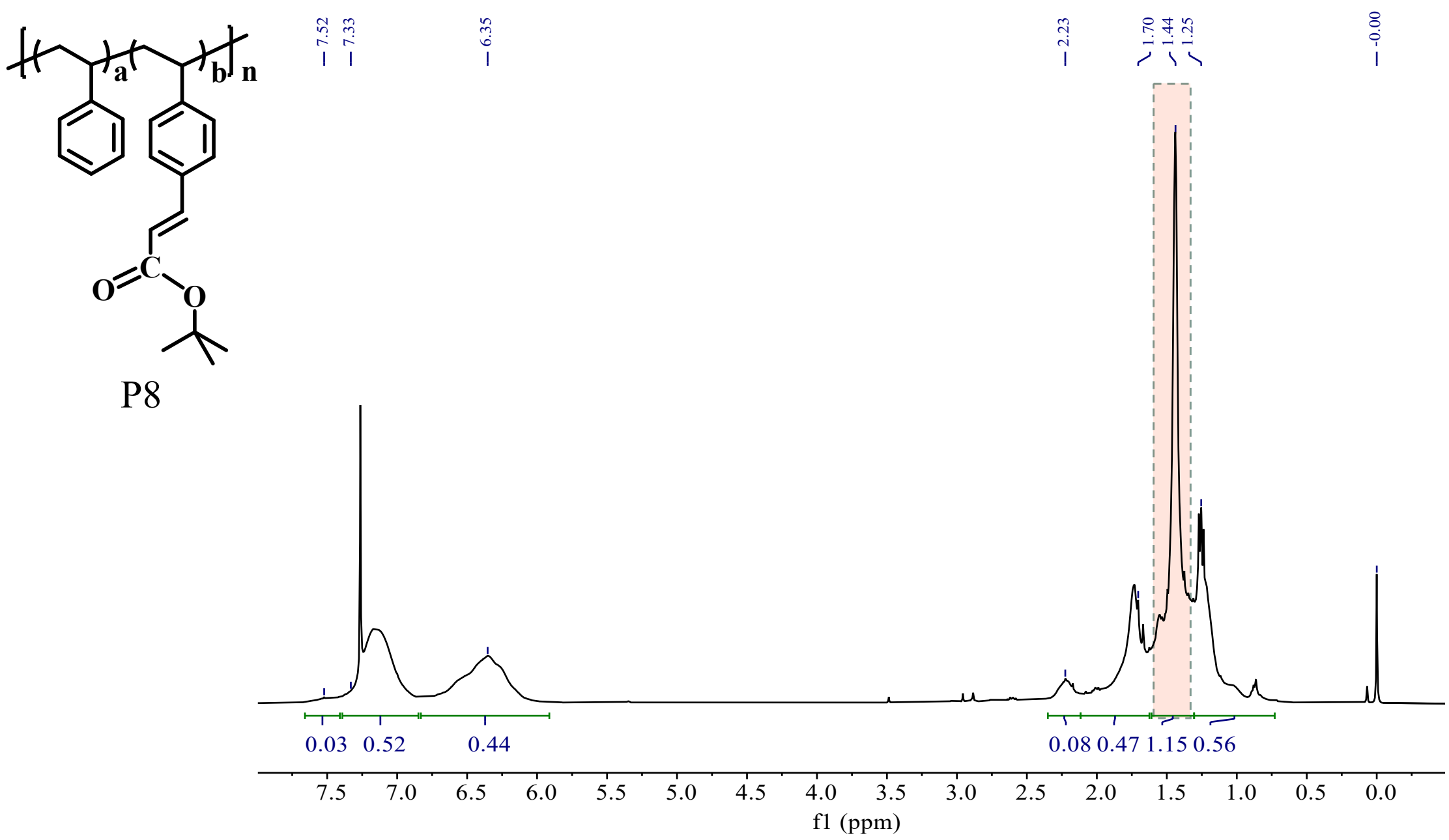

Figure S34. ^{1}H NMR (500 MHz, $CDCl_3$) spectrum of **P8**.

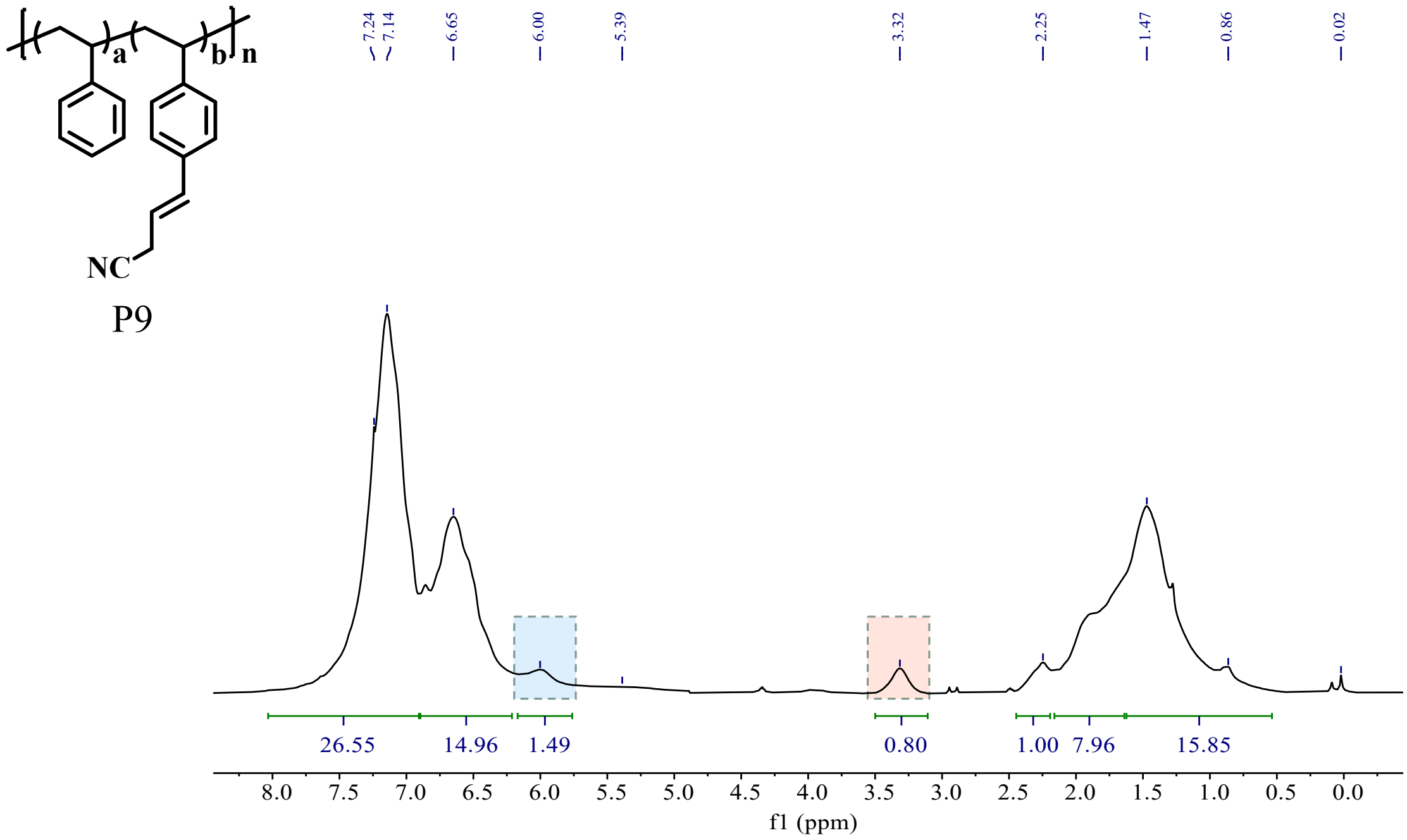

Figure S35. ^{1}H NMR (500 MHz, $CDCl_3$) spectrum of **P9**.

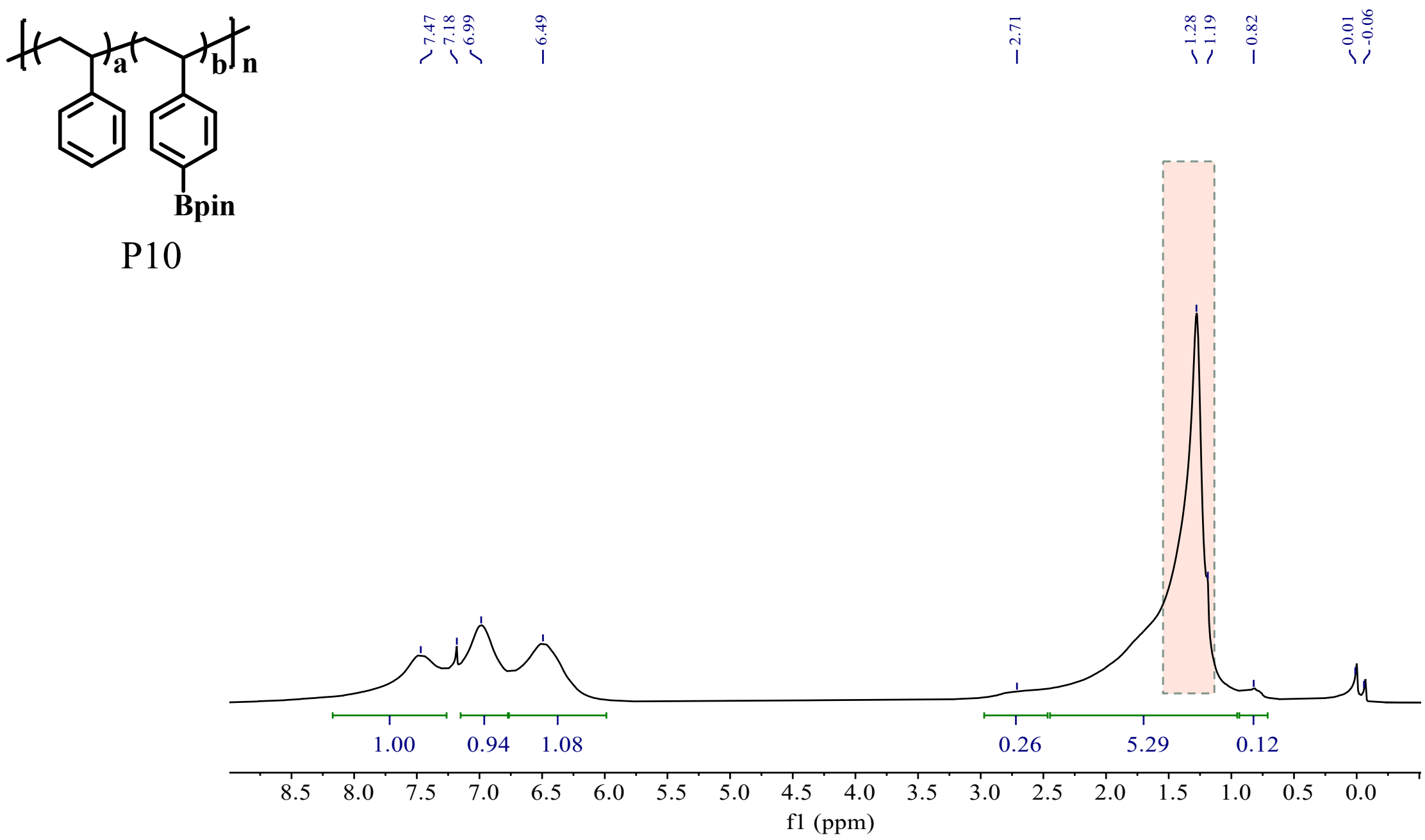

Figure S36. ^{1}H NMR (500 MHz, $CDCl_3$) spectrum of **P10**.

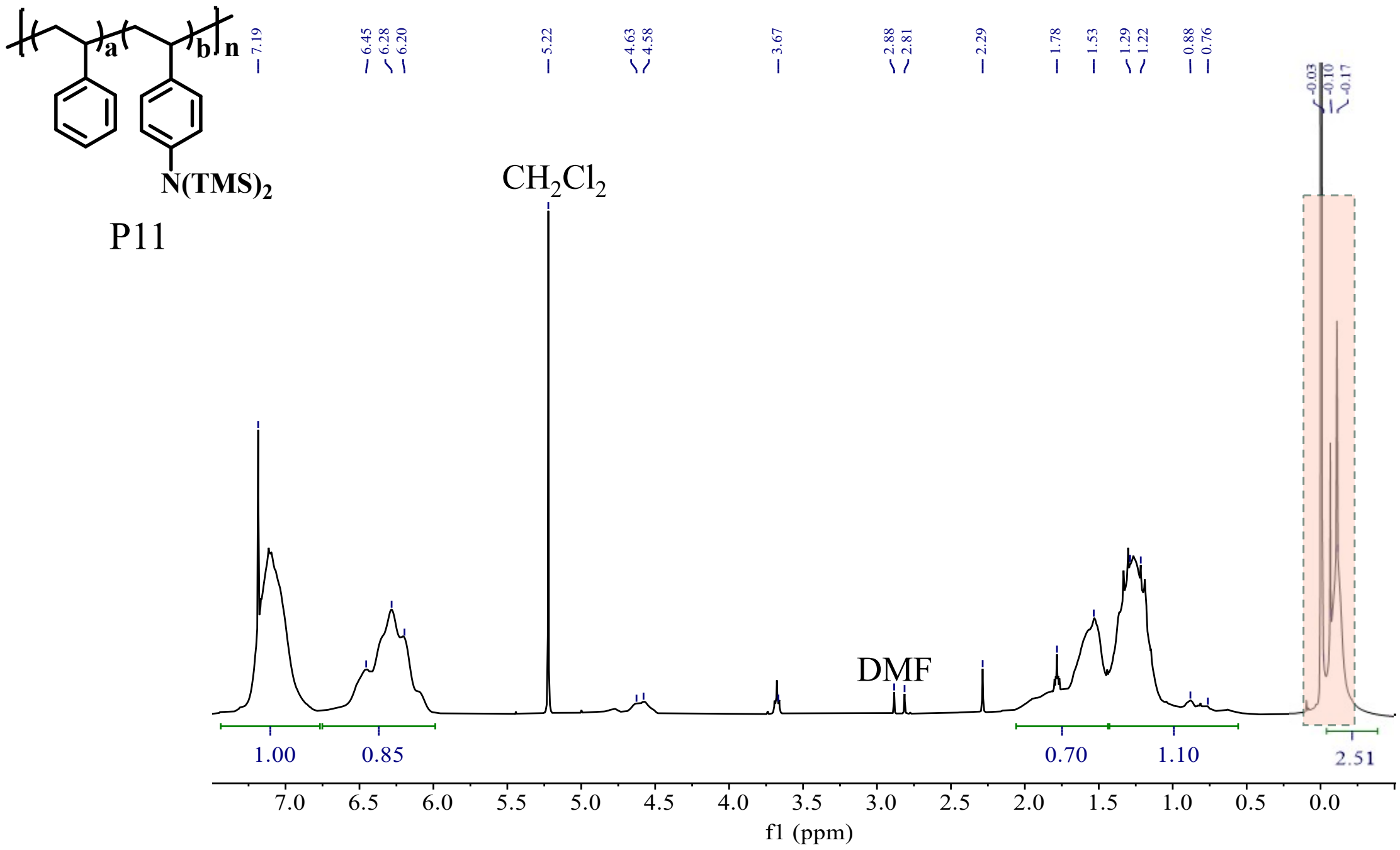

Figure S37. ^{1}H NMR (500 MHz, $CDCl_3$) spectrum of **P11**.

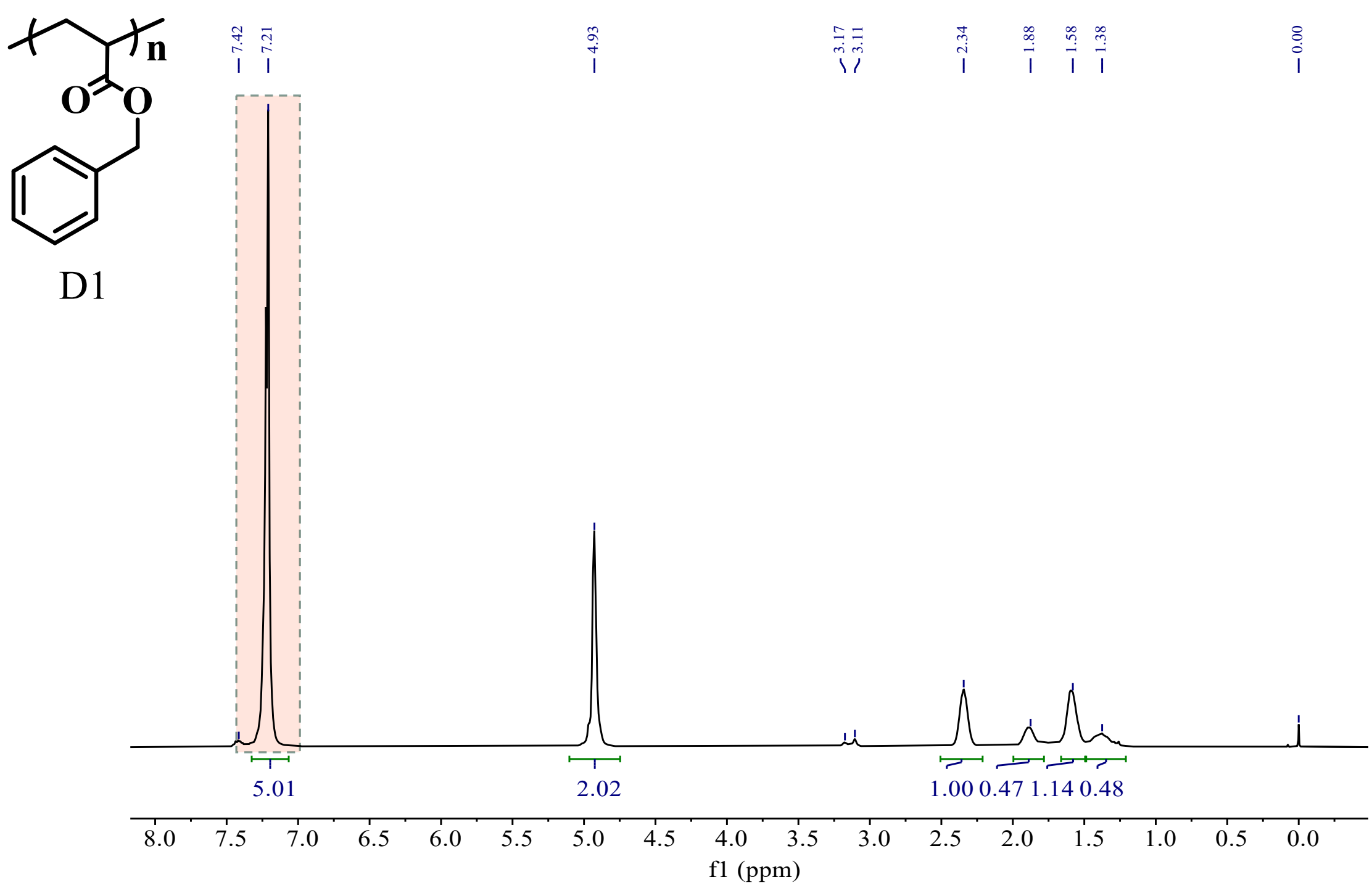

Figure S38. ^{1}H NMR (500 MHz, $CDCl_3$) spectrum of **D1**.

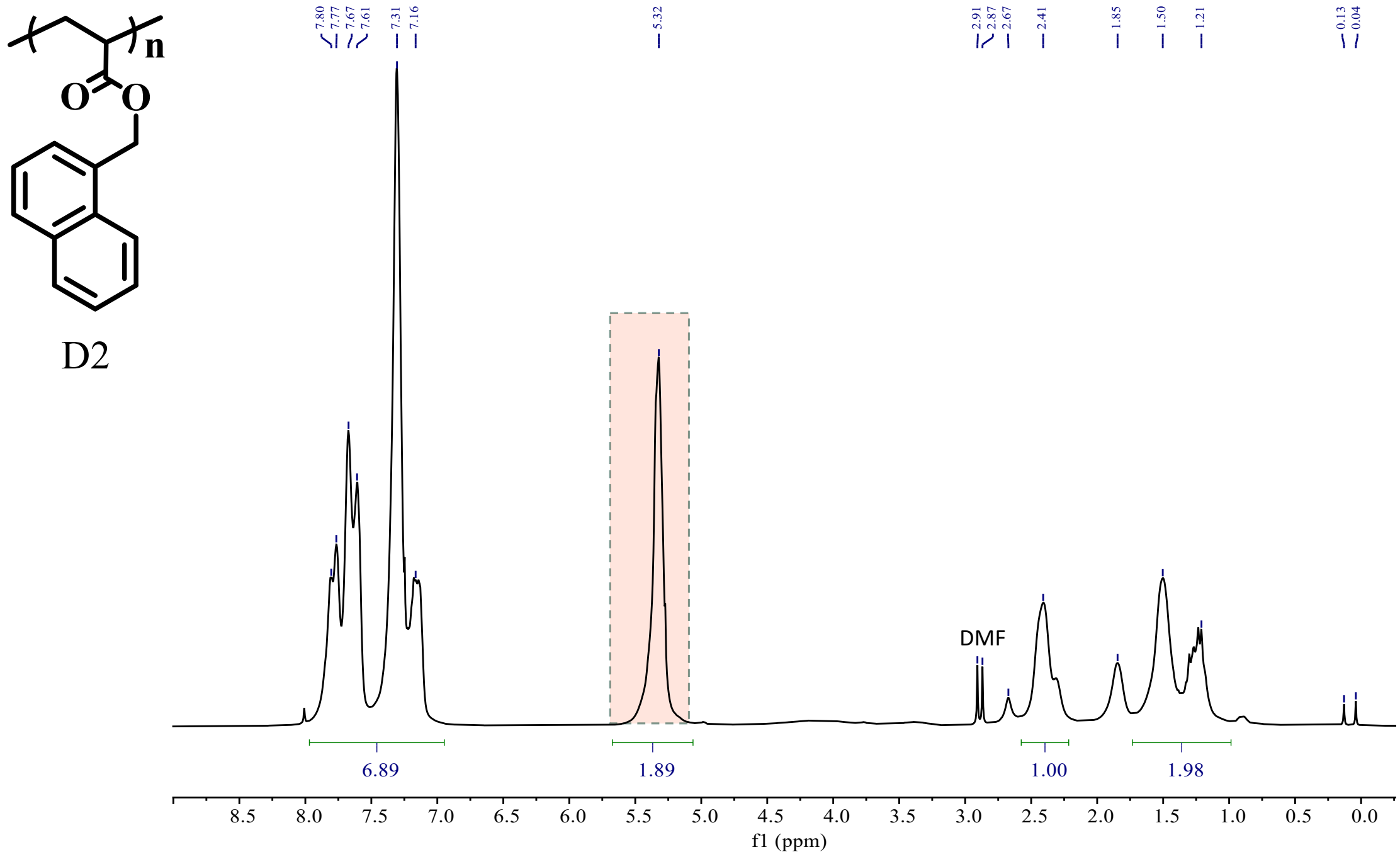

Figure S39. ^{1}H NMR (500 MHz, $CDCl_3$) spectrum of **D2**.

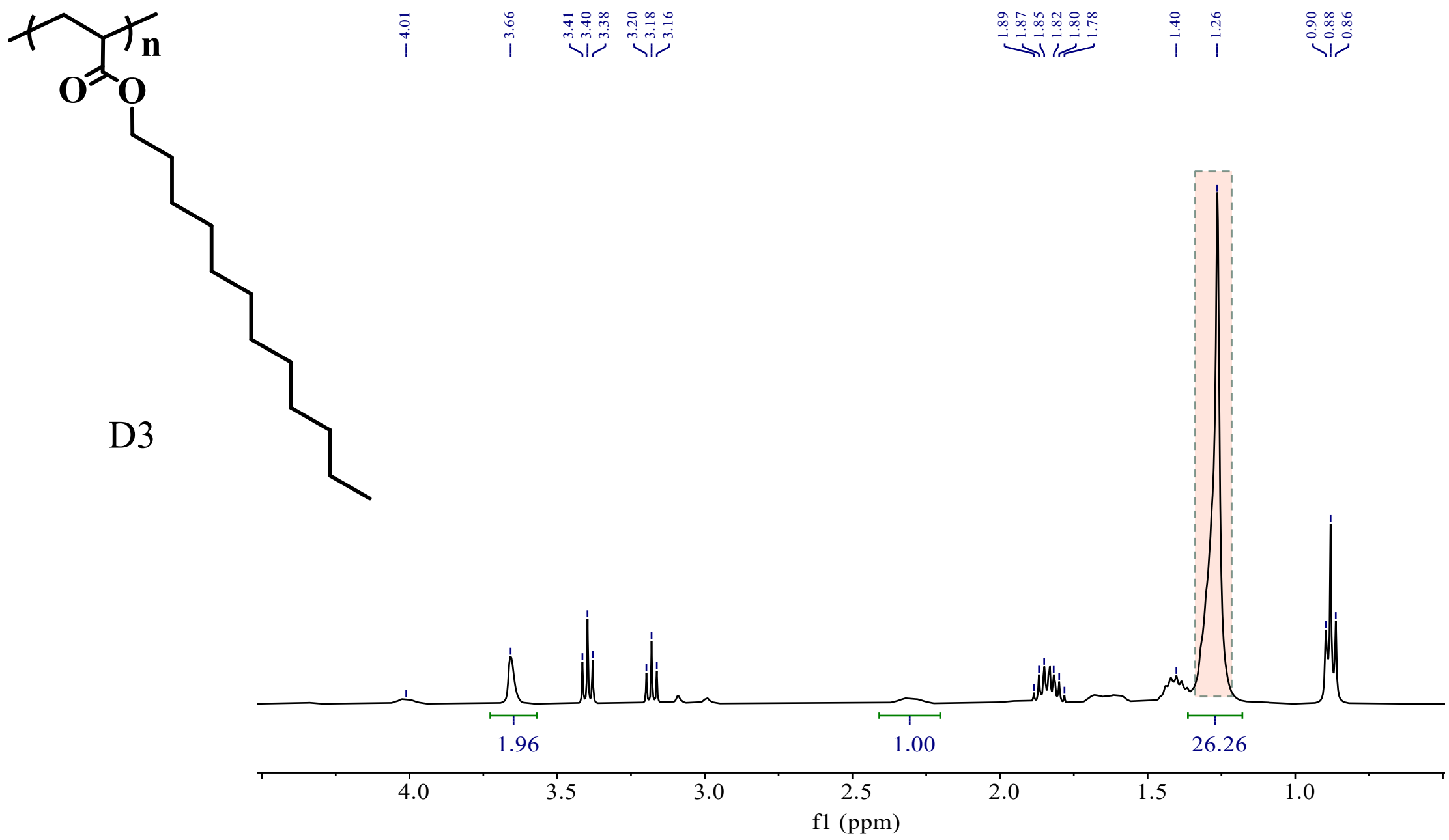

Figure S40. ^{1}H NMR (500 MHz, $CDCl_3$) spectrum of **D3**.

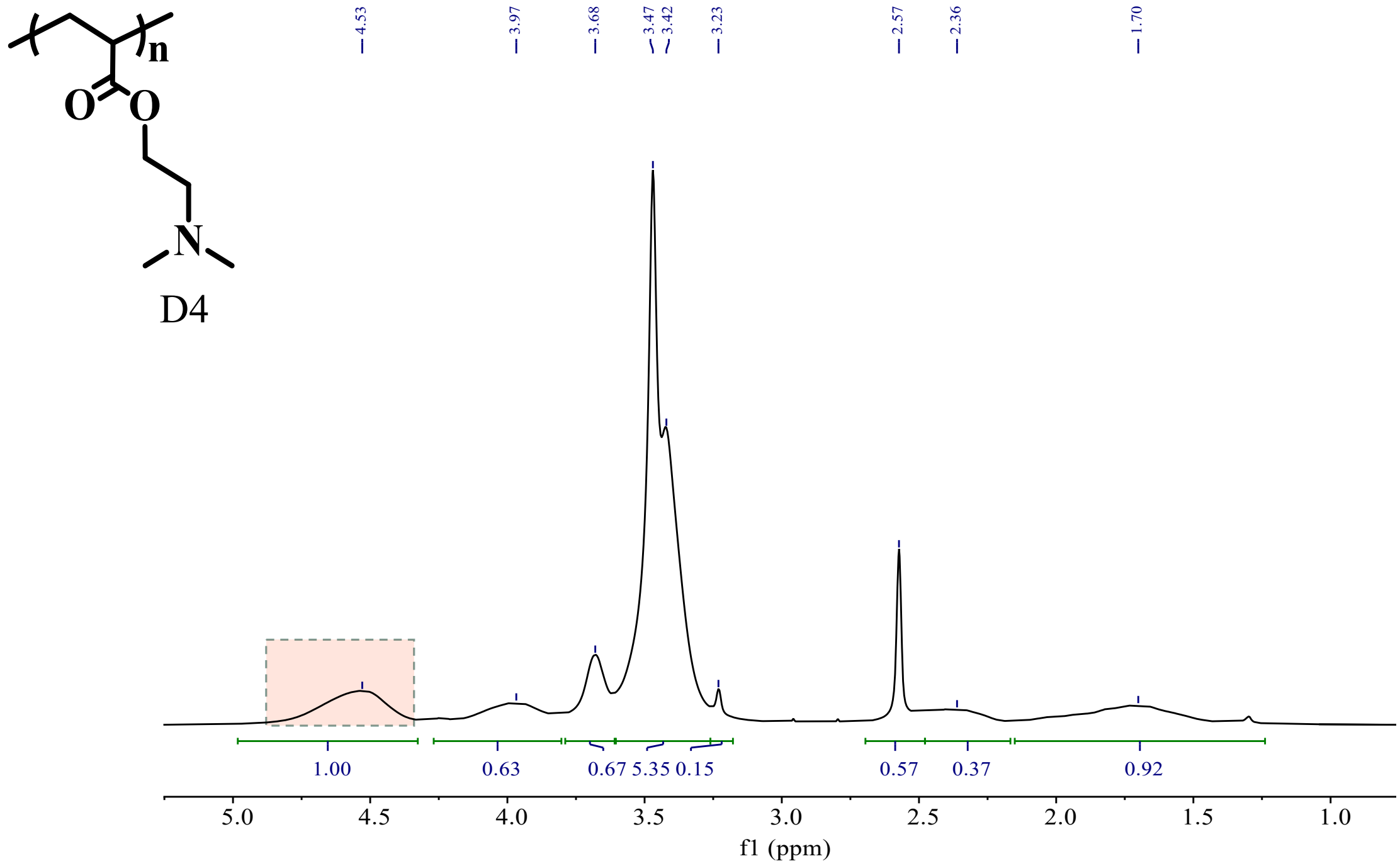

Figure S41. ^{1}H NMR (500 MHz, , DMSO-d_6) spectrum of **D4**.

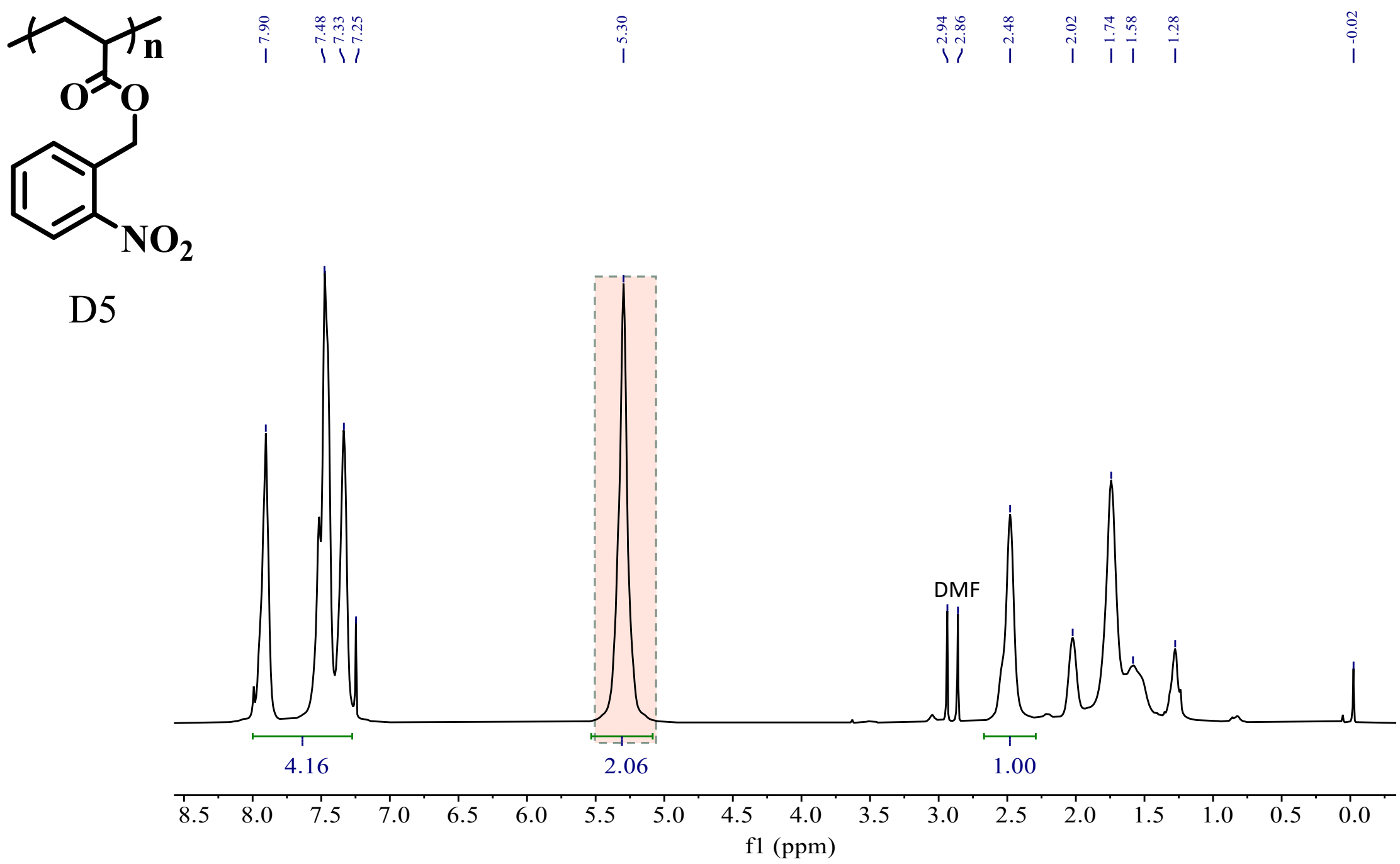

Figure S42. ^{1}H NMR (500 MHz, $CDCl_3$) spectrum of **D5**.

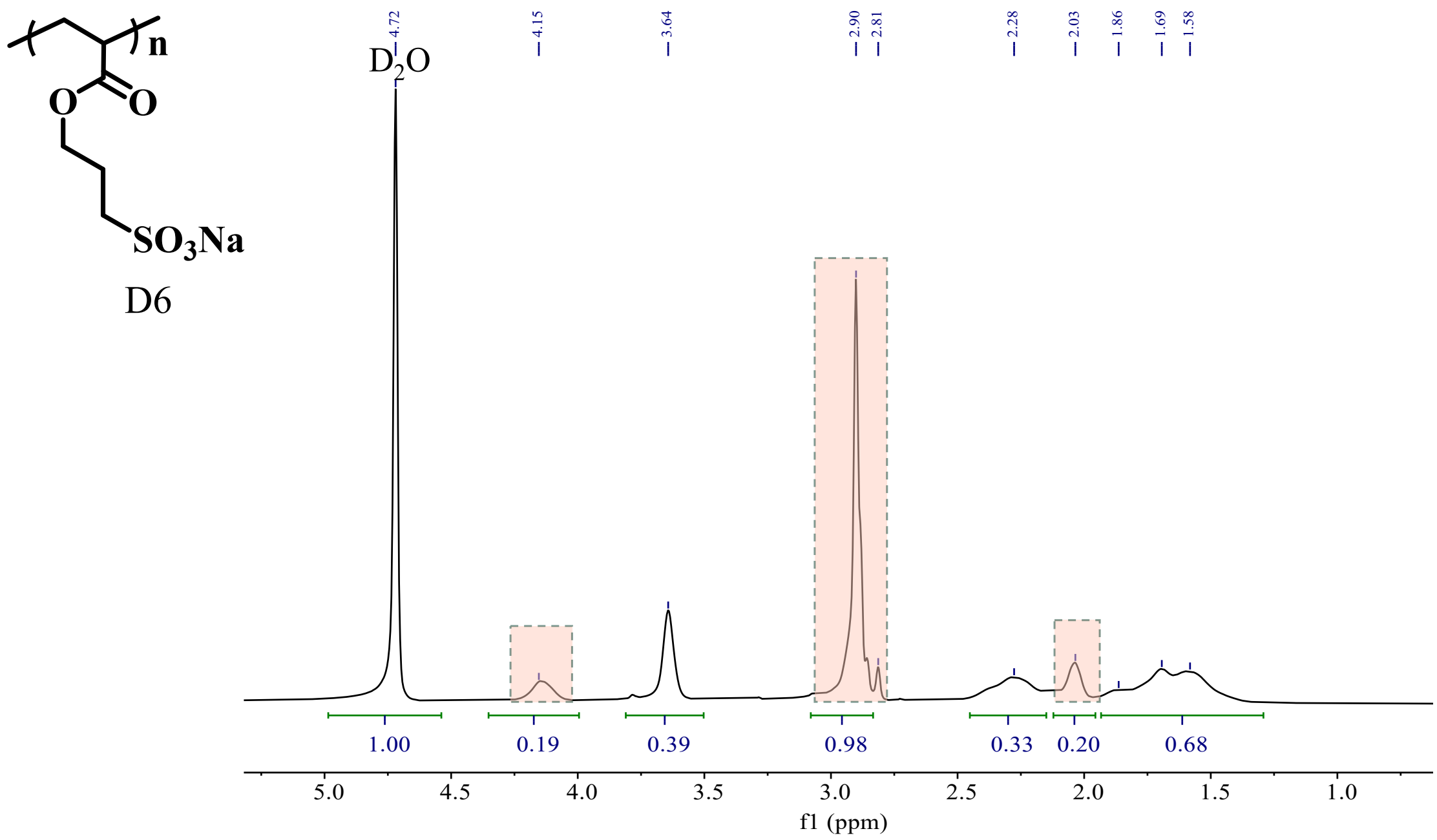

Figure S43. ^{1}H NMR (500 MHz, D_2O) spectrum of **D6**.

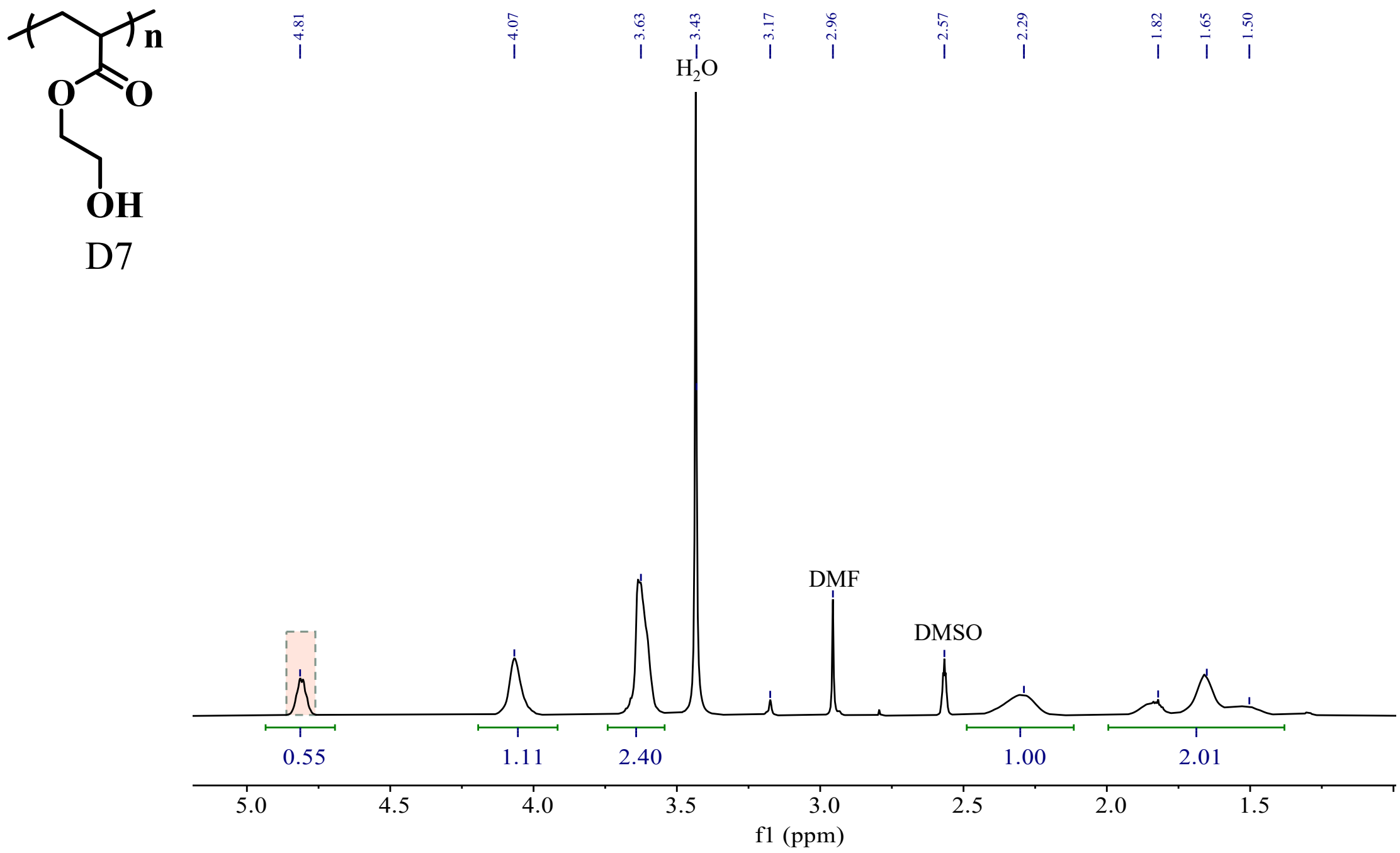

Figure S44. ^{1}H NMR (500 MHz, DMSO-d_6) spectrum of **D7**.

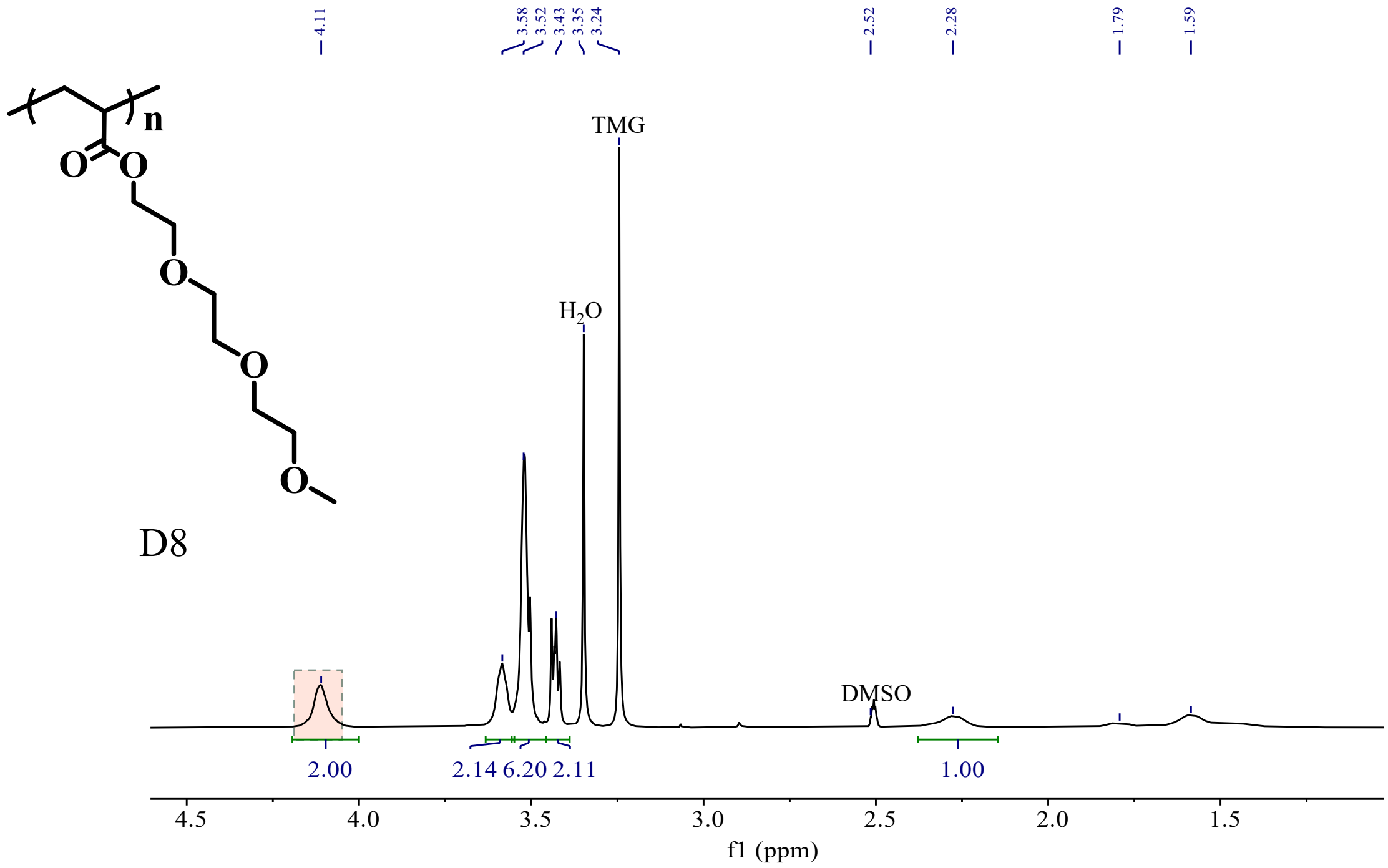

Figure S45. ^{1}H NMR (500 MHz, $CDCl_3$) spectrum of **D8**.

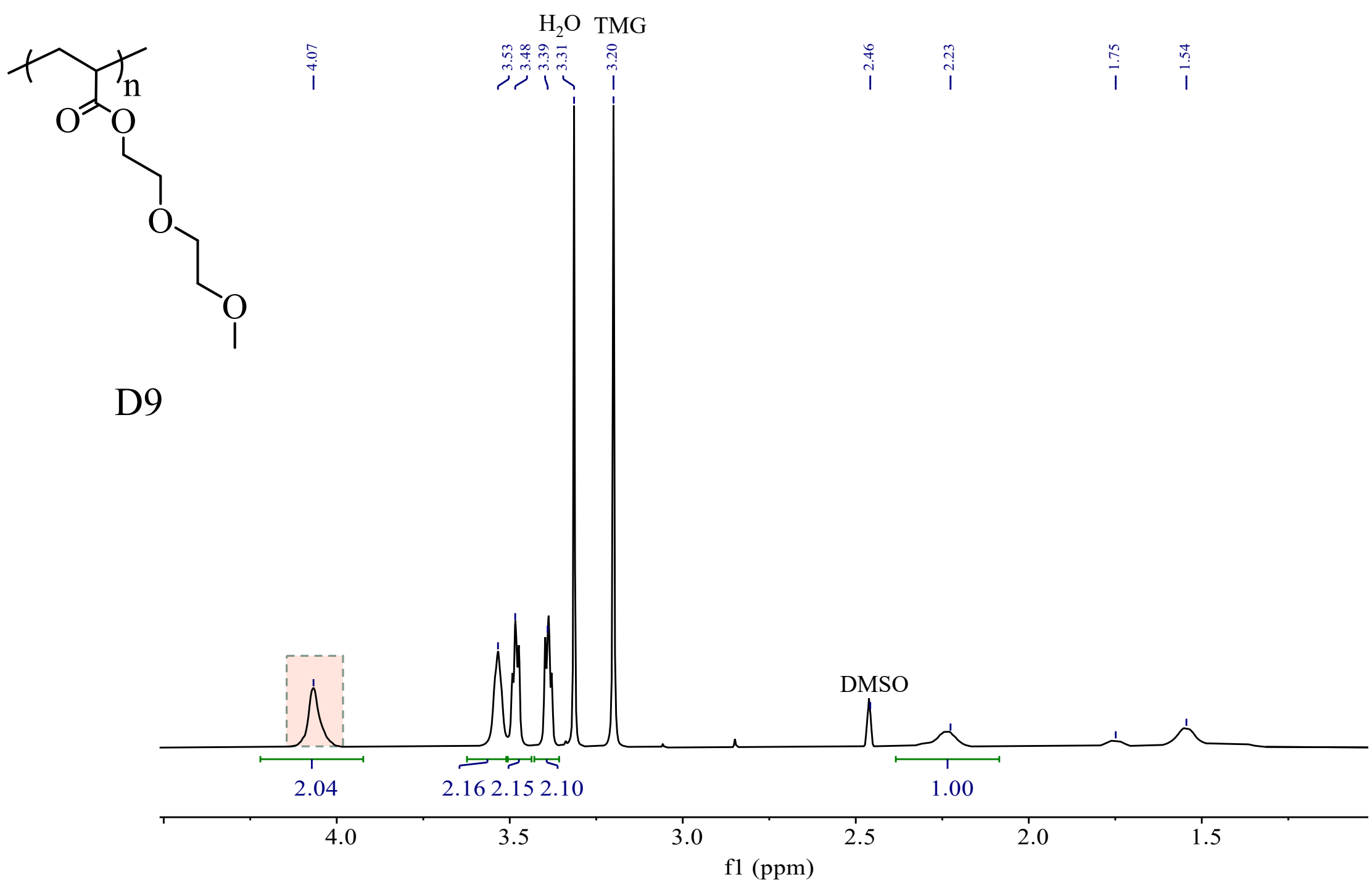

Figure S46. ^{1}H NMR (500 MHz, $CDCl_3$) spectrum of **D9**.

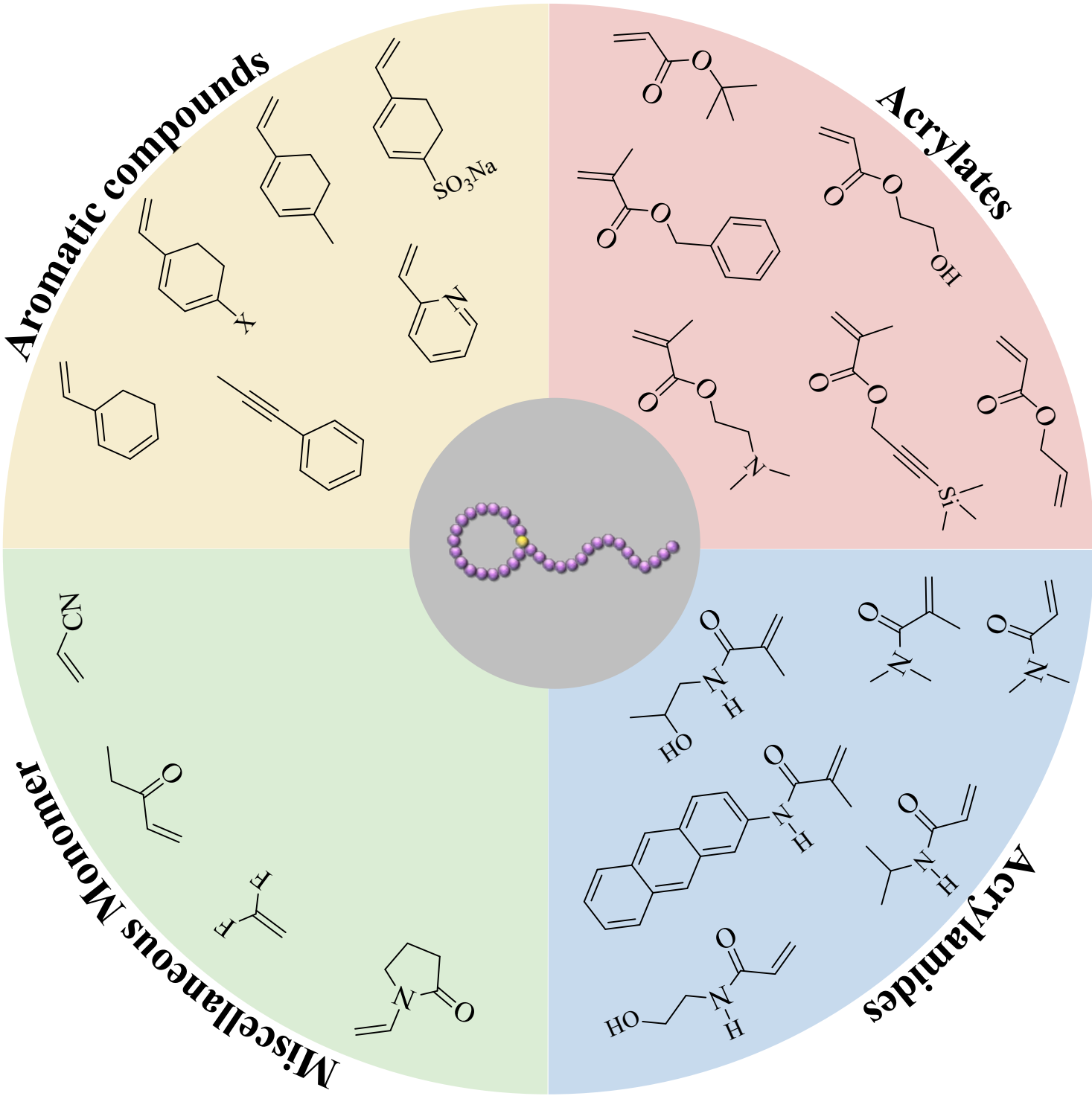

Figure S47. Preparation of potential tadpole polymers library based on dual-terminal intramolecular cyclization strategy.

Reference

(1) Li, L.; He, C.; He, W.; Wu, C. Formation Kinetics and Scaling of "Defect-Free" Hyperbranched Polystyrene Chains with Uniform Subchains Prepared from Seesaw-Type Macromonomers. *Macromolecules* 2011, 44, 8195–8206.

(2) Yang, J.; Chen, L.; Zhu, M.; Ishaq, M. W.; Chen, S.; Li, L. Investigation of the Multimer Cyclization Effect during Click Step-Growth Polymerization of AB-Type Macromonomers. *Macromolecules* 2022, 55, 6830–6840.

(3) Zhou, J.; Yang, J.; Ishaq, M. W.; Li, L. Study of Linear and Cyclic Graft Polystyrenes with Identical Backbone Contour in Dilute Solutions: Preparation, Characterization, and Conformational Properties. *Macromolecules* 2022, 55, 1398–1411.

(4) McGraw, M. L.; Clarke, R. W.; Chen, E. Y. X. Synchronous Control of Chain Length/Sequence/Topology for Precision Synthesis of Cyclic Block Copolymers from Monomer Mixtures. *J. Am. Chem. Soc.* 2021, 143, 3318–3322.

(5) Gao, L.; Oh, J.; Chang, T.; Chen, D.; Li, X.; Yang, X.; Tu, Y.; Zhu, X.; Li, C. Y. A Nearly Quantitative Synthetic Approach towards Monocyclic Polystyrenes and the Solvent, Concentration and Molecular Weight Effect on Cyclic Yield. *Polymer* 2016, 101, 379–387.

(6) Pan, X.; Ding, M.; Li, L. Experimental Validation on Average Conformation of a Comblike Polystyrene Library in Dilute Solutions: Universal Scaling Laws and Abnormal SEC Elution Behavior. *Macromolecules* 2021, 54, 11019–11031.

(7) Dou, B.; Xu, Y.; Wang, J. Gold-Catalyzed Precise Bromination of Polystyrene. *J. Am. Chem. Soc.* 2023, 145, 10422–10430.

(8) Matyjaszewski, K. Advanced Materials by Atom Transfer Radical Polymerization. *Adv. Mater.* 2018, 30, 1706441.

(9) Harrisson, S.; Whitfield, R.; Anastasaki, A.; Matyjaszewski, K. Atom Transfer Radical Polymerization. *Nat. Rev. Methods Primers* 2025, 5, 2.
